# Biomimetic synergistic effect of redox site and Lewis acid for construction of efficient artificial enzyme

**DOI:** 10.1038/s41467-024-50687-1

**Published:** 2024-07-26

**Authors:** Haibin Si, Dexin Du, Chengcheng Jiao, Yan Sun, Lu Li, Bo Tang

**Affiliations:** 1 College of Chemistry, Chemical Engineering and Materials Science, Collaborative Innovation Center of Functionalized Probes for Chemical Imaging, Key Laboratory of Molecular and Nano Probes, Ministry of Education, Shandong Normal University, Jinan, 250014 P. R. China; 2 Jinan Institute of Quantum Technology, Jinan, 250101 P. R. China; 3 Laoshan Laboratory, Qingdao, 266237 P. R. China

**Keywords:** Catalytic mechanisms, Bioinspired materials

## Abstract

In enzymatic catalysis, the redox site and Lewis acid are the two main roles played by metal to assist amino acids. However, the reported enzyme mimics only focus on the redox-active metal as redox site, while the redox-inert metal as Lewis acid has, to the best of our knowledge, not been studied, presenting a bottleneck of enzyme mimics construction. Based on this, a series of highly efficient M_x_V_2_O_5_·nH_2_O peroxidase mimics with vanadium as redox site and alkaline-earth metal ion (M^2+^) as Lewis acid are reported. Experimental results and theoretical calculations indicate the peroxidase-mimicking activity of M_x_V_2_O_5_·nH_2_O show a periodic change with the Lewis acidity (ion potential) of M^2+^, revealing the mechanism of redox-inert M^2+^ regulating electron transfer of V-O through non-covalent polarization and thus promoting H_2_O_2_ adsorbate dissociation. The biomimetic synergetic effect of redox site and Lewis acid is expected to provide an inspiration for design of enzyme mimics.

## Abstract (web summary)

The so far reported enzyme mimics focus on the redox-active metal as a redox site, while the redox-inert metal as Lewis acid has so far not been reported. Here, the authors report efficient M_x_V_2_O_5_·nH_2_O peroxidase mimics with vanadium as redox site and alkaline-earth metal ion (M^2+^) as Lewis acid, where the peroxidase-mimicking activity shows a periodic change with the Lewis acidity of the metal.

## Introduction

Enzyme is a kind of high-performance catalyst in biology, which controls most chemical reactions under physiological conditions^1,2^. It is important to simulate the structure and function of enzymes for the development of efficient enzyme-mimicking catalysts. In terms of structure, amino acids are the main body of enzymes, leading enzymes to catalyze reactions^3^. But in many cases, amino acids alone are not enough to handle all enzymatic reactions. In these cases, non-amino acid components can bind to the protein, thereby improving the reaction dynamics. Metal ions are present in nearly a third of all enzymes as cofactors, helping to catalyze biochemical reactions and perform specific physiological functions^4^. The role of metal ions in the enzymatic catalytic mechanism can be roughly divided into two groups, depending on whether the metal acts as a redox site in the catalytic process^5^. When the metal (redox-active metal ion) functions as a redox site, it is usually directly involved in one or more steps of an enzymatic reaction by obtaining/providing electrons from/to other reactive substances. Such as copper in superoxide dismutase^6,7^, iron in peroxidase^8,9^. When the metal (redox-inert metal ion) functions as a non-redox site, it acts as a Lewis acid to reduce the activation energy by activating the reaction substrate and stabilizing the intermediate or transition state. Such as magnesium in 6-hydroxymethyl-7,8-dihydropterin pyrophosphokinase, calcium in phosphoinositide phospholipase C^10,11^.

**Fig. 1:**
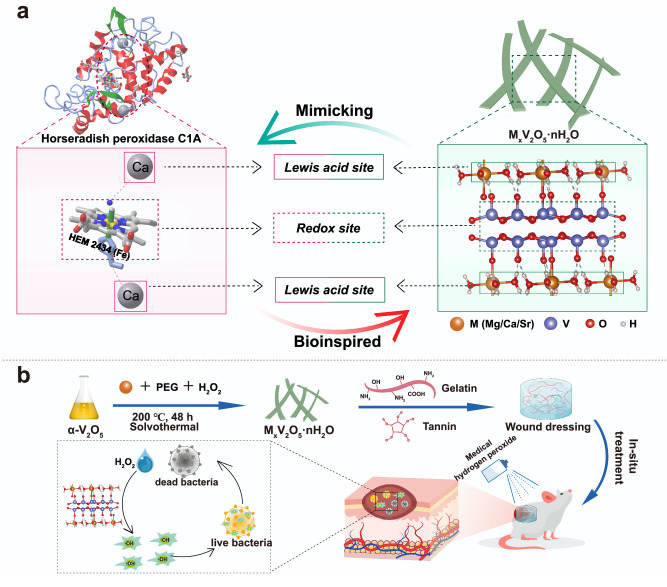
Design peroxidase mimics with synergetic effect of redox-active vanadium and Lewis acidic alkali earth metal. **a** Schematic illustration of the active site of natural horseradish peroxidase C1A (HRP C1A), in which Fe^3+^ is located in protoporphyrin IX as redox site in coordination with the proximal histidine ligand and 2 mol Ca^2+^ as Lewis acid site at the proximal and distal sites of redox site. The alkaline-earth metal M (M = Mg, Ca, Sr) and the active center V are connected by non-covalent bonds in bioinspired M_x_V_2_O_5_·nH_2_O, which resemble the Lewis acid site and the redox site in HRP C1A, respectively. **b** Bioinspired M_x_V_2_O_5_·nH_2_O serves as the peroxidase mimics against bacteria for accelerating infected wound healing.

The heme peroxidase superfamily, isolated from many plants, fungi, and bacteria, is a group of enzymes that use hydrogen peroxide to oxidize the reducing substrate^12,13^ Horseradish peroxidase isoenzyme C (HRP-C), as the most studied isoenzyme of the peroxidase superfamily, its structure and function are typical^14,15^. It is generally acknowledged that the peroxidase contains high-spin Fe^3+^, located in protoporphyrin IX in coordination with the proximal histidine ligand. As the redox center of peroxidase, Fe^3+^ functions primarily by transferring electrons during enzymatic catalysis. At the same time, there is also redox-inert Ca^2+^ in HRP-C with a content of 2 mol of Ca^2+^/mol of enzyme. The Ca^2+^ are on both sides of the protoporphyrin plane, which do not directly participate in electron transfer, but can significantly affect the catalytic activity of enzyme (left part of Fig. 1a). Haschke R H, Friedhoff J M. et al. found that after the removal of Ca^2+^ in HRP-C, more than half of the activity of HRP-C could be lost^16,17^. Further study by Isao Morishima et al. showed that the removal of Ca^2+^ significantly change the redox properties of HRP, and the redox potential of Fe(III)/Fe(II) experienced a cathodic shift of 30 mV. This may be due to the loss of Ca^2+^ affects the structure of the imidazolyl ligand of the redox iron^13,18^. The similar results also be verified in wheat and barley peroxidase^19–21^. In addition, cationic peroxides extracted from the Mediterranean shrub Euphorbia characias have one strongly bound endogenous Ca^2+^ ^22^. Removal of Ca^2+^ resulted in changes in the electronic structure of heme iron, and the activity of the Ca-free enzyme was about 2% of that of the natural enzyme. Moreover, the second Ca^2+^ added make the enzyme efficiency parameter k_cat_/K_m_ was nearly 1000 times higher than that of native enzyme. These studies indicate that redox-inert metal ions play a key role in regulating the conformation and electronic structure of peroxidase.

Due to the important role of the metal ion in natural enzyme, many different types of metal-based nanomaterials, such as transition metal oxide nanomaterials^23,24^, noble metal nanomaterials^25,26^, metal organic frameworks (MOFs)^27^, and single-atom catalysts^28–30^, had been designed and synthesized for enzyme-mimicking catalysis. The metals in these enzyme mimics are usually transition elements with different valence states. These redox-active metal ions can achieve electron transfer in the catalytic process, which are a good simulation of the redox center in natural enzyme^28^. However, the enzyme mimics using redox-inert metal ions as Lewis acid inspired from natural enzymes have not been formally proposed and systematically explored. Considering the key role of redox-inert metal ions in adjusting the geometric and electronic structure of enzymes, the synergism of redox-active metal ions and redox-inert metal ions is expected to provide an inspiration for the design and synthesis of highly efficient enzyme-mimicking materials.

Inspired by the structure of natural peroxidase, the bionic synergetic structure with redox site and Lewis acid should have the following three characteristics: redox-active metal with high electron transfer ability as the main body of the enzyme-mimicking nanomaterials; redox-inert metal as Lewis acid site; the redox-inert metals exist in the main body of redox-active metal by weak bonding and have an effect on the geometric and electronic structure of the main body. Vanadium oxides such as V_2_O_5_^31,32^, VO_2_^33^, V_6_O_13_^34^ etc., have been reported to have good peroxidase-mimicking activities, which mainly based on the multi-electron transfer ability of element V as redox center. In addition, vanadium oxides are a class of layered materials with highly anisotropic bonding, which are strongly bonded via covalent interaction within layers and stacked by weak van der Waals interactions between layers. The interlayer space allows for the intercalation of different guest species, such as cations, anions, organic molecules, etc., thus achieving structural and functional regulation^35–37^. Therefore, the layered vanadium oxide is an ideal material for constructing the bionic synergetic structure.

In this paper, with vanadium oxides (V_2_O_5_) as host material, introducing redox-inert alkaline-earth metal ion (M = Mg, Ca, Sr) guest between layers through intercalation, we constructed a series of M_x_V_2_O_5_·nH_2_O peroxidase mimics with a biomimetic synergistic structure of redox-active metal (redox site) and redox-inert metal (Lewis acid) (right part of Fig. 1a). The characterization show that M_x_V_2_O_5_·nH_2_O have a unique layered crystal structure with large interlayer spacing, a mixed oxidation state with high V^4+^/ (V^4+^ + V^5+^) ratio, and abundant oxygen vacancies (O_V_). M_x_V_2_O_5_·nH_2_O exhibited more significant peroxidase-mimicking activity compared with other typical peroxidase mimics, even some noble metal and single atom materials. Structural analysis, experimental results and theoretical calculations demonstrate that the excellent enzyme-mimicking activity of M_x_V_2_O_5_·nH_2_O can be ascribed to the increase of O_V_ concentration caused by the change of coordination structure and the enhancement of H_2_O_2_* dissociation ability caused by the change of electronic structure after redox-inert M^2+^ intercalation. Among them, this paper found that the enzyme-mimicking activity of M_x_V_2_O_5_·nH_2_O is based on the periodic change of Lewis acidity (ion potential) of intercalated M^2+^, revealing the mechanism of the redox-inert M^2+^ regulating electron transfer of V-O through non-covalent polarization and thus promoting OH* formation. Furthermore, M_x_V_2_O_5_·nH_2_O nanobelts were used as antibacterial additives in hydrogel as wound dressings due to their stable banded structure and high hydroxyl radical generation capacity. In vivo and in vitro experimental data show the wounding dressings containing M_x_V_2_O_5_·nH_2_O nanobelts possess the ability of good broad-spectrum antibacterial and promoting wound healing.

## Results and discussion

### **Synthesis and characterization**

**Fig. 2:**
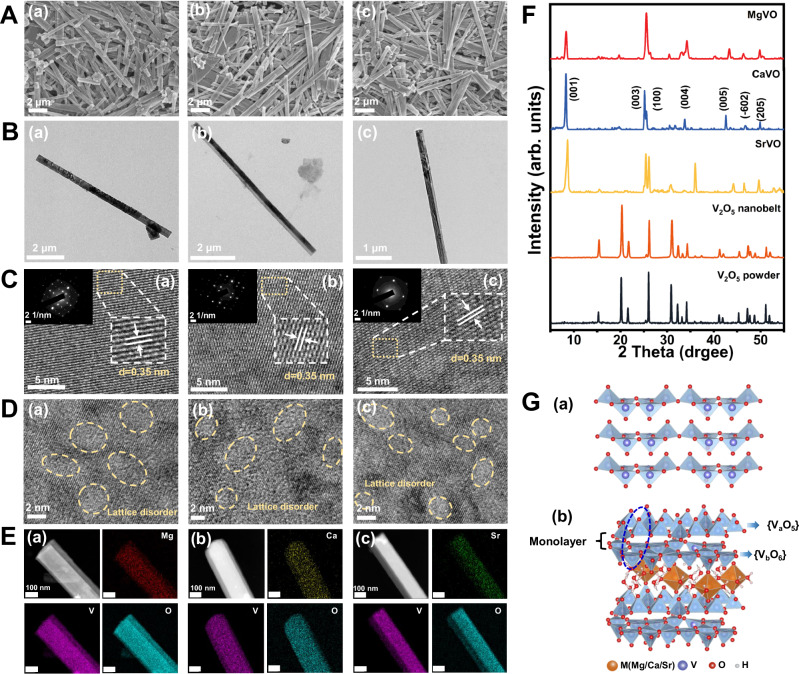
Synthesis and characterization of M_x_V_2_O_5_·nH_2_O nanobelts. **A** SEM images, **B** TEM images, **C** HRTEM images (the inserts are SAED images) with clear lattice fringes and lattice disorder **D**, EDX-mapping (**E**) of MgVO (a), CaVO (b) and SrVO (c). **F** XRD patterns of MgVO, CaVO, SrVO, V_2_O_5_ nanobelt and V_2_O_5_ powder. **G** Crystal structure of α-V_2_O_5_ (a) and M_x_V_2_O_5_·nH_2_O (b). Representative images are shown from three independent experiments with similar results (**A-E**).

M_x_V_2_O_5_·nH_2_O (M = Mg, Ca, Sr) was prepared by a simple reducing hydrothermal method^38^. Using commercially available V_2_O_5_ powder as precursor, then H_2_O_2_ was used as a reducing agent to reconstruct the crystal structure of V_2_O_5_ for the intercalation of different alkaline-earth metal ions by reacting with MgCl_2_·6H_2_O, CaCl_2_, SrCl_2_·6H_2_O, respectively. In addition, the V_2_O_5_ nanobelt without intercalation as control sample was also as control samples prepared by same methods without adding alkaline-earth metal ions to investigate the effect of M^2+^ intercalation on the structure and properties of V_2_O_5_. Scanning transmission microscope (SEM) and transmission electron microscopy (TEM) images showed that V_2_O_5_ powder were irregular particle with 1–3 μm (Supplementary Fig. 1A, 1 C). The V_2_O_5_ nanobelt exhibits unique 1D structure with length of 1 to 5 μm and width of 10 to 100 nm (Supplementary Fig. 1B, D). The lengths of M_x_V_2_O_5_·nH_2_O are between 1 to 10 μm and the widths are between 200 to 450 nm (Fig. 2A, B, Supplementary Fig. 2, 3). In addition, atomic force microscope (AFM) images show that the thicknesses of most M_x_V_2_O_5_·nH_2_O materials are in the range of 20–60 nm (Supplementary Fig. 4). Due to their large aspect ratio and nanoscale thickness, the M_x_V_2_O_5_·nH_2_O materials are also defined as M_x_V_2_O_5_·nH_2_O nanobelts. By high resolution transmission electron microscopy (HRTEM) analysis, the identical lattice spacing of Mg_x_V_2_O_5_·nH_2_O, Ca_x_V_2_O_5_·nH_2_O and Sr_x_V_2_O_5_·nH_2_O nanobelts is 0.35 nm, corresponding to (100) lattice (Fig. 2C). It is worth noting that lattice disorder and mismatching can be observed in the HRTEM images of three kinds of M_x_V_2_O_5_·nH_2_O (Fig. 2D), which may come from the surface oxygen vacancies (O_V_) introduced by M^2+^ intercalation. The concentration and formation of vacancies will be discussed in the subsequent experiments and characterization. The insets in Fig. 2C shows the selected area electron diffraction (SAED) images of M_x_V_2_O_5_·nH_2_O. The uniform and well-ordered space lattices suggest the single-crystal diffraction pattern. Energy-dispersive X-ray spectroscopy (EDX) showed the existence and uniform distribution of M, V, and O in M_x_V_2_O_5_·nH_2_O, and no element segregation was found when compared with HRTEM images (Fig. 2E). The chemical composition of M_x_V_2_O_5_·nH_2_O nanobelts were also investigated by inductively coupled plasma mass spectrometry (ICP-MS) and thermogravimetric analysis (TGA), and the results are shown in Supplementary Table 1 and Supplementary Fig. 5. The molecular formulas of the three kinds of M_x_V_2_O_5_·nH_2_O are Mg_0.22_V_2_O_5_·0.75H_2_O (MgVO), Ca_0.30_V_2_O_5_·0.83H_2_O (CaVO), Sr_0.34_V_2_O_5_·0.72H_2_O (SrVO), respectively.

The crystalline phases of V_2_O_5_ powder, V_2_O_5_ nanobelt, and M_x_V_2_O_5_·nH_2_O nanobelts were characterized by X-ray diffraction (XRD) to further verify the preparation of V_2_O_5_ nanobelt and M_x_V_2_O_5_·nH_2_O nanobelts. As shown in Fig. 2F, the XRD pattern of V_2_O_5_ powder matches the standard orthorhombic V_2_O_5_ (α-V_2_O_5,_ JCPDS no. 41-1426, space group Pmmn). α-V_2_O_5_ has a typical two-dimensional (2D) layered structure, which facilitates metal ion insertion between layers (Fig. 2G). V_2_O_5_ nanobelt has similar crystal structure with V_2_O_5_ powder without crystal phase change. The XRD pattern of CaVO is well matched with the monoclinic phase Ca_0.24_V_2_O_5_·H_2_O (JCPDS no. 01-088-0579), which is a kind of layered material containing Ca^2+^ and coordination water. Its cell parameters are as follows: a = 11.68 Å, b = 3.65 Å, c = 10.999 Å, β = 105.41°. No standard cards matching the XRD patterns of MgVO and SrVO were found, but the diffraction peaks corresponding to (001), (003), (004) and (005) crystal planes in the XRD patterns of the three kinds of M_x_V_2_O_5_·nH_2_O were similar, indicating that they have similar crystal structures. In particular, compared with V_2_O_5_ powder and V_2_O_5_ nanobelt, sharp diffraction peaks before 2θ = 10° appeared in M_x_V_2_O_5_·nH_2_O (Fig. 2F, Supplementary Fig. 6). The diffraction peaks near 2θ = 10° are generally attributed to the (001) crystal plane, which indicate that the crystal displays lamellar ordering as dominated by the pronounced (001) reflections^39^. With the intercalation of M^2+^ and H_2_O in M_x_V_2_O_5_·nH_2_O, the lattice spacing of (001) increases, causing the diffraction peak of (001) shift to a smaller angle^40^. By substituting the above diffraction peaks into Bragg equation 2dsinθ = nλ, the crystal plane spacing of MgVO, CaVO and SrVO can be calculated as 10.54 Å, 10.66 Å, 10.23 Å, respectively. Compared with V_2_O_5_ (4.37 Å)^41^, the larger crystal plane spacing further indicated the successful insertion of Mg^2+^, Ca^2+^, Sr^2+^. The corresponding structure diagrams according to the XRD patterns and related crystal parameters are drawn in Fig. 2G. It can be seen from the schematic diagram that the α-V_2_O_5_ layer is composed of VO_5_ square pyramids. However, square pyramids are connected up and down by sharing apexes point. Thus, if oxygen from adjacent square pyramids is included, the vanadium coordination geometry can be described as a distorted octahedra belonging to the saturated coordination^42^. M_x_V_2_O_5_·nH_2_O crystallized in the monoclinic space group *C*2/*m*. When α-V_2_O_5_ is converted to monoclinic M_x_V_2_O_5_·nH_2_O, the V-O-V bilayers are separated into two adjacent V-O-V monolayers due to the elongated V-O distances. It has been shown that this is caused by the pre-intercalation of hydrated metal ions and free water molecular chains. Each V-O-V monolayer in M_x_V_2_O_5_·nH_2_O is composed of {V_a_O_5_} tetragonal pyramids and {V_b_O_6_} octahedrons, extending infinitely in the plane (Fig. 2G). In the interlayer of V-O-V bilayers, M^2+^ coordinates with four water molecules to form planar square geometries, sharing edges with each other in a 1D chain. It can be seen that some V centers and M centers in M_x_V_2_O_5_·nH_2_O show unsaturated coordination, indicating that there may be abundant oxygen vacancies in M_x_V_2_O_5_·nH_2_O^39,43^. V_2_O_5_ nanobelt has no obvious crystal structure change compared with V_2_O_5_ powder. This suggests that metal intercalation may be able to effectively induce crystal structure change and oxygen vacancies generation, which is also consistent with related HRTEM characterization. It has been reported that transition metal center with unsaturated coordination have high catalytic activity, and abundant oxygen vacancies can provide adsorption sites for catalytic reactions^44,45^, indicating that metal ion intercalation may bring additional catalytic activity to M_x_V_2_O_5_·nH_2_O.

**Fig. 3:**
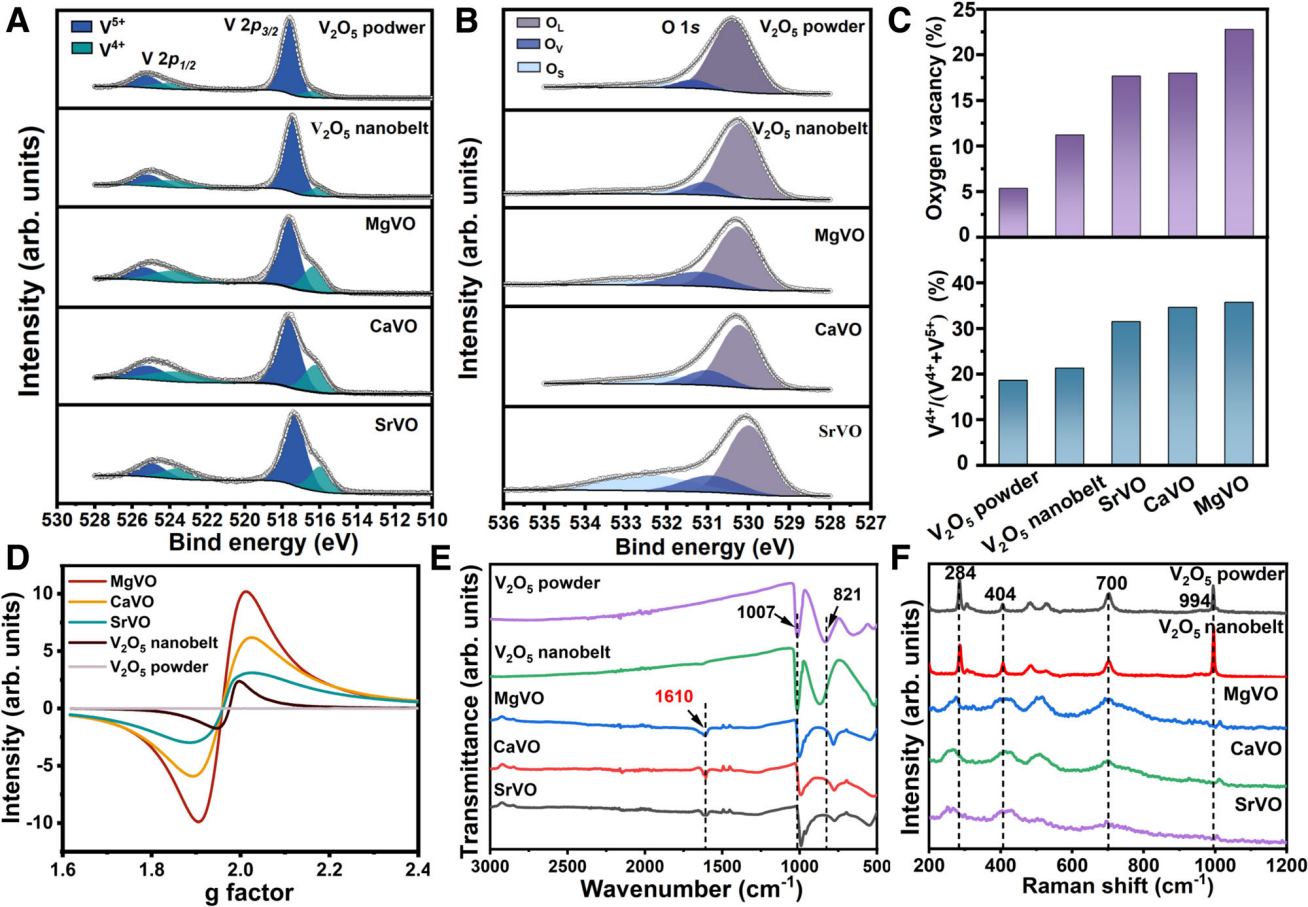
Characterization of M_x_V_2_O_5_·nH_2_O nanobelts. **A** XPS spectra of V 2*p* and **B** O 1 *s* of, V_2_O_5_ powder, V_2_O_5_ nanobelt, MgVO, CaVO, and SrVO. **C** Quantitative analysis of the V^4+^/(V^4+^ + V^5+^) ratio and relative content of O_V_ of V_2_O_5_ powder, V_2_O_5_ nanobelt, MgVO, CaVO, and SrVO. **D** The analysis of O_V_ of V_2_O_5_ powder, V_2_O_5_ nanobelt, MgVO, CaVO, and SrVO by ESR spectroscopy. **E** FTIR spectra (1007 cm^−^^1^: symmetric stretching vibration of V = O; 821 cm^−^^1^: stretching vibration of V-O-V; 1610 cm^−^^1^: vibration of O-H) and (**F**) Raman spectra of V_2_O_5_ powder, V_2_O_5_ nanobelt, MgVO, CaVO, and SrVO. (994 cm^−^^1^: symmetric stretching vibration of V = O; 700 and 404 cm^−^^1^: stretching vibration of V-O; 284 cm^−^^1^: vibration of V = O).

To further understand the elemental composition and vacancies information of the prepared materials, X-ray photoelectron spectroscopy (XPS) analysis (Supplementary Fig. 7) was performed on V_2_O_5_ powder, V_2_O_5_ nanobelt and M_x_V_2_O_5_·nH_2_O nanobelts. Compared with V_2_O_5_ powder and V_2_O_5_ nanobelt, M_x_V_2_O_5_·nH_2_O nanobelts show new peaks at 1304.2 eV, 345.7 eV, and 351.0 eV, which corresponding to Mg 1 *s*, Ca 2*p*_*3/2*_, Ca 2*p*_*1/2*_ and Sr 3*d*_*3/2*_*/3d*_*5/2*_, respectively. This further confirmed the successful intercalation of M^2+^ in M_x_V_2_O_5_·nH_2_O nanobelts. The five materials all show two similar characteristic peaks between 512–528 eV, corresponding to the spin orbit peaks of V 2*p*_*3/2*_ and V 2*p*_*1/2*_ respectively. Compared with V_2_O_5_ powder, the V 2*p*_*3/2*_ and V 2*p*_*1/2*_ of M_x_V_2_O_5_·nH_2_O nanobelts show prominent asymmetry, indicating that V exists in a larger V^4+^/(V^4+^ + V^5+^) ratio in M_x_V_2_O_5_·nH_2_O nanobelts (Fig. 3A). The deconvolution of the peaks show that the proportion of V^4+^/(V^4+^ + V^5+^) in M_x_V_2_O_5_·nH_2_O nanobelts reach 35.7% 34.6% and 31.5%, respectively, significantly higher than 18.6% in V_2_O_5_ powder, indicating that more V^5+^ are reduced to V^4+^ during the generation of M_x_V_2_O_5_·nH_2_O nanobelts (Fig. 3C). However, the V^4+^/ (V^4+^ + V^5+^) ratio in V_2_O_5_ nanobelt with the same dose of reducing agent added in the preparation process is only 21.3%, meaning that the insertion of M^2+^ is conducive to the existence of high V^4+^/(V^4+^ + V^5+^) ratio. The relative content of oxygen vacancies in V_2_O_5_ nanobelt and M_x_V_2_O_5_·nH_2_O nanobelts were further analyzed based on O 1 *s* high resolution spectra. As shown in Fig. 3B, the O 1 *s* spectrum can be fitted to three peaks of 529.5 eV, 530.5 eV, and 532 eV, which belong to the oxygen vacancies and surface-adsorbed oxygen (O_S_) and lattice oxygen (O_L_), respectively. As expected, the relative content of O_V_ in M_x_V_2_O_5_·nH_2_O nanobelts reach 22.76%, 17.96%, and 17.65% with the increase of V^4+^/(V^4+^ + V^5+^) ratio respectively, which higher than V_2_O_5_ powder (5.34%) and V_2_O_5_ nanobelt (11.2%) (Fig. 3C). The reconstruction of the structure and the generation of O_V_ are further confirmed by the use of electron spin resonance (ESR) spectra. As shown in Fig. 3D, M_x_V_2_O_5_·nH_2_O nanobelts transmit an intense resonance signal at g = 1.97, corresponding to the captured electrons in O_V_, whereas only a weak signal is detected in V_2_O_5_ powder and V_2_O_5_ nanobelt. The results of ESR are consistent with those of XPS, and verified with HRTEM and XRD. In order to further understand the bonding structure of the prepared M_x_V_2_O_5_·nH_2_O nanobelts, Fourier transform infrared (FT-IR) spectroscopy and Raman spectroscopy were used to analyze them. The attributions of major characteristic peaks are shown in Fig. 3E, F, which is consistent with relevant reports^46^.

### Highly specific and efficient enzyme-mimicking activity of M_x_V_2_O_5_·nH_2_O nanobelts

**Fig. 4:**
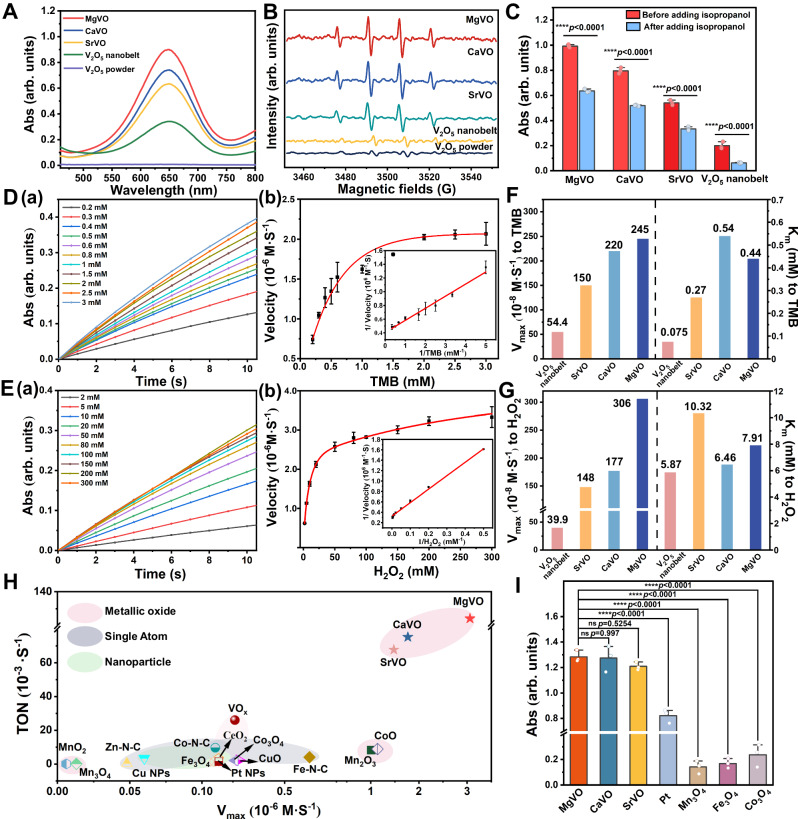
Detection of enzyme-mimicking activity and steady-state kinetics of M_x_V_2_O_5_·nH_2_O. **A** UV-vis absorption spectra of oxTMB in the presence of H_2_O_2_ with M_x_V_2_O_5_·nH_2_O, V_2_O_5_ powder, V_2_O_5_ nanobelt, respectively. **B** The generation of ·OH testified by ESR spectra of M_x_V_2_O_5_·nH_2_O, V_2_O_5_ powder, V_2_O_5_ nanobelt in the presence of H_2_O_2_. **C** Absorbance of oxTMB in the presence of H_2_O_2_ and M_x_V_2_O_5_·nH_2_O/V_2_O_5_ nanobelt after adding isopropanol. *n*  =  3 experimental replicates. Data are presented as mean values  ±  SD. The data were analyzed by using a one-sided unpaired *t*-test. ****P < 0.0001. **D** Time-dependent absorbance of oxTMB (a), reaction rate (b) with different concentrations of TMB and fixed concentrations of MgVO/H_2_O_2_, and the corresponding double reciprocal (Lineweaver-Burk) plots (inset). **E** Time-dependent absorbance of oxTMB (a), reaction rate (b) with different concentrations of H_2_O_2_ and fixed concentrations of MgVO/TMB, and the corresponding double reciprocal (Lineweaver-Burk) plots (inset). *n*  =  3 experimental replicates (**D**, **E** (b)). Data are presented as mean values  ±  SD (**D**, **E** (b)). V_max_ and K_m_ values of M_x_V_2_O_5_·nH_2_O/V_2_O_5_ nanobelt towards to TMB (**F**) and (**G**) H_2_O_2_. **H** The comparison of the TON and V_max_ values of M_x_V_2_O_5_·nH_2_O with other recently reported catalysts. **I** The comparison of the relative catalytic activity of M_x_V_2_O_5_·nH_2_O and synthesized peroxidase mimics. *n*  =  3 experimental replicates. Data are presented as mean values  ±  SD. Data were analyzed by one-way ANOVA with Turkey’s multiple comparisons test, ns represented no statistical difference, ****P < 0.0001.

The M_x_V_2_O_5_·nH_2_O nanobelts prepared in this paper are expected to present excellent catalytic performance due to the unsaturated coordination of vanadium and abundant oxygen vacancies caused by M^2+^ intercalation. The peroxidase-mimicking activity of M_x_V_2_O_5_ nanobelts were studied by using 3, 3’, 5, 5’-tetramethylbenzidine (TMB), 2, 2’-azino-bis (3-ethylbenzothiazoline-6-sulfonic acid) (ABTS) and o-Phenylenediamine (OPD) as substrates for typical color reaction in the presence of H_2_O_2_. As shown in Fig. 4A, M_x_V_2_O_5_·nH_2_O nanobelts can effectively catalyze the oxidation of TMB in the presence of H_2_O_2_, showing obvious peroxidase-mimicking activity. Experiments also show that M_x_V_2_O_5_·nH_2_O nanobelts exhibit optimal peroxidase-mimicking catalytic activity under weak acidic environment (pH=5.0) and near room temperature (10–80 °C) (Supplementary Fig. 8). The M_x_V_2_O_5_·nH_2_O nanobelts maintain stable crystal structures and morphologies within 15 days at pH=5.0 (Supplementary Fig. 9, 10). Correspondingly, V_2_O_5_ powder show no detectable peroxidase-mimicking activity, while V_2_O_5_ nanobelt without M^2+^ intercalation show significantly lower catalytic activity than M_x_V_2_O_5_·nH_2_O nanobelts under the same conditions. These results indicate that the activated peroxidase-mimicking activity of M_x_V_2_O_5_·nH_2_O nanobelts may be related to the composition and structure changes after M^2+^ intercalation. The above results were also confirmed when ABTS and OPD were used as substrates (Supplementary Fig. 11). Meanwhile, cyclic voltammetry was used to verify the catalytic activity of M_x_V_2_O_5_·nH_2_O on the substrate^26,47^. As shown in Supplementary Fig. 12, 13, when H_2_O_2_ and TMB are added to the electrolyte, the currents corresponding to the redox of V with different valence states are significantly reduced, showing obvious catalytic responses. In the absence of H_2_O_2_, M_x_V_2_O_5_·nH_2_O nanobelts can not effectively catalyze the oxidation of TMB/ABTS/OPD, indicating that it has almost no oxidase-mimicking activity (Supplementary Fig. 14). In addition, the dissolved oxygen test and the nitrogen blue tetrazole (NBT) colorimetry showed that M_x_V_2_O_5_·nH_2_O nanobelts show no obvious catalase-mimicking activity and superoxide dismutase-mimicking activity (Supplementary Fig. 14). In addition, previous studies have shown that some vanadium-based nanozymes also demonstrate haloperoxidase and glutathione peroxidase activities^48,49^. Therefore, we detected the haloperoxidase-mimicking activity and glutathione peroxidase-mimicking activity of M_x_V_2_O_5_·nH_2_O. As shown in Supplementary Fig. 15 and 16, M_x_V_2_O_5_·nH_2_O show glutathione-mimicking peroxidase activity, and no significant haloperoxidase-mimicking activity was detected.

According to the peroxidase catalytic mechanism, the ability of M_x_V_2_O_5_·nH_2_O to catalyze the decomposition of H_2_O_2_ to produce ·OH was verified by ESR spectra. Due to the short life and high chemical activity of ·OH, 5, 5-dimethyl-1-pyrrolin-n-oxide (DMPO) was used as ·OH radical catcher to evaluate the production of ·OH. As shown in Fig. 4B, in the presence of H_2_O_2_, the ESR spectra of M_x_V_2_O_5_·nH_2_O show an obvious characteristic spectroscopy of 1:2:2:1 intensity for DMPO/·OH adducts, which proved the generation of ·OH in the M_x_V_2_O_5_·nH_2_O/H_2_O_2_ system. To further verify the generation of ·OH, isopropyl alcohol as ·OH scavenger was added to the M_x_V_2_O_5_·nH_2_O/H_2_O_2_/TMB catalytic reaction system, the absorbance of oxTMB in the reaction system decreased significantly (Fig. 4C), illustrating ·OH is the main reactive species in the enzymatic reaction system of M_x_V_2_O_5_·nH_2_O.

In order to evaluate the peroxidase-mimicking activity of M_x_V_2_O_5_·nH_2_O nanobelts, the steady-state kinetic analysis of M_x_V_2_O_5_·nH_2_O was carried out in proper concentration range of TMB and H_2_O_2_. Figure 4D, E and Supplementary Fig. 17–19 show the absorption spectra of catalytic oxidation of TMB by M_x_V_2_O_5_·nH_2_O at different concentrations of TMB or H_2_O_2_. The XPS analysis of M_x_V_2_O_5_·nH_2_O was performed before and after the reaction. It can be seen from Supplementary Fig. 20 that M_x_V_2_O_5_·nH_2_O remain relatively stable under catalytic conditions. According to the initial reaction rate calculated in Fig. 4D (a), 4E (a), typical Michaelis-Menten curves of TMB and H_2_O_2_ can be obtained respectively (Fig. 4D (b), 4E(b)). Next, the reciprocal of substrate concentration and initial reaction rate could be used to prepare the double reciprocal plot (Lineweaver-Burk) to obtain the maximal reaction velocity (V_max_) and Michaelis−Menten constant (K_m_). Among them, V_max_ is an important parameter to evaluate the catalytic rate of enzymatic reaction, and K_m_ is an important indicator of enzyme affinity to substrates. As shown in Fig. 4F, when TMB was used as substrate, V_max_ of MgVO, CaVO and SrVO nanobelts are 245, 220, 150 (10^−8^ M·s^−^^1^), K_m_ are 0.44, 0.54, 0.27 mM, respectively; When H_2_O_2_ was used as substrate (Fig. 4G), the V_max_ of MgVO, CaVO and SrVO nanobelts are 306, 177, 148 (10^−8^ M·s^−^^1^), and K_m_ are 7.91, 6.46, 10.32 mM, respectively. Comparing with natural horseradish peroxidase (HRP) and classical Fe_3_O_4_ (Supplementary Table 2), the K_m_ of M_x_V_2_O_5_·nH_2_O for the two substrates are similar to that of HRP and Fe_3_O_4_, but the V_max_ of M_x_V_2_O_5_·nH_2_O are more than one order of magnitude higher than that of HRP and Fe_3_O_4_ under their respective optimal catalytic conditions. In order to systematically evaluate its catalytic performance, we compared the V_max_ and TON values (the maximum number of conversing substrates via the mole concentration of metal in the whole nanomaterials^50^) of M_x_V_2_O_5_·nH_2_O with currently reported classical peroxidase mimics, including Zn-N-C^51^, Pt NPs^52^, CeO_2_^53^, Fe_3_O_4_^53^, and MnO_2_^53^, etc (Supplementary Table 3), the results indicate M_x_V_2_O_5_·nH_2_O display the optimal catalytic performances in comparison with metal oxides-based nanozymes, even some noble metal nanozymes and single atom nanozymes (Fig. 4H). In order to eliminate data differences caused by different test conditions, four typical peroxidase-mimicking materials were synthesized by referring to the methods in the literature (Supplementary Fig. 21, 22), and catalytic oxidation of TMB was tested under the same test conditions. The results of Fig. 4I directly confirm that M_x_V_2_O_5_·nH_2_O nanobelts presented high efficiency peroxidase-mimicking activity.

### The synergistic effect of redox V and redox-inert M in enzyme-mimicking catalysis

Theoretical calculations and some experimental studies have showed that the catalytic reaction paths of POD-like nanozymes can be classified as two types according to dissociation mode of H_2_O_2_ adsorbate (H_2_O_2_*)^54,55^. The path 1 is that the dissociated H_2_O_2_* generates hydroxyl adsorbate (OH*) and hydroxyl radical (·OH), then generates H_2_O and oxidized substrate to complete the cycle^30,51,56–58^. Because it is similar to the well-known Fenton reaction, this mechanism is also known as the Fenton-like mechanism and is widely accepted to explain the POD-like activity of many materials. The path 2 usually does not involve the formation of ·OH. H_2_O_2_* first generates two OH*, which can directly oxidize the substrate under acidic conditions^59,60^. Alternatively, OH* can also be converted to O* and H_2_O* through a hydrogen transfer reaction, with O* oxidizing the substrate^53,57,61,62^. This path that the adsorbed ROS directly acting on substrates has attracted more and more attention because it is similar to the catalytic mechanism of natural peroxidase (ferryl oxo species). In view of the direct detection of ·OH produced under catalysis by ESR spectroscopy and the scavenging verification ·OH by isopropyl alcohol, the possible catalytic mechanism of M_x_V_2_O_5_·nH_2_O is proposed following path 1. Therefore, the reaction mechanism was studied from the free energy, charge density difference, and band structure through density functional theory calculation. Subsequently, combined with the changes in the structure and catalytic activity of M_x_V_2_O_5_·nH_2_O after M^2+^ intercalation, the synergistic effect of redox-inert M^2+^ with redox-active V is further discussed.

**Fig. 5:**
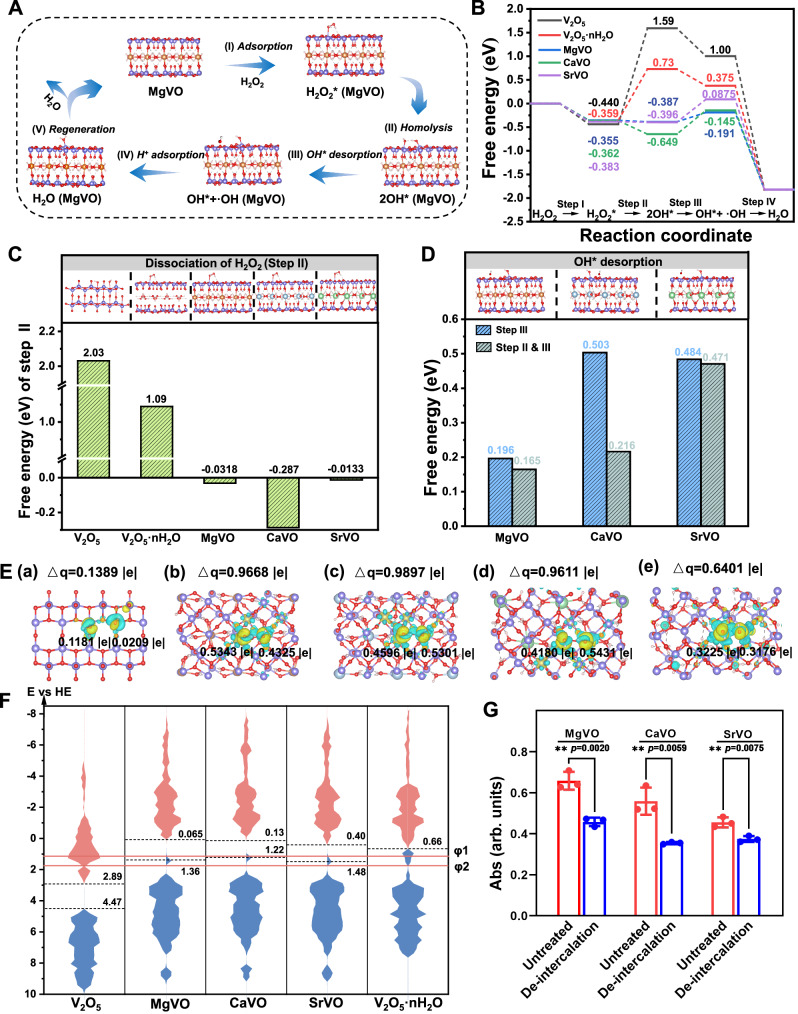
DFT calculations and the proposed mechanism for the peroxidase-mimicking of M_x_V_2_O_5_·nH_2_O. **A** The possible reaction pathway on M_x_V_2_O_5_·nH_2_O, taking MgVO for example. (Purple: V atom; Red: O atom; White: H atom; Yellow: Mg atom). **B** Free energy diagram of the proposed reaction pathways for V_2_O_5_, V_2_O_5_·nH_2_O and M_x_V_2_O_5_·nH_2_O models. **C** Free energy change of dissociation of H_2_O_2_ (Step II) on different models. (Blue, Ca atom; Green: Sr atom). **D** Free energy change of OH* desorption (Step III or Step II & III) on M_x_V_2_O_5_·nH_2_O models. **E** The differential charge analysis of (a) V_2_O_5_, (b) MgVO, (c) CaVO, (d) SrVO and (e) V_2_O_5_·nH_2_O with 2OH* models (cyan and yellow represent charge depletion and accumulation, respectively). **F** Calculated electronic density of states with the energies of FMOs marked. **G** Comparison of peroxidase-mimicking activity of M_x_V_2_O_5_·nH_2_O before and after de-intercalation. *n*  =  3 experimental replicates. Data are presented as mean values  ±  SD. Data were analyzed by using a one-sided unpaired *t*-test, **P < 0.01.

Four crystal models including V_2_O_5_, MgVO, CaVO and SrVO were established to simulate V_2_O_5_ nanobelt and three kinds of M_x_V_2_O_5_·nH_2_O nanobelts. In addition, to exclude the effect of crystal structure change (V_2_O_5_ and M_x_V_2_O_5_·nH_2_O) on the catalytic process, an additional V_2_O_5_·nH_2_O crystal model with only H_2_O intercalation was also established to study the effect of M^2+^ in catalysis comparison with M_x_V_2_O_5_·nH_2_O. At the same time, since V_2_O_5_·nH_2_O have unsaturated coordination structure relative to V_2_O_5_ like M_x_V_2_O_5_·nH_2_O, the contrast between V_2_O_5_·nH_2_O and V_2_O_5_ can be used to separately analyze the role of oxygen vacancies caused by unsaturated coordination in catalysis. The top and side views of the above five crystal models are shown in Supplementary Fig. 23. The possible catalytic mechanism of M_x_V_2_O_5_·nH_2_O catalyzing H_2_O_2_ decomposition is shown in Fig. 5A. Taking MgVO as an example, H_2_O_2_ tends to preferentially adsorb on the active site V of MgVO, and then the activated H_2_O_2_ is uniformly dissociated into two OH*. Among them, one OH* desorbs from the adsorption position to form ·OH, and the other OH* binds with a protonated hydrogen atom to form H_2_O molecule. MgVO returns to its initial state after H_2_O desorption. According to this mechanism, the free energy changes in the reaction process of the five models was shown in Fig. 5B.

Combined with the free energy profile and related literature^51,60^, it can be inferred that there are two key steps in this catalytic path, H_2_O_2_* dissociation and OH* desorption. For V_2_O_5_ before intercalation, the energy of H_2_O_2_* dissociation is 2.03 eV, indicating that the process is endothermic. For V_2_O_5_·nH_2_O after H_2_O intercalation, the energy of H_2_O_2_* dissociation decreased to 1.09 eV, indicating that the dissociation is promoted. This may be owed to the oxygen vacancies caused by unsaturated coordination in H_2_O-intercalated V_2_O_5_·nH_2_O. In spite of this, the H_2_O_2_* dissociation for V_2_O_5_·nH_2_O is still an endothermic process, and the reaction is not favorable. After Mg/Ca/Sr intercalation, the H_2_O_2_ dissociations are obviously promoted, and their free energies all become negative, that is, the process changes from endothermic to exothermic (Fig. 5B, C). Since the energy of H_2_O_2_* dissociation is determined by the difference between the adsorption energy (E_ad_) of OH* and H_2_O_2_*, E_ad_ (OH*) can be considered as descriptor of the process under the premise that E_ad_ (H_2_O_2_*) are similar. Therefore, compared with V_2_O_5_ and V_2_O_5_·nH_2_O, M^2+^ intercalation significantly enhanced the adsorption of OH*, thus promoting the dissociation of H_2_O_2_*. Further comparing the effect of different intercalation ions, CaVO showed a stronger ability than MgVO and SrVO to promote H_2_O_2_* dissociation, which is inconsistent with the order of V_max_ obtained by experiments. Considering that strong OH* adsorption is beneficial to the dissociation of H_2_O_2_*, but not conducive to the desorption of OH*, the catalytic activities may also be affected by OH* desorption. According to the free energy profile, the energy of OH* desorption on MgVO is only 0.196 eV, meaning desorption is relatively simple. But the energies of CaVO and SrVO during desorption are 0.503 eV and 0.484 eV, respectively (Fig. 5B, D), which is not favorable for the dissociation of OH*. On the whole, considering the energy from H_2_O_2_* to OH* (Step II & III), the order of MgVO (0.165 eV) < CaVO (0.216 eV) < SrVO (0.471 eV) is presented (Fig. 5B, D). It is worth noting that the energy change (Step II & III) is positively correlated with the experimental V_max_, and shows the consistency with the periodic change of M. Besides the above mechanism involving releasing ·OH, there are many reports on mechanism that the OH* oxidizes substrates directly under acidic conditions^59,60^. In this path, H_2_O_2_* first generates two OH*, and then the reductive substrates (such as TMB) continuously providing reductive hydrogen [H] to the two adsorbed OH*. Since the presence of the substrate, it is considered to be more similar in energy to the actual catalytic process. Based on this, we calculate the free energy profile under this path. As shown in Supplementary Fig. 24, the dissociation of H_2_O_2_ is not affected after the substrate is added. Compared with V_2_O_5_ and V_2_O_5_·nH_2_O, the energy of H_2_O_2_ dissociation decreases significantly after Mg/Ca/Sr intercalation. This is consistent with the result in path 1 (Fig. 5B). The reaction of OH* with TMB-H^+^ becomes completely spontaneous due to the formation of H_2_O. Therefore, from the perspective of free energy change, Ead (OH*), that is, the stability of OH* on the catalyst surface, is key to determine the catalytic reaction activity in both path 1 (Fig. 5B) and path 2 (Supplementary Fig. 24). The redox-inert M^2+^ are not considered to be catalytic centers like redox-active V. Therefore, the synergistic function of redox-inert M^2+^ through regulating the electronic structure of redox-active V may be the main way to improve their enzyme-like catalytic activities. The interlayer spacing of M_x_V_2_O_5_·nH_2_O indicated that the intercalated M^2+^ and the host was connected by non-covalent bonds^43^. Therefore, the effect of intercalated M^2+^ on the electronic structure of the crystal is mainly polarization according to their Lewis acidity, which can usually be evaluated by the ionic potential. The Ionic potential (Φ) is defined as

1 $$\Phi={{\mbox{Z}}}_{{\mbox{eff}}}/{\mbox{r}}=({\mbox{Z}}-{{{\rm{\sigma }}}})/{\mbox{r}}$$

where Z_eff_ is the effective nuclear charge, σ is the screening constant, and r is the ionic radius^63^. Among them, the effective nuclear charge is a periodic property and depends upon the electronic shell structures although it is only qualitatively conceived and measured semiempirically^64^. Since Mg^2+^, Ca^2+^ and Sr^2+^ belong to the IIA in the periodic table of the elements, they have the same ionic charge and valence shell electron configuration, so there is little difference in their Z_eff_. Therefore, the ionic potential of Mg^2+^, Ca^2+^, Sr^2+^ is inversely proportional to their ionic radius. With the increase of ionic potential, the Sr^2+^, Ca^2+^ and Mg^2+^ in the interlayer of M_x_V_2_O_5_·nH_2_O showed a periodic enhancement of polarization, thus polarizing the inherent V-O bond in the crystal and making V-OH* more stable.

To confirm the above theory, the differential charge density calculation was used to and analyze the electron transfer from M_x_V_2_O_5_·nH_2_O to OH* to determine the stability of OH*. As shown in Fig. 5E, there is basically no electron transfer (0.1181 and 0.0209 |e| respectively) between V and two OH* in V_2_O_5_ before intercalation, indicating that there is no stable interaction between V and OH*. After H_2_O intercalation (V_2_O_5_·nH_2_O), the electrons transferred between V and two OH* is significantly enhanced, reaching 0.3225 and 0.3176 |e| respectively. After M^2+^ intercalation, the number of transferred electrons increased further (0.5343/0.4325 |e | ; 0.4596/0.5301 |e | ;0.4180/0.5431 |e | ), indicating the stable bonding interaction between V and OH*. On the whole, the order of charge transfer from the V to two OH* is CaVO (0.9897 |e | ) > MgVO (0.9668 |e | ) > SrVO (0.9611 |e | ) > V_2_O_5_·nH_2_O (0.6401 |e | ) > V_2_O_5_ (0.1389 |e | ), where the charge transfers after M^2+^ intercalation are significantly greater than that of V_2_O_5_ and V_2_O_5_·nH_2_O, which corresponds to the energy of H_2_O_2_* dissociation. Then, the influence of Lewis acid M^2+^ on the intrinsic electronic structure of redox-active V is further analyzed by calculating the average bader charge of V before and after intercalation. As shown in Supplementary Fig. 25, the bader charge around V in V_2_O_5_ before intercalation is 2.1670 |e | . After H_2_O intercalation, the bader charge around V is 2.1730 |e | , and no significant change occurs. After M^2+^ intercalation, the average bader charge around V decreases to 2.1379, 2.1264 and 2.1242 |e | , respectively. Since M^2+^ acts directly on the coordinated O of V through electrostatic attraction, the polarization of M^2+^ on the V-O bond results in the decrease in electron density around V. In general, V with low electron density will be more inclined to produce stable V-OH*. Thus, the Lewis acid M^2+^ promotes the H_2_O_2_* homolysis by adjusting the electronic structure of V.

In order to further understand the effect of Lewis acid intercalation on enzyme-mimicking catalytic activity, the band structure before and after intercalation were calculated and discussed combing with the POD-like catalytic redox potential^65,66^. The typical reaction catalyzed by peroxidase nanozyme is as follows: H_2_O_2_ + 2TMB + 2H^+^ = 2H_2_O + 2TMB^+^, where TMB^+^ is the oxidation state of TMB. The reaction can be divided into two half reactions as follows:

$$\begin{array}{c}{{{{\rm{TMB}}}}}^{+}+{{{{\rm{e}}}}}^{-}={{{\rm{TMB}}}},\, {{{{\rm{\varphi }}}}}_{1}{{{\rm{;}}}}\\ {1/2{{{\rm{H}}}}}_{2}{{{{\rm{O}}}}}_{2}+{{{{\rm{H}}}}}^{+}+{{{{\rm{e}}}}}^{-}={{{{\rm{H}}}}}_{2}{{{{\rm{O}}}}}_{,} \, {{{{\rm{\varphi }}}}}_{2}\end{array}$$

The reduction potential of TMB^+^/TMB (φ_1_) used is about 1.13 V, referring to a well-established value in the literature, and the standard reduction potential of H_2_O_2_/H_2_O (φ_2_) is 1.776 V^67,68^. As can be seen from Fig. 5F, the frontier molecular orbitals (FMO) of V_2_O_5_ before intercalation, including the valence band maximum (VBM) and the conduction band minimum (CBM), display more positive energy than φ_2_, indicating that electrons can be transferred from TMB to V_2_O_5_, but cannot be transferred from V_2_O_5_ to H_2_O_2_ to complete the catalytic reaction. After Lewis acid M^2+^ intercalation, the energies of the FMO of M_x_V_2_O_5_·nH_2_O move significantly toward the negative direction, indicating that the reduction abilities of M_x_V_2_O_5_·nH_2_O are significantly enhanced. Among them, the energies of VBM is close to or less negative than φ_2_, indicating that electrons can be passed from TMB to V_2_O_5_, and then to H_2_O_2_ to complete the catalysis. The H_2_O intercalated V_2_O_5_·nH_2_O shows a more negative FMO energy, and electrons cannot transfer from TMB to V_2_O_5_.

According to the reported literatures, the redox-inert Ca^2+^ as cofactors in natural peroxidase with Lewis acidity play a key role in the catalytic performance, which is mainly manifested in the loss of Ca^2+^ leading to significant reduction of catalytic activity^16,17^. The structural similarities between M_x_V_2_O_5_·nH_2_O and natural peroxidase encouraged us to further investigate whether the redox-inert M^2+^ could also act as cofactors like Ca^2+^ of natural peroxidase to affect the peroxidase-mimicking activity of M_x_V_2_O_5_·nH_2_O. Thus, M_x_V_2_O_5_·nH_2_O were placed in heated alkaline liquid to remove the inserted M^2+^ to achieve deintercalation. The results of ICP-MS showed that the molar ratios of Mg/V, Ca/V, and Sr/V in M_x_V_2_O_5_·nH_2_O decreased to 0.08/1, 0.13/1, and 0.14/1 respectively (Supplementary Fig. 26, Supplementary Table 4), confirming partial removal of M^2+^ in M_x_V_2_O_5_·nH_2_O. The catalytic capacity of M_x_V_2_O_5_·nH_2_O for TMB oxidation before and after de-intercalation were then compared. From the result shown in Fig. 5G, the catalytic performance of M_x_V_2_O_5_·nH_2_O after de-intercalation show obvious decrement owing to the decreased concentration of M^2+^. The SEM and XRD results show that the morphology and crystal structure of M_x_V_2_O_5_·nH_2_O after partial de-intercalation have no obvious changes (Supplementary Fig. 27, 28). The above results showed that the redox-inert M^2+^ in M_x_V_2_O_5_·nH_2_O play similar roles to the Ca^2+^ in natural peroxidase in regulating the catalytic reaction. Benefiting from these, the synergism of redox-active V and redox-inert M^2+^ in M_x_V_2_O_5_·nH_2_O plays a key role in regulating the structure and function of peroxidase mimics, which is similar to the relationship between redox site and Lewis acid in natural peroxidase.

### Excellent antibacterial properties of M_x_V_2_O_5_·nH_2_O

**Fig. 6:**
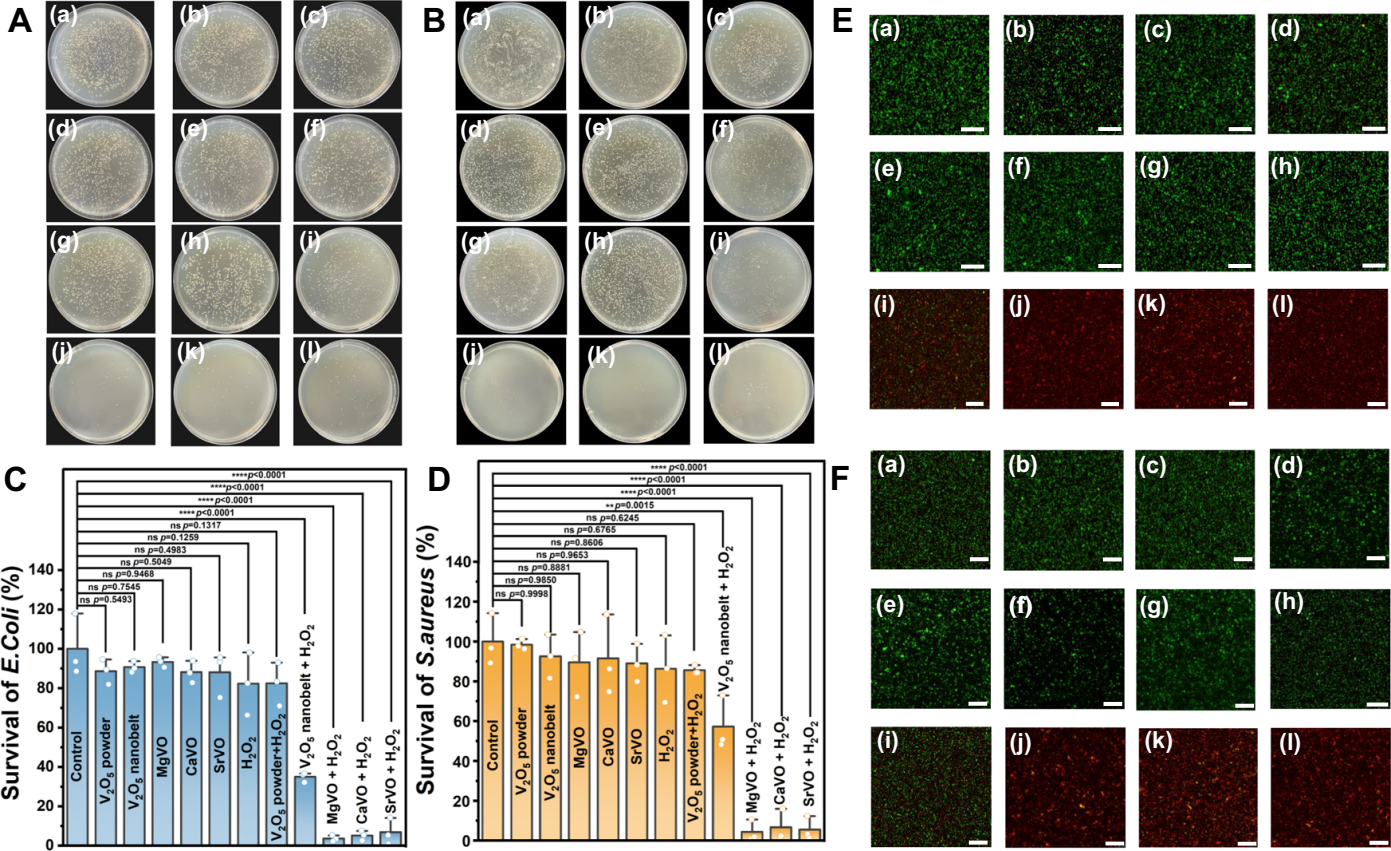
Antibacterial properties of M_x_V_2_O_5_·nH_2_O. Photographs of bacterial colonies against *E. coli* (**A**) and *S. aureus* (**B**) with different treatments. Relative survival rate of *E. coli* (**C**) and *S. aureus* (**D**) upon different treatments determined by spread plate method. *n*  =  3 biologically independent samples (**C**, **D**). Data are presented as mean values  ±  SD (**C**, **D**). Data were analyzed by one-way ANOVA with Turkey’s multiple comparisons test (**C**, **D**), ns represents no statistical difference, *P < 0.01. **P < 0.001. ***P < 0.0001. ****P < 0.0001. CLSM images of *E. coli* (**E**) and *S. aureus* (**F**) co-stained with Syto9 and PI after incubating with different treatments. Scale bar: 50 μm. (a) PBS, (b) V_2_O_5_ powder, (c) V_2_O_5_ nanobelt, (d) MgVO, (e) CaVO, (f) SrVO, (g) H_2_O_2_, (h) V_2_O_5_ powder + H_2_O_2_, (i) V_2_O_5_ nanobelt + H_2_O_2_, (j) MgVO + H_2_O_2_, (k) CaVO + H_2_O_2_, (l) SrVO + H_2_O_2_. Representative images are shown from three independent experiments with similar results (**E**, **F**).

The enzyme mimics are widely used in antibacterial researches, because the reactive oxygen species (ROS) catalyzed by enzyme mimics can oxidize key components of bacteria, such as cell membranes/walls or intracellular compartments^69–71^. In this work, M_x_V_2_O_5_·nH_2_O are expected to acquire additional bactericidal ability in the presence of low concentration of medical H_2_O_2_ disinfectant due to its high peroxidase-mimicking activity. To verify this, we evaluated the activity of M_x_V_2_O_5_·nH_2_O against gram-positive *Staphylococcus aureus* (*S. aureus*) and gram-negative *Escherichia coli* (*E. coli*) by standard spread plate method. As shown in Fig. 6A, B, in the presence of low concentration H_2_O_2_ (200 µM), M_x_V_2_O_5_·nH_2_O nanobelts show stronger antibacterial ability toward to *S. aureus* and *E. coli* than V_2_O_5_ powder and V_2_O_5_ nanobelt. Quantitative analysis shows that the relative survival rate of *E. coli* decreased to 3.63%, 5.19%, and 6.79% after M_x_V_2_O_5_·nH_2_O and H_2_O_2_ treatment, while the relative survival rate of *S. aureus* was 4.37%, 6.65%, and 5.56%, respectively (Fig. 6C, D). In control experiments without H_2_O_2_ or with H_2_O_2_ alone, the Luria Bertani (LB) agar plate were completely covered with bacterial colonies. This indicates that the bacteria could be inhibited only in the presence of H_2_O_2_ and M_x_V_2_O_5_·nH_2_O, that is, the antibacterial activity came from the peroxidase-mimicking activity of the materials. To further visually verify the antibacterial ability of the material, SYTO9/PI was used to stain live/dead bacteria. As shown in Fig. 6E, F, compared with other control groups, the red color of PI in the presence of both H_2_O_2_ and M_x_V_2_O_5_·nH_2_O was enhanced, indicating a significant antibacterial effect. To further verify the broad-spectrum antibacterial ability of M_x_V_2_O_5_·nH_2_O, the antibacterial test for gram-positive *Bacillus subtilis* (*B. subtilis*) and gram-negative *Pseudomonas aeruginosa* (*P. aeruginosa*) by standard spread plate method were added. As shown in Supplementary Fig. 29, M_x_V_2_O_5_·nH_2_O show significant antibacterial ability against *B. subtilis* and *P. aeruginosa* in the presence of low concentration H_2_O_2_.

### Composite hydrogel with M_x_V_2_O_5_·nH_2_O as additive for infected wound healing

**Fig. 7:**
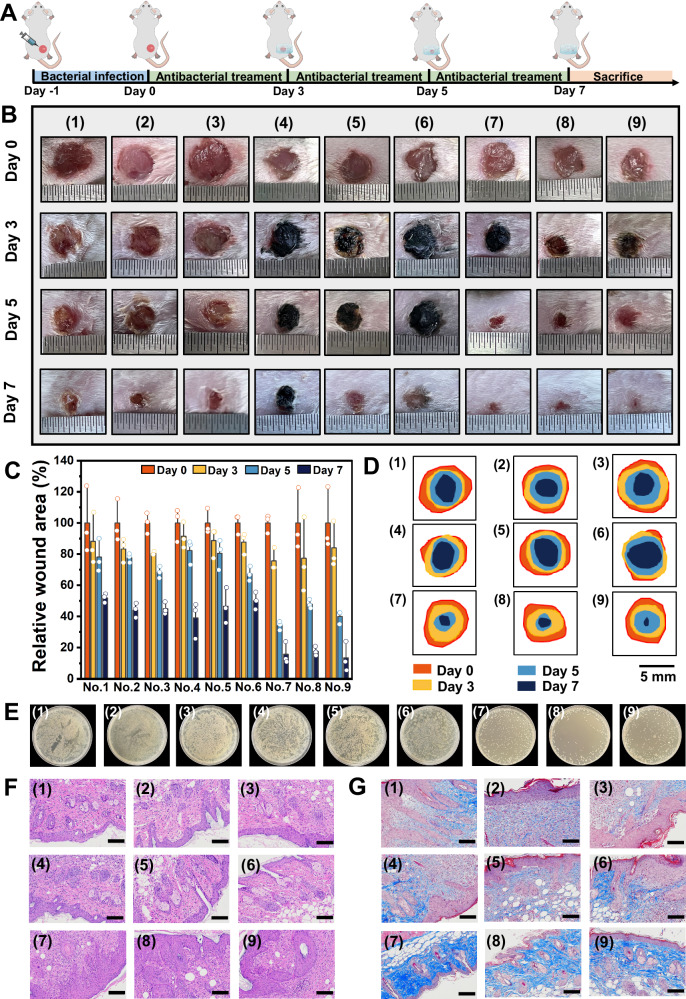
M_x_V_2_O_5_·nH_2_O functionalized hydrogels used as antibacterial dressings to promote wound healing. **A** Schematic illustration of the in vivo wound disinfection treatment with wound dressing containing M_x_V_2_O_5_·nH_2_O in a mice model. **B** Photographs of the infected wound on mice and (**C**) relative wound area of different groups at day 0, 3, 5, 7 during antibacterial treatment process. *n*  =  3 biologically independent samples. Data are presented as mean values  ±  SD. **D** Wound healing traces of mice in each group during 7 days. **E** Photographs of bacterial colonies separated from wound tissue with different treatments at day 5. **F** H&E staining and **G** Masson’s trichrome staining of the excised wound tissues treated by different groups. (1) PBS, (2) GelTA, (3) GelTA + H_2_O_2_, (4) Mg-GelTA, (5) Ca-GelTA, (6) Sr-GelTA, (7) Mg-GelTA + H_2_O_2_, (8) Ca-GelTA + H_2_O_2_, (9) Sr-GelTA + H_2_O_2_. Scale bar, 100 μm. Representative images are shown from three independent experiments with similar results (F, G).

In addition to antibacterial properties, the banded structure of M_x_V_2_O_5_·nH_2_O are widely believed to contribute to the formation of stable composites. Meanwhile, the large size reduces cytotoxicity due to the difficulty in endocytosis (Supplementary Fig. 30). Therefore, M_x_V_2_O_5_·nH_2_O nanobelts are expected to be wound dressing additives for the healing of infected wound. Hence, we then prepared an antibacterial composite hydrogel as wound dressing for wound healing. Concretely, the gelatin solution was mixed with tannic acid solution and the hydrogels (GelTA) rapidly formed via hydrogen bond crosslinking^72^. During the preparation of GelTA, M_x_V_2_O_5_·nH_2_O were added to form composite hydrogels for offering wound barrier and promoting the wound healing. Fourier transform infrared (FT-IR) spectrum was used to characterize the prepared hydrogel. The attribution of major characteristic peaks is consistent with relevant reports (Supplementary Fig. 31)^72^. SEM images showed that freeze-dried GelTA has abundant porous structure with high interconnectivity. M_x_V_2_O_5_·nH_2_O functionalized GelTA (Mg-Gel-TA/ Ca-Gel-TA/ Sr-Gel-TA) also displayed similar porous structure, which were beneficial for exchange of substances (Supplementary Fig. 32). EDX elemental quantification shows the existence of M_x_V_2_O_5_·nH_2_O in the composite hydrogel. In order to study the peroxidase-mimicking activity of three M_x_V_2_O_5_·nH_2_O functionalized GelTA, which were placed in the mixed solution of H_2_O_2_ and TMB. The results showed that the M_x_V_2_O_5_·nH_2_O functionalized GelTA could rapidly make the mixed solution turn dark blue, the absorption peak of oxTMB at 652 nm appeared in the UV-vis absorption spectrum (Supplementary Fig. 33), indicating that the M_x_V_2_O_5_·nH_2_O functionalized GelTA exhibit peroxidase-mimicking activity, which is expected to have antibacterial ability. In order to verify the antibacterial efficacy of the M_x_V_2_O_5_·nH_2_O functionalized GelTA as wound dressings, infected wound healing experiment was carried out on mice. A round whole cortical wound with a diameter of 5.5 mm was formed on the back of each mouse (Supplementary Fig. 34), which was randomly divided into 9 groups for different treatments after 24 h of *S. aureus* infection: (1) PBS, (2) GelTA, (3) GelTA+H_2_O_2_, (4) Mg-GelTA, (5) Ca-GelTA, (6) Sr-GelTA, (7) Mg-GelTA+H_2_O_2_, (8) Ca-GelTA+H_2_O_2_, (9) Sr-GelTA+H_2_O_2_. The wound photos of mice at day 0, 3, 5 and 7 were respectively shown in Fig. 7B. It could be seen that the wound had crusted and no obvious infection occurred on day 3 after treatment with Mg-GelTA+ H_2_O_2_, Ca-GelTA+ H_2_O_2_, Sr-GelTA+ H_2_O_2_. On day 5, the relative wound area of mice decreased to 34.1%, 47.8% and 39.9%, which are much smaller than other treatment groups (Fig. 7C). Besides, there were no ulceration or suppuration occurred during the treatment. In contrast, the other 6 groups show slower wound healing and varying degrees of inflammation. The wound healing traces of mice treated with different treatments within 7 days were analyzed (Fig. 7D), which further indicated that the wound healing rate of group (7) (8) (9) are significantly faster than other treatment group. In addition, the bacteria from wound tissues of mice in each group were cultured on LB agar plates, and the results showed that the number of bacteria significantly decreased at the wound sites in the treatment groups (Fig. 7E, Supplementary Fig. 35). Further, Hematoxylin and eosin (H&E) staining was analyzed for the wound tissue of different groups of mice after seven days of treatment (Fig. 7F). The results show that the epidermal layers at the wound sites are more intact in the treatment groups, and only a few inflammatory cells are distributed. In the control groups, the epidermal layers at the wound sites are fragmented and many inflammatory cells gather in the wound areas. Masson staining shows that the density of collagen fibers at the wound site are higher in the treatment groups than in the control groups (Fig. 7G). To ensure its biosafety in vivo, weight changes in mice were recorded during the treatment. As shown in Supplementary Fig. 36, there are no significant changes in body weight the body weight of mice during the treatment, indicating that the mice lived well. Further, H&E staining analysis of the major organs of the mice in all experimental groups show that no obvious organ damage, abnormality or inflammation occurred in the mice (Supplementary Fig. 37). In conclusion, M_x_V_2_O_5_·nH_2_O functionalized GelTA can effectively inhibit bacterial growth and promote wound healing as a biosafe antibacterial wound dressing.

In summary, we have developed a series of M_x_V_2_O_5_·nH_2_O peroxidase mimics with synergistic structure of redox site and Lewis acid, which have more significant peroxidase-mimicking activity compared with other typical peroxidase mimics according to the V_max_ and TON values comparison. The experimental results and theoretical calculation indicate that the prominent enzyme-mimicking catalytic activity of M_x_V_2_O_5_·nH_2_O are caused by the change of geometric and electronic structure after redox-inert M^2+^ intercalation. Specifically, the M^2+^ intercalation lead to the central redox-active V in V_2_O_5_ crystal transform into a low coordination number structure, and the resulting high concentration of oxygen vacancies can initially reduce the energy of H_2_O_2_* dissociation. More importantly, the charge redistribution around V caused by M^2+^ intercalation makes V more incline to connect with OH*, thus promoting the H_2_O_2_* dissociation to become completely spontaneous. This significantly enhanced the POD-mimicking activity of M_x_V_2_O_5_·nH_2_O. Interestingly, we found that the catalytic activity of M_x_V_2_O_5_·nH_2_O are periodically related to the Lewis acidity of intercalated M^2+^, it should be caused by the non-covalent polarization of intercalated metal ions. The redox-inert M^2+^ regulation of the geometrical and electronic structure of the V_2_O_5_ host highly mimics the synergistic effect of the redox site and Lewis acid in natural peroxidase. On this basis, M_x_V_2_O_5_·nH_2_O were applied to antibacterial therapy as an antibacterial additive in wound dressing. In vivo and in vitro experiments showed that M_x_V_2_O_5_·nH_2_O functionalized wound dressings displayed significant antibacterial activity in the presence of low concentration of medical hydrogen peroxide. This study not only reported a series of highly efficient peroxidase mimics with practical application potential, but also proposed and explained a strategy to activate enzyme-mimicking activity through synergetic structure of redox site and Lewis acid at the experimental and theoretical levels. At the same time, it also provides a general idea for the regulation of enzyme-mimicking catalytic activity of many other metal oxides layered compounds.

## Methods

### Ethical statement

All animal experiments were carried out in compliance with the 3 R principle, and were approved by Animal Ethics Committee of Shandong Normal University under approval number of AEECSDNU2022100.

### Materials

3,3’,5,5’-tetramethylbiphenamine (TMB), riboflavin and 5,5-dimethyl-1-pyrrolin-n-oxide (DMPO) were purchased from Sigma Aldrich Trading Co., Ltd. Magnesium chloride hexahydrate (MgCl_2_·6H_2_O), Calcium chloride (CaCl_2_), strontium cholride hexahydrate (SrCl_2_·6H_2_O), vanadium pentoxide (V_2_O_5_), Polyethylene glycol-4000 (PEG-4000) and dimethyl sulfoxide (DMSO) were purchased from Shanghai Macklin Biochemical Co., Ltd. Sodium acetateanhydrous, acetic acid, isopropanol, and hydrogen peroxide (wt 30%) were purchased form Sinopharm Chemical Reagent Co., Ltd. Nitro blue tetrazolium (NBT), L-Methionine, 1,2-diaminobenzene and 2,2’-azino-bis(3-ethylbenzothiazoline-6-sulfonic acid) (ABTS) were purchased from Shanghai Aladdin Biochemical Technology Co., Ltd. SYTO 9/PI Live/Dead Bacterial Double Stain Kit was purchased from Shanghai Maokang Biotechnology Co., Ltd. *E. coli* (ATCC 25922), *B. subtilis* (ATCC 6051), *S. aureus* (ATCC 6538) and *P. aeruginosa* (ATCC 27853) were purchased from Beijing Solarbio Science & Technology Co., Ltd.

### Preparation of M_x_V_2_O_5_·nH_2_O nanobelts

The M_x_V_2_O_5_·nH_2_O (M = Mg, Ca, Sr) nanobelts were synthesized by using an one-step hydrothermal method^38^. Mg_0.22_V_2_O_5_·0.75H_2_O (MgVO) was synthesized by following steps: First, 2 mmol V_2_O_5_ was dispersed to the 54 mL deionized water under stirring. After stirring for about 30 min, 10 mL H_2_O_2_ (30 wt%) was then slowly added into the above solution with vigorous stirring until the orange-red solution was obtained. Then 0.04 g PEG-4000 and 60 mmol MgCl_2_·6H_2_O was added into the solution, and stirred for 3 h at room temperature, the transparent, orange-red solution turned into a dark-red solution. The final solution is transferred into a 100 mL Teflon-lined stainless steel autoclave and hydrothermally treated at 200 °C for 48 h. The autoclave was cooled to room temperature, and the obtained products was washed with water and ethanol for three times. Finally, dark green MgVO nanobelts were obtained by vacuum dried at 60 °C for 10 h.

Ca_0.30_V_2_O_5_·0.83H_2_O (CaVO) and Sr_0.34_V_2_O_5_·0.72H_2_O (SrVO) nanobelts were also synthesized with the above method. When CaVO nanobelts were prepared, MgCl_2_·6H_2_O (60 mmol) were replaced by CaCl_2_ (60 mmol) and dispersed in 60 mL deionized water, and other conditions remain unchanged.

Likewise, when SrVO nanobelts were prepared, MgCl_2_·6H_2_O (60 mmol) were replaced by SrCl_2_·6H_2_O (60 mmol), and other conditions remain unchanged.

### Assay of peroxidase-mimicking activity of M_x_V_2_O_5_·nH_2_O nanobelts

The peroxidase-mimicking activity of M_x_V_2_O_5_·nH_2_O nanobelts were determined by measuring the oxidation of TMB in the presence of H_2_O_2_. M_x_V_2_O_5_·nH_2_O nanobelts (5 μg/mL) were added into 2 mL HAc-NaAC buffer (0.2 M, pH = 5.0) containing TMB (2 mM) and H_2_O_2_ (200 mM). The absorption intensities of oxidized TMB (oxTMB) at 652 nm were monitored by a UV-vis spectroscopy. In addition, the peroxidase-mimicking activity of M_x_V_2_O_5_·nH_2_O nanobelts was further determined by ABTS and OPD instead of TMB.

### Electrochemical detection

First, glassy carbon electrodes (GCEs) were polished using alumina oxide powder (0.05 μm) and then washed with deionized water. Then the 20 μL mixed solution containing M_x_V_2_O_5_·nH_2_O nanobelts (1 mg/mL) and Nafion (10% in alcohol) was drop on the GCE to obtain the M_x_V_2_O_5_·nH_2_O modified GCEs. Cyclic voltammetry (CV) were carried out to monitor the catalytic activity of the M_x_V_2_O_5_·nH_2_O modified GCEs in 3 M KCl with different concentrations of H_2_O_2_ (0.1–0.5 mM) and fixed concentrations of TMB (0.2 mM).

### Steady-state kinetic assays

The Steady-state kinetic assays were carried out in a 2.0 mL cuvette (optical path *l*: 5 mm) containing M_x_V_2_O_5_·nH_2_O nanobelts (5 μg/mL) in 2 mL HAc-NaAc buffer (0.2 M, pH = 5.0). To obtained kinetic data toward for TMB, the different concentrations of TMB (0.2-3 mM) and the fixed concentrations of H_2_O_2_ (200 mM) were added to the 2 mL HAc-NaAc buffer (0.2 M, pH = 5.0) containing M_x_V_2_O_5_·nH_2_O nanobelts (5 μg/mL). Similarly, to obtained kinetic data toward for H_2_O_2_, the different concentrations of H_2_O_2_ (2–300 mM) and the fixed concentrations of TMB (2 mM) were added to the 2 mL HAc-NaAc buffer (0.2 M, pH = 5.0) containing M_x_V_2_O_5_·nH_2_O nanobelts (5 μg/mL). The absorbance intensities at 652 nm was measured by a UV spectroscopy as a function of time with time intervals of 1 s for 1 min. The initial reaction rate within 5 s was calculated according to the following formula: ${{{\rm{v}}}}={\Delta}{\mbox{A}}/{\Delta}{\mbox{t}}\left({\varepsilon}_{652}\times l\right)$, where ε_652_ is the molar absorption coefficient of oxidized TMB at 652 nm (ε_652_ = 3.9×10^4^ M^−^^1^·cm^−^^1^), and *l* is the optical path of cuvette (*l* = 5 mm). The kinetic parameter was derived from the Michaelis-Menten equation: ${\mbox{v}}=({{\mbox{V}}}_{\max }\times [{\mbox{S}}])/\left({{\mbox{K}}}_{{\mbox{m}}}+[{\mbox{S}}]\right)$, where v is the initial velocity, [S] is the concentration of the substrate, K_m_ is the Michaelis−Menten constant, and V_max_ is the maximal reaction velocity. ${\mbox{TON}}={{\mbox{V}}}_{\max }/[{\mbox{E}}]$, where [E] is the molar concentration of MgVO/CaVO/SrVO.

### DFT calculation

The spin-polarized density functional theory (DFT) calculations have been conducted on Vienna ab-initio simulation package (VASP)^73,74^ to study the alkaline hydrogen evolution reaction. The Projector augmented wave method^75^ with a cutoff energy of 450 eV accompanied by Perdew-Burke-Ernzerhof functional^75^ has been used in the DFT calculations. DFT + U method^76^ with the effective U value of 3.0 eV^77^ and DFT-D3 method^78^ was used to correct the influence of 3d electrons of V atom and van der Waals interactions, respectively. Two layers of V_2_O_5_ (001) facet have been cleaved with a vacuum layer of 15 Å to build the V_2_O_5_ slab model and half of bottom layers have been fixed to simulate the bulk phase. Four of Mg-atom, Ca-atom and Sr-atom with 16 of H_2_O molecules have been inserted in the interlamination of V_2_O_5_, respectively to build the MgVO, CaVO and SrVO models. Besides, 16 of H_2_O molecules have been inserted in the interlamination of V_2_O_5_ to build the V_2_O_5_·nH_2_O model. All models have been fully relaxed with the energy convergence criterion of 10^−5^ eV and the force convergence criterion of 0.02 eV/Å, respectively. The free energy of H^+^ is obtained by H^+^ + e^-^→0.5 H_2_ according to the National Institute of Standards and Technology chemistry webbook and the free energy of TMB-H^+^ has been calculated by regarding TMB-H^+^ as a radical. The (1 × 2 × 1) K-points have been used as K-point mesh. The adsorption energy (E_ads_) has been calculated using formula 2,

2 $${{\mbox{E}}}_{{\mbox{ads}}}={{\mbox{E}}}_{{\mbox{total}}}-{{\mbox{E}}}_{{\mbox{substrate}}}-{{\mbox{E}}}_{{\mbox{adsorbate}}}$$

The E_total_, E_substrate_ and E_adsorbate_ represent the energy of adsorption structure, substrate and adsorbate, respectively. The free energies have been calculated using the following formula 3,

3 $${\mbox{G}}={{\mbox{E}}}_{{\mbox{DFT}}}+{\mbox{ZPE}}-{\mbox{TS}}$$

The G, E_DFT_, ZPE and TS represent the free energy, energy from DFT calculations, zero point energy and entropic contributions, respectively.

### The deintercalation on the activity of M_x_V_2_O_5_·nH_2_O

MgVO, CaVO and SrVO were respectively placed in deionized water and the solution is adjusted to pH = 10.0 with 1 M NaOH. The above solution was heated under nitrogen for 8 h. Then the products were collected and alternately cleaned with ethanol and water for three times, finally, the MgVO, CaVO and SrVO after deintercalation were obtained by vacuum dried at 60 °C for 10 h. The peroxidase-mimicking activity of M_x_V_2_O_5_·nH_2_O before and after deintercalation under the same condition with 2 mL HAc-NaAC buffer (0.2 M, pH = 5.0) containing TMB (2 mM) and H_2_O_2_ (200 mM).

### In vitro antibacterial activity

The antibacterial activity of nanozymes were evaluated by a spread plate method. In brief, *E. coli* (ATCC 25922) or *S. aureus* (ATCC 6538) were respectively diluted with sterilized HAc-NaAc buffer (0.2 mM, pH = 5.0) to 10^5^ CFU·mL^−^^1^ and added to 24-well cell culture plates. Then V_2_O_5_ powder, V_2_O_5_ nanobelt, M_x_V_2_O_5_ (MgVO/CaVO/SrVO) with final concentration of 100 µg/mL and H_2_O_2_ (200 µM) were added, respectively. The total volume of solution in each well was 500 μL. After incubation at 37 °C and 180 rpm for 30 min, 100 μL bacterial suspension was taken out and evenly coated on LB agar plate. The survival bacterial colonies were present on the plates by culturing at 37 °C for 12 h.

### Live/dead fluorescent staining of bacteria

A confocal laser microscope was used to observe the survival and death status of bacteria. The bacterial suspension (*E. coli* (ATCC 25922) or *S. aureus* (ATCC 6538), 10^5^ CFU mL^−^^1^) was respectively diluted by sterilized HAc-NaAc buffer (0.2 mM, pH = 5.0), then the bacterial suspension was incubated with 100 µg/mL V_2_O_5_ powder, V_2_O_5_ nanobelt, M_x_V_2_O_5_·nH_2_O(MgVO/CaVO/SrVO) and 200 µM H_2_O_2_ at 37 °C at 180 rpm for 4 h. Finally, the bacteria were stained using the SYTO 9/PI live/dead bacterial double stain kit.

### Cytotoxicity validation of M_x_V_2_O_5_·nH_2_O

The effect of M_x_V_2_O_5_·nH_2_O on cell viability was determined using CCK8 (Beyotime Biotech, China). HMEC-1 (CRL-3243) and L929 (CCL-1) cells were acquired commercially form ATCC, which were authenticated by STR. Briefly, HMEC-1or L929 cells were digested into suspension and plated in 96-well plates with 5000 cells per well for 24 h incubation. Then, these cells were cocultured with various concentrations (20-300 μg/mL) of M_x_V_2_O_5_·nH_2_O for 8 h. After the cells were washed, 100 μL of CCK8 dilution was added, and the cells were incubated for some time to detect the absorbance of cells at 450 nm.

### Preparation of GelTA hydrogel

GelTA hydrogels were prepared by hydrogen bonding between gelatin and tannic acid (TA) using the previously reported method^72^. Gelatin is dissolved by stirring in deionized water at 70 °C to obtain 10% w/v gelatin solution. Then, 0.5 mg/mL TA solution was prepared in deionized water. 120 μL TA solution is added drop by drop to 1 mL 10% w/v gelatin solution and stirred to obtain GelTA hydrogel. Finally, the GelTA hydrogel was freeze-dried for later using.

### Preparation of wound dressing based on M_x_V_2_O_5_·nH_2_O

100 mg gelatin were added to 0.9 mL deionized water and heat it to dissolve at 70 °C, then 500 μL 1 mg·mL^−^^1^ MgVO/CaVO/SrVO were respectively added to the gelatin solution, finally, 120 μL 0.5 mg·mL^−^^1^ TA solution was added to the mixed solution, and the M_x_V_2_O_5_·nH_2_O functionalized GelTA hydrogels were obtained by stirring the solution as wound dressing. After being freeze-dried, a round wound dressing with uniform size (5.5 mm diameter) was obtained by a hole punch.

### In vivo Wound healing assessment with *S. aureus* infection

All animal experiments were approved by the Animal Ethics Committee of Shandong Normal University (AEECSDNU2022100). We established a full-thickness wound model caused by *S. aureus* (ATCC 6538) on the back of female Balb/c mice (6 weeks, wide-type, Jinan Pengyue Laboratory Animal Breeding Co., LTD., China). This study only included female mice, and we did not pay attention to the effect of gender on wound healing. Since male mice are more prone to bite wounds and dressings than female mice when fed in the same cage, we chose more docile female mice. All mice fed in a barrier environment and mice were kept in constant temperature (22°C), constant humidity (55%) and cyclic lighting (12 h light/12 h dark). Prior to surgery, mice were anesthetized with intraperitoneal injection of 100 μL chloral hydrate (3.5%). After removing the back hair of mice, the 5.5 mm diameter full-thickness wounds were established with surgical scissors. 50 μL *S. aureus* (ATCC 6538) suspension (1 × 10^8^ CFU mL^−1^) were added to the wound sites, after 24 h infection, mice were randomly divided into nine groups and different treatment were administered to the wound sites of the nine groups and each group had an average of three mice: (1) PBS, (2) GelTA, (3) GelTA+H_2_O_2_, (4) Mg-GelTA, (5) Ca-GelTA, (6) Sr-GelTA, (7) Mg-GelTA+H_2_O_2_, (8) Ca-GelTA+H_2_O_2_, (9) Sr-GelTA+H_2_O_2_. The concentration of H_2_O_2_ was the same as in vitro antibacterial test. The wound sites of mice were treated every 24 h, different wound dressings were fixed on the wound sites with medical gauze, and the wound dressings were removed after 1 h. The body weight of mice was recorded, and the wound area was measured and photographed to record the wound healing process at day 0, 3, 5 and 7, respectively. The wound dressings were changed when the wound areas were being photographed.

The number of bacteria at the wound sites were measured by spread plate method, mice with different treatment were sacrificed on day 7 and the related wound tissues were harvested and placed in normal saline. The obtained bacterial suspension was spread on LB agar plates and incubated at 37 °C for 18 h.

### Histological and immunofluorescence analysis

After 7 days of treatment, mice (female Balb/c, 6 weeks, wide-type) in each group were sacrificed, and the related wound tissue was fixed in 4% paraformaldehyde for 24 h. Subsequently, wound tissues were embedded in paraffin, sectioned, stained with hematoxylin and eosin (H&E) staining and Masson’s trichrome staining to evaluate the wound healing effect. All samples were examined with a microscope.

### Reporting summary

Further information on research design is available in the Nature Portfolio Reporting Summary linked to this article.

### Supplementary information

Supplementary Information

Reporting summary

Supplementary Data 1

Description of Additional Supplementary Files

Peer Review File

### Source data

Source data

## Data Availability

All data generated in this study are provided in the Supplementary Information/Source Data file. Source data are provided with this paper.
