# Supplementary material for: Biomimetic synergistic effect of redox site and Lewis acid for construction of efficient artificial enzyme
Source: Nat Commun. 2024 Jul 26;15:6315. doi: 10.1038/s41467-024-50687-1 (PMC11282276; doi:10.1038/s41467-024-50687-1)
Supplement: Supplementary file 1 — Supplementary Information [file 41467_2024_50687_MOESM1_ESM.docx]

**Supplementary information**

**Biomimetic synergistic effect of redox site and Lewis acid for construction of efficient artificial enzyme**

Haibin Si^1†^, Dexin Du^1†^, Chengcheng Jiao^1^, Yan Sun^1^, Lu Li*^1, 2^, Bo Tang*^1, 3^

^1^College of Chemistry, Chemical Engineering and Materials Science, Collaborative Innovation Center of Functionalized Probes for Chemical Imaging, Key Laboratory of Molecular and Nano Probes, Ministry of Education, Shandong Normal University, Jinan 250014, P. R. China.

^2^Jinan Institute of Quantum Technology, Jinan 250101, P. R. China.

^3^Laoshan Laboratory, Qingdao 266237, P. R. China.

^†^These authors contributed equally: Haibin Si, Dexin Du.

*E-mail: lilu5252@163.com, tangb@sdnu.edu.cn.

Table of contents

[1. Supplementary Methods 1](#_Toc171501562)

[**1.1 Characterization** 1](#_Toc171501563)

[**1.2 Assay of Catalase-mimicking activity of M_x_V_2_O_5_·nH_2_O nanobelts** 1](#_Toc171501564)

[**1.3 Assay of Superoxide Dismutase (SOD)-mimicking activity of M_x_V_2_O_5_·nH_2_O nanobelts** 1](#_Toc171501565)

[**1.4 Assay** **of Oxidase-mimicking Activity of M_x_V_2_O_5_·nH_2_O nanobelts** 1](#_Toc171501566)

[**1.5 Assay** **of glutathione peroxidase-mimicking activity of M_x_V_2_O_5_·nH_2_O nanobelts** 2](#_Toc171501567)

[**1.6 Assay** **of haloperoxidase-mimicking activity of M_x_V_2_O_5_·nH_2_O nanobelts** 2](#_Toc171501568)

[**1.7 Measurement of ·OH** 2](#_Toc171501569)

[**1.8 Bacterial culture** 2](#_Toc171501570)

[**1.9 In vivo toxicity analysis** 2](#_Toc171501571)

[2. Supplementary Figures and Tables 3](#_Toc171501572)

[**Supplementary Fig. 1** **Characterization of V_2_O_5_ powder and V_2_O_5_ nanobelt.** 3](#_Toc171501573)

[**Supplementary Fig. 2** **TEM images.** 3](#_Toc171501574)

[**Supplementary Fig. 3** **Width profiling of M_x_V_2_O_5_·nH_2_O by SEM images.** 3](#_Toc171501575)

[**Supplementary Fig. 4** **Height profiling of M_x_V_2_O_5_·nH_2_O by AFM (atomic force microscope).**. 4](#_Toc171501576)

[**Supplementary Fig. 5 Thermogravimetric analysis.** 4](#_Toc171501577)

[**Supplementary Fig. 6 XRD pattern.** . 5](#_Toc171501578)

[**Supplementary Fig. 7** **XPS spectra.** 5](#_Toc171501579)

[**Supplementary Fig. 8 Optimization of reaction conditons.** 6](#_Toc171501580)

[**Supplementary Fig. 9 Stability analysis of M_x_V_2_O_5_·nH_2_O**. 6](#_Toc171501581)

[**Supplementary Fig. 10 Stability analysis of M_x_V_2_O_5_·nH_2_O**. 6](#_Toc171501582)

[**Supplementary Fig. 11 Assay of peroxidase-mimicking activity.** 7](#_Toc171501583)

[**Supplementary Fig. 12 Electrochemical analysis of peroxidase-mimicking activity.** 7](#_Toc171501584)

[**Supplementary Fig. 13 Electrochemical stability analysis.** 8](#_Toc171501585)

[**Supplementary Fig. 14 Assay of oxidase/ catalase/ superoxide dismutase-mimicking activity.** 8](#_Toc171501586)

[**Supplementary Fig. 15 Assay of glutathione peroxidase-mimicking activity.** 8](#_Toc171501587)

[**Supplementary Fig. 16 Assay of haloperoxidase-mimicking activity.** 9](#_Toc171501588)

[**Supplementary Fig. 17 Steady-state kinetics of CaVO.** 9](#_Toc171501589)

[**Supplementary Fig. 18** **Steady-state kinetics of SrVO.** 10](#_Toc171501590)

[**Supplementary Fig. 19** **Steady-state kinetics of** **V_2_O_5_ nanobelt.** 10](#_Toc171501591)

[**Supplementary Fig. 20 XPS spectra.** 11](#_Toc171501592)

[**Supplementary Fig. 21 TEM images.** 11](#_Toc171501593)

[**Supplementary Fig. 22 XRD patterns**. 11](#_Toc171501594)

[**Supplementary Fig. 23 Crystal models.** 12](#_Toc171501595)

[**Supplementary Fig. 24 DFT calculation of reaction pathway.** 12](#_Toc171501596)

[**Supplementary Fig. 25 The bader charge and differential charge analysis.** 12](#_Toc171501597)

[**Supplementary Fig. 26 The molar ratios of Mg/V, Ca/V, and Sr/V in M_x_V_2_O_5_·nH_2_O before and after deintercalation.** 13](#_Toc171501598)

[**Supplementary Fig. 27** **SEM images.** 13](#_Toc171501599)

[**Supplementary Fig. 28 XRD patterns.** 13](#_Toc171501601)

[**Supplementary Fig. 29 Antibacterial properties of M_x_V_2_O_5_·nH_2_O.** 14](#_Toc171501602)

[**Supplementary Fig. 30 Cytotoxicity validation of M_x_V_2_O_5_·nH_2_O.** 14](#_Toc171501603)

[**Supplementary Fig. 31 FTIR spectra of hydrogel wound dressing.** 15](#_Toc171501604)

[**Supplementary Fig. 32 SEM images with EDX elemental quantification of functionalized wound dressing.** 15](#_Toc171501605)

[**Supplementary Fig. 33 Assay of peroxidase-mimicking activity of functionalized wound dressing.** 16](#_Toc171501606)

[**Supplementary Fig. 34 The diameter of wound at day 0 measured by vernier caliper.**](#_Toc171501607)

[**Supplementary Fig. 35 Count of bacteria number.** 17](#_Toc171501608)

[**Supplementary Fig. 36. Weight analysis of mice.** 17](#_Toc171501609)

[**Supplementary Fig. 37 Histological analysis.** 18](#_Toc171501610)

[**Supplementary Table 1** 19](#_Toc171501611)

[**Supplementary Table 2** 20](#_Toc171501612)

[**Supplementary Table 3** 21](#_Toc171501613)

[**Supplementary Table 4** 22](#_Toc171501614)

**1.** **Supplementary Methods**

**1.1 Characterization**

Thermo Fisher Talors F2OOS G2(TEM, HRTEM, EDS-mapping), Hitacihi Cold Field Emisson Scanning Electron Microscope (SEM), RigaKu Ultima IV X-ray diffractormeter (XRD), Escalab 250Xi (XPS), HORIBA LabRAM HR Evolution Raman Spectrometer (Raman), Therm Fisher Scientific IS50 FI-IR Spectormeter (FT-IR), Bruker EMX plus-6/1 (ESR), Bruker Dimension Icon (AFM), Shimadzu UV-2600i (UV-Vis), Thermo scientific XSeries2 (ICP-MS), Mettler Toledo TGA/DSC 3+ (TGA), Leica STELLARIS 5 confocal laser scanning microscop (CLSM).

**1.2 Assay of Catalase-mimicking activity of** **M_x_V_2_O_5_·nH_2_O nanobelts**

The catalase- mimicking activity of M_x_V_2_O_5_·nH_2_O nanobelts was determined by measuring the O_2_ produced during the decomposition of H_2_O_2_ by a portable dissolved oxygen meter. 5 μg/mL M_x_V_2_O_5_·nH_2_O nanobelts were added to HAc-NaAc buffer (0.2 M, pH = 5.0) containing 10 mM H_2_O_2_ and the generated O_2_ solubility (OD, mg/L) produced within 5 min was recorded.

**1.3 Assay of Superoxide Dismutase (SOD)-mimicking activity of M_x_V_2_O_5_·nH_2_O nanobelts**

The SOD-mimicking activity of M_x_V_2_O_5_·nH_2_O nanobelts was determined by measuring inhibition of the photoreduction of nitro blue tetrazolium (NBT). Riboflavin (20 μM), methionine (13 μM), NBT (75 μM), M_x_V_2_O_5_·nH_2_O (5 μg/mL) were mixed in HAc-NaAc buffer (0.2 M, pH = 5.0). The mixed solution was illuminated by a lamp with a constant light intensity for 10 min at room temperature. After illumination, immediately the absorbance at 560 nm was measured. The whole reaction was enclosed in a box lined with aluminum foil. The tubs containing the same reaction solution were placed in the dark as a blank group. SOD-mimicking activity was calculated according to the following formula: SOD-mimicking activity (%) = [(A_0_-A)/A_0_] ×100%, where A_0_ is the absorbance of the control group at 560 nm, and A is the absorbance of the sample.

**1.4 Assay** **of Oxidase-mimicking Activity of M_x_V_2_O_5_·nH_2_O nanobelts**

The Oxidase- mimicking activity of M_x_V_2_O_5_·nH_2_O nanobelts were determined by measuring the oxidation of TMB, which is indicated by the absorbance at 652 nm. In typical assay, M_x_V_2_O_5_·nH_2_O nanobelts (5 μg/mL) were added into 2 mL HAc-NaAC buffer (0.2 M, pH = 5.0) containing TMB (2 mM), and the change of absorbance of 652 nm was recorded. In addition, the Oxidase-mimicking activity of M_x_V_2_O_5_·nH_2_O nanobelts was further determined by ABTS and OPD instead of TMB.

**1.5 Assay** **of glutathione peroxidase-mimicking activity of M_x_V_2_O_5_·nH_2_O nanobelts**

M_x_V_2_O_5_·nH_2_O nanobelts (5 μg/mL) were added into 2 mL phosphate buffer (pH 7.4) containing 2 mM GSH, 0.4 mM H_2_O_2_, and 0.4 mM NADPH at room temperature. The absorbances at 432 and 590 nm were recorded.

**1.6 Assay** **of haloperoxidase-mimicking activity of M_x_V_2_O_5_·nH_2_O nanobelts**

M_x_V_2_O_5_·nH_2_O nanobelts (5 μg/mL) were added into 2 mL NaAc/HAc buffer (0.2 M, pH = 5.0) containing 28 μM phenol red, 4.4 mM NH_4_Br, and 0.42 mM H_2_O_2_ at room temperature. The absorbances at 340 nm were recorded.

**1.7 Measurement of ·OH**

Electron paramagnetic resonance (ESR) spectroscopy was applied to determine the generation of ·OH with 5, 5-dimethyl-1-pyrroline N-oxide (DMPO) as spin trap. 40 μL DMPO (100 mM), 20 μL H_2_O_2_ (5 mM), 1 μg M_x_V_2_O_5_·nH_2_O were added into a black centrifugal tube containing HAc-NaAc buffer (0.2 M, pH = 5.0), After mixing, 2 μL mixed solution was transferred into a quartz capillary immediately and determined by ESR.

Isopropanol was also used to detect the generation of ·OH. In brief, Isopropanol (100 mM) was added to 2 mL HAc-NaAC buffer solution (0.2 M, pH = 5.0) containing TMB (2 mM), H_2_O_2_ (200 mM) and M_x_V_2_O_5_·nH_2_O nanobelts (5 μg/mL). Then the absorption intensities of oxidized TMB at 652 nm were record.

**1.8 Bacterial culture**

*E. coli* (ATCC 25922)/*B. subtilis* (ATCC 6051), and *S. aureus* (ATCC 6538)/*P. aeruginosa* (ATCC 27853) were used as Gram-negative and Gram-positive models, respectively. The monocolony of *E. coli*, *B. subtilis, P. aeruginosa* or *S. aureus* were cultured in Luria-Bertani (LB) liquid medium (neutral pH) at constant 37 °C under 180 rpm for 12 h. The concentration of bacteria was quantified by measuring the optical density of medium using a microplate reader. (Colony forming units, CFU·mL^-1^). In all experiments, the bacteria were allowed to grow to a logarithmic stage (OD_600_ = 0.6-0.8) before using.

**1.9 In vivo toxicity analysis**

After 7 days of treatment, the mice (female Balb/c, 6 weeks, wide-type) were sacrificed, and the major organs (heart, liver, spleen, lung and kidney) were collected and fixed with 4% paraformaldehyde. Finally, all organ samples were subjected to H&E staining. All samples were examined with a microscope.

**2. Supplementary Figures and Tables**


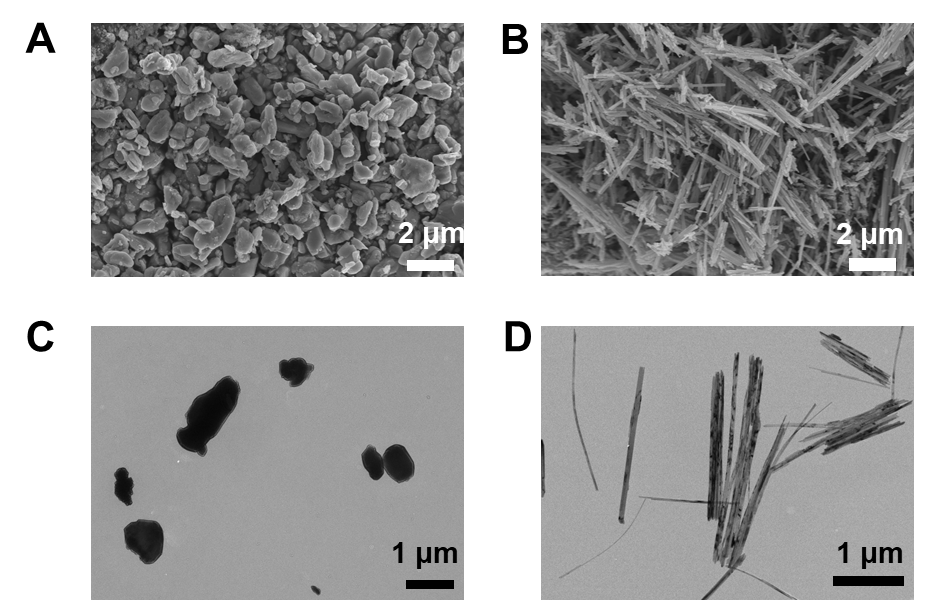


**Supplementary Fig. 1** **Characterization of V_2_O_5_ powder and V_2_O_5_ nanobelt.** SEM images of (A) V_2_O_5_ powder and (B) V_2_O_5_ nanobelt. TEM images of (C) V_2_O_5_ powder and (D) V_2_O_5_ nanobelt. Representative images are shown from three independent experiments with similar results.


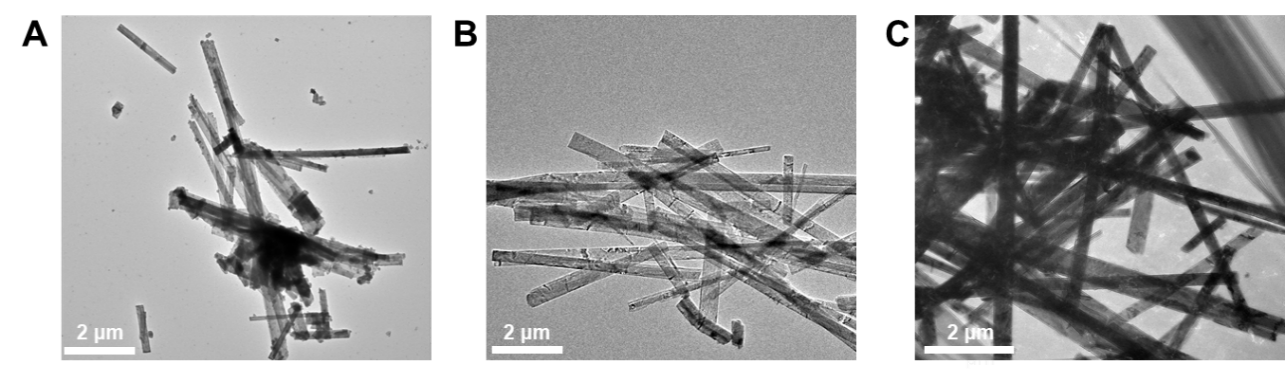


**Supplementary Fig. 2** **TEM images.** The TEM images with a large field of view of MgVO (A), CaVO (B) and SrVO (C). Representative images are shown from three independent experiments with similar results.


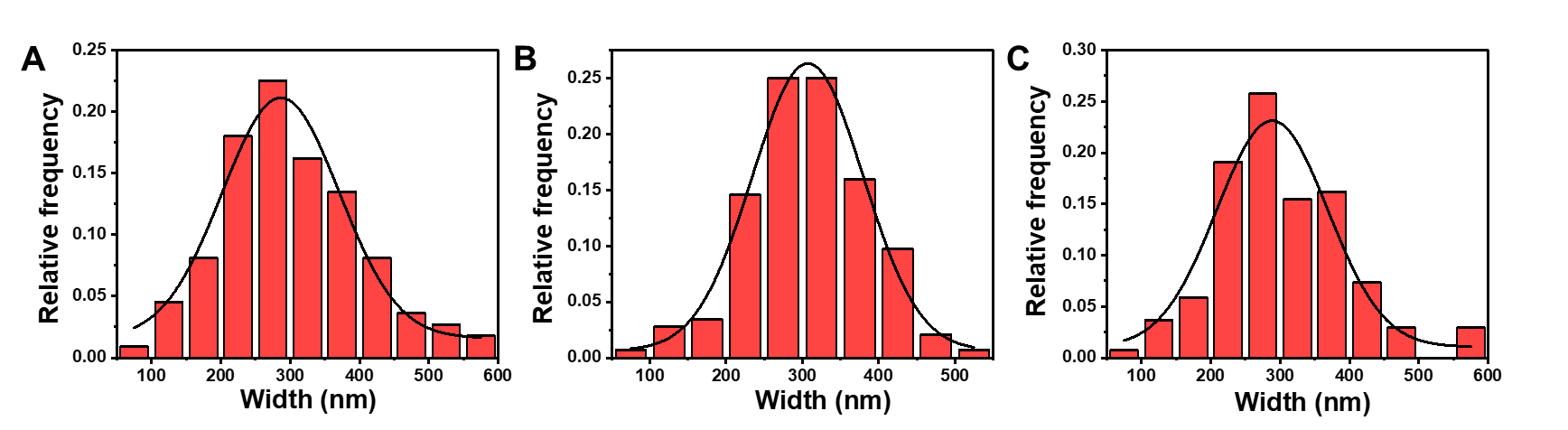


**Supplementary Fig. 3** **Width profiling of M_x_V_2_O_5_·nH_2_O by SEM images.** The width distributions of MgVO (A), CaVO (B) and SrVO (C).


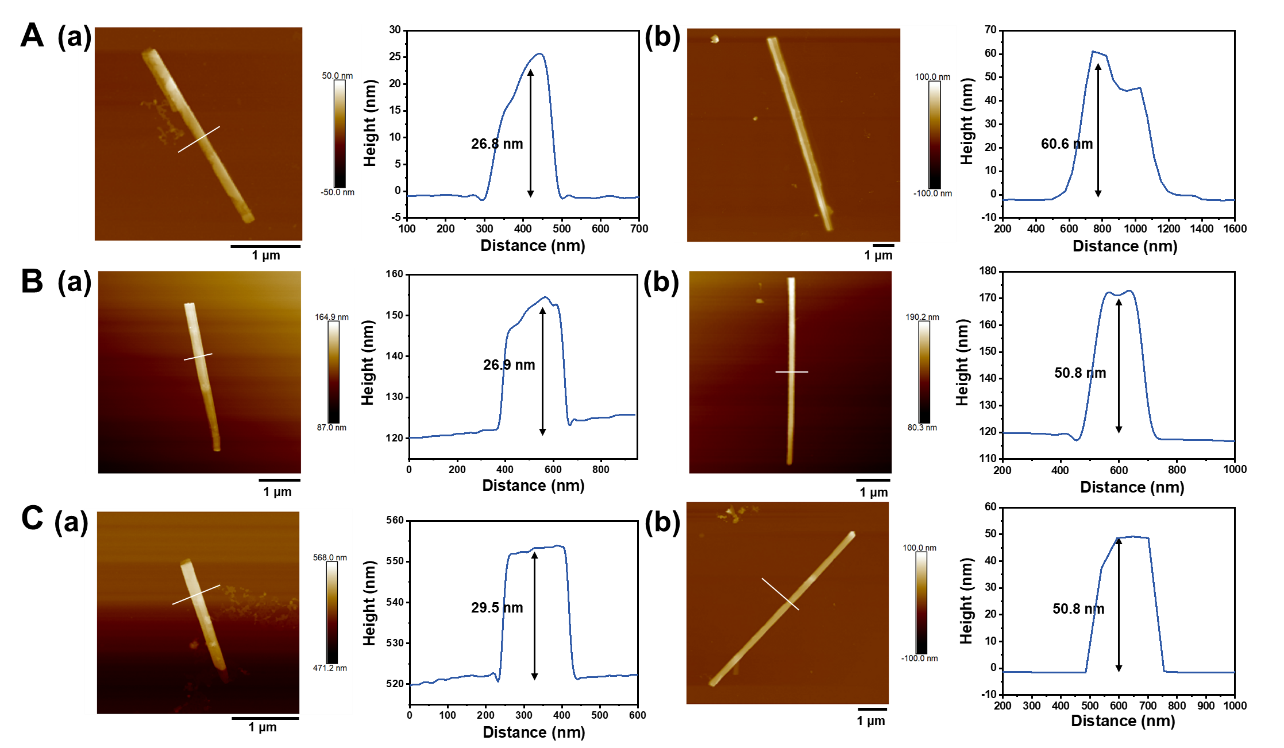


**Supplementary Fig. 4** **Height profiling of M_x_V_2_O_5_·nH_2_O by AFM (atomic force microscope).** AFM images (left) with height profile (right) of MgVO (A), CaVO (B) and SrVO (C). (a), (b) are two representative materials, respectively.


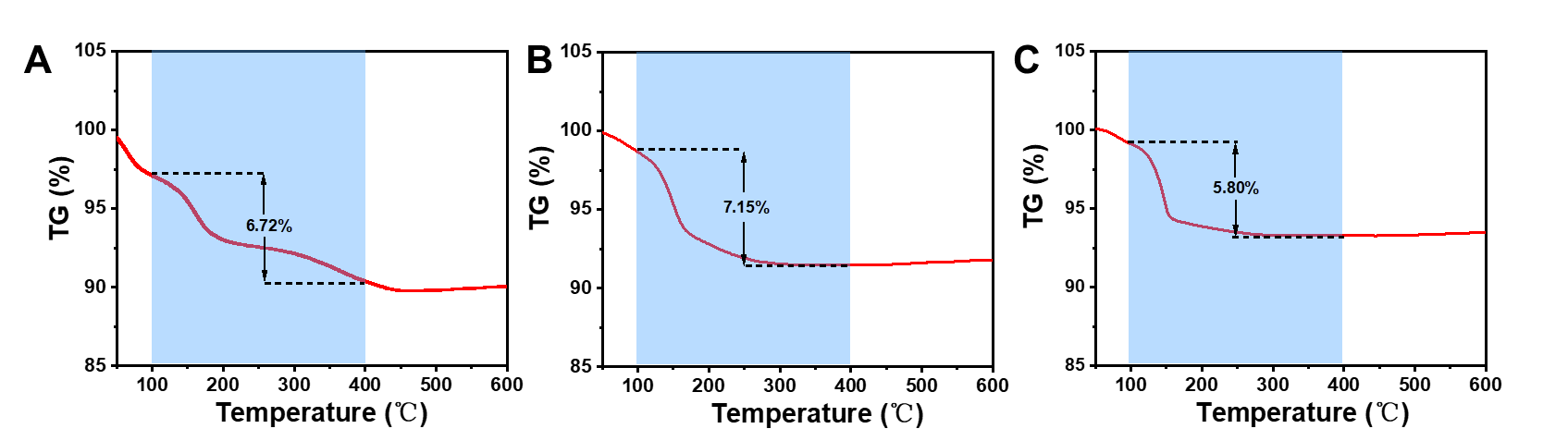


**Supplementary Fig. 5 Thermogravimetric analysis.** (A) MgVO, (B) CaVO and (C) SrVO.


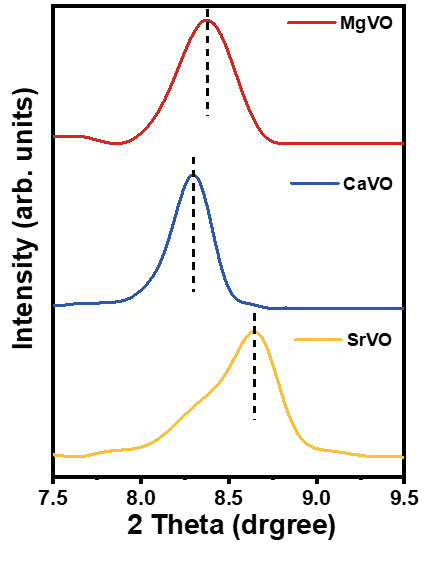


**Supplementary Fig. 6 XRD pattern.** The (001) peak after amplification of MgVO, CaVO and SrVO.


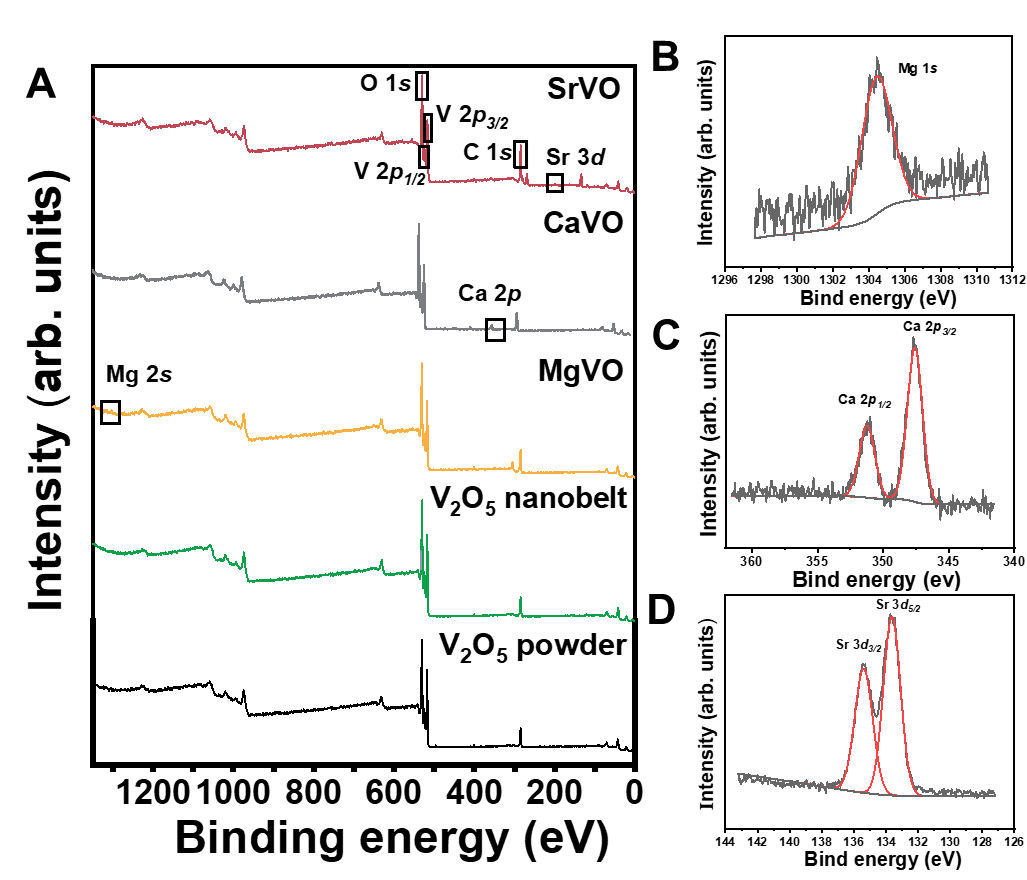


**Supplementary Fig. 7** **XPS spectra.** (A) XPS spectra of M_x_V_2_O_5_·nH_2_O, V_2_O_5_ powder, V_2_O_5_ nanobelt. (B) XPS high-resolution Mg 1*s* spectrum of MgVO. (C) XPS high-resolution Ca 2*p* spectrum of CaVO. (D) XPS high-resolution Sr 3*d* spectrum of SrVO.

**
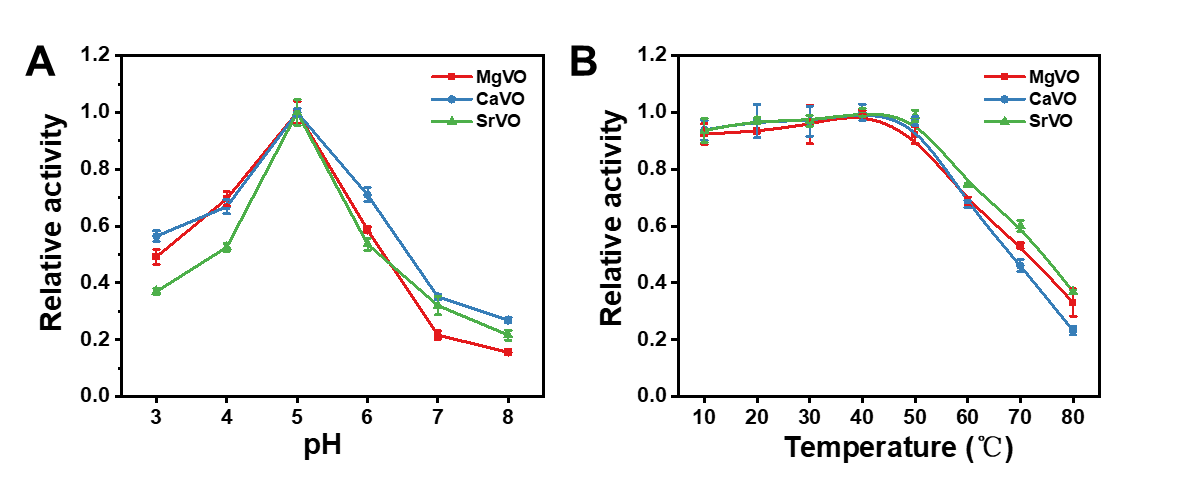
**

**Supplementary Fig. 8 Optimization of reaction conditons.** Changes in peroxidase-mimicking activity of MgVO, CaVO, and SrVO at different (A) pH and (B) temperatures**.** Data are presented as mean values ± SD. n = 3 experimental replicates.

**
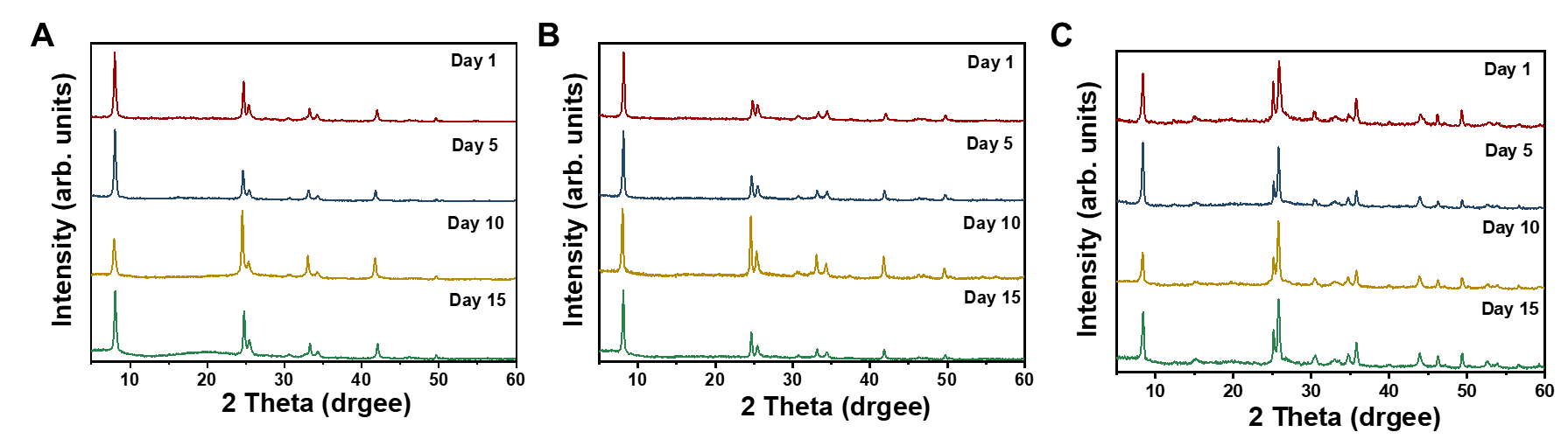
**

**Supplementary Fig. 9 Stability analysis of M_x_V_2_O_5_·nH_2_O**. The XRD patterns of MgVO (A), CaVO (B) and SrVO (C) under HAc-NaAC buffer (0.2 M, pH = 5.0) within 15 days.

**
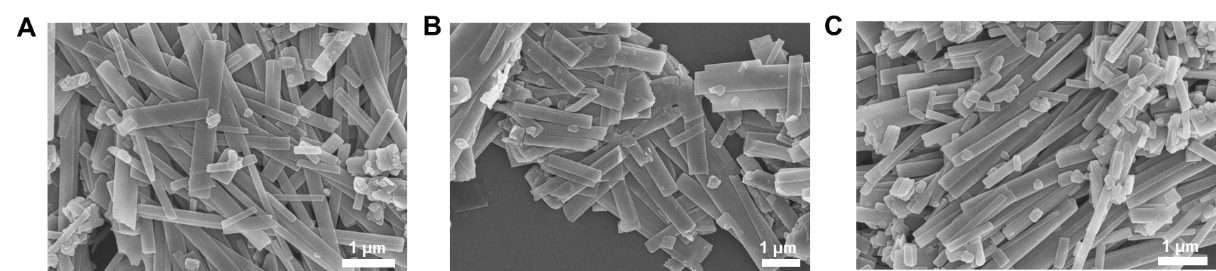
**

**Supplementary Fig. 10 Stability analysis of M_x_V_2_O_5_·nH_2_O**. The SEM images of MgVO (A), CaVO (B) and SrVO (C) under HAc-NaAC buffer (0.2 M, pH = 5.0) within 15 days. Representative images are shown from three independent experiments with similar results.


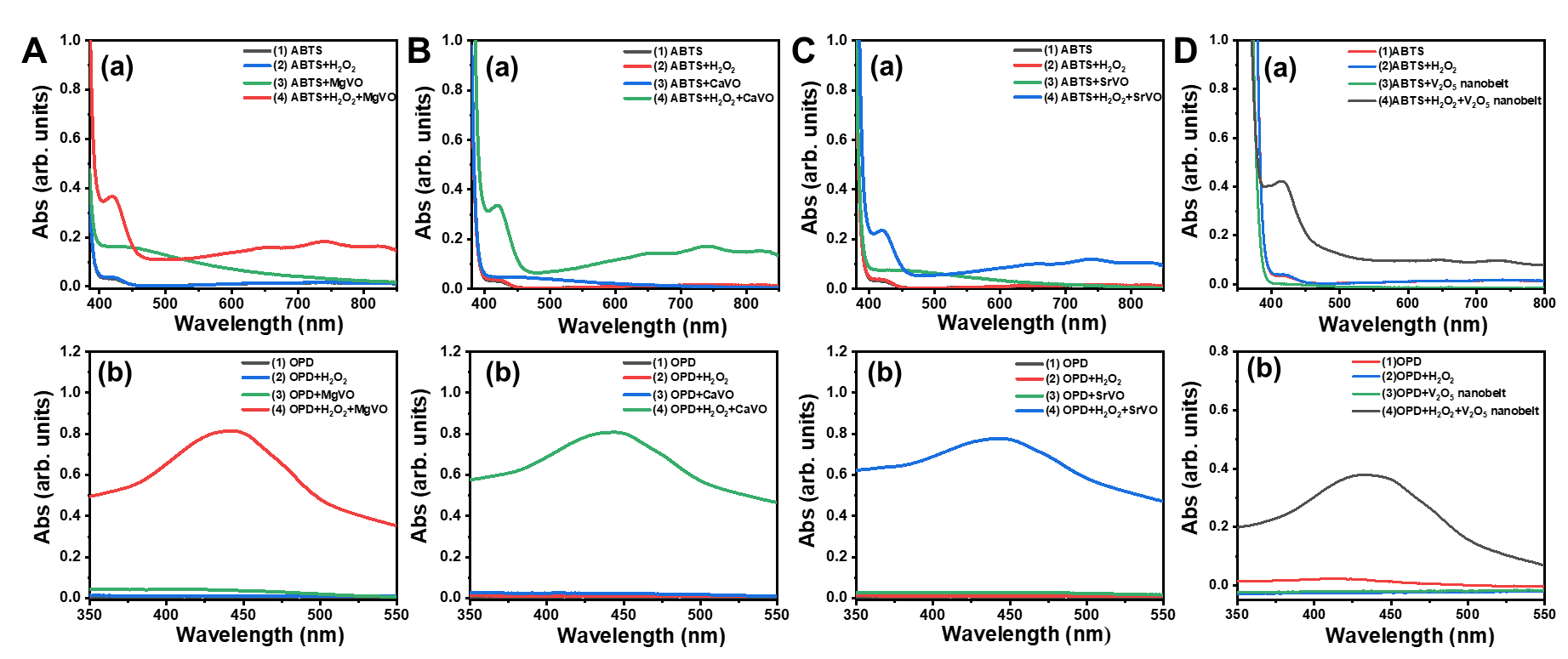


**Supplementary Fig. 11 Assay of peroxidase-mimicking activity.** Exploration of peroxidase-mimicking activity of (A) MgVO, (B) CaVO, (C) SrVO and (D) V_2_O_5_ nanobelt. UV-vis absorption spectra of different reaction systems at (a) 450 nm when ATBS as substrate and 420 nm (b) when OPD as substrate.


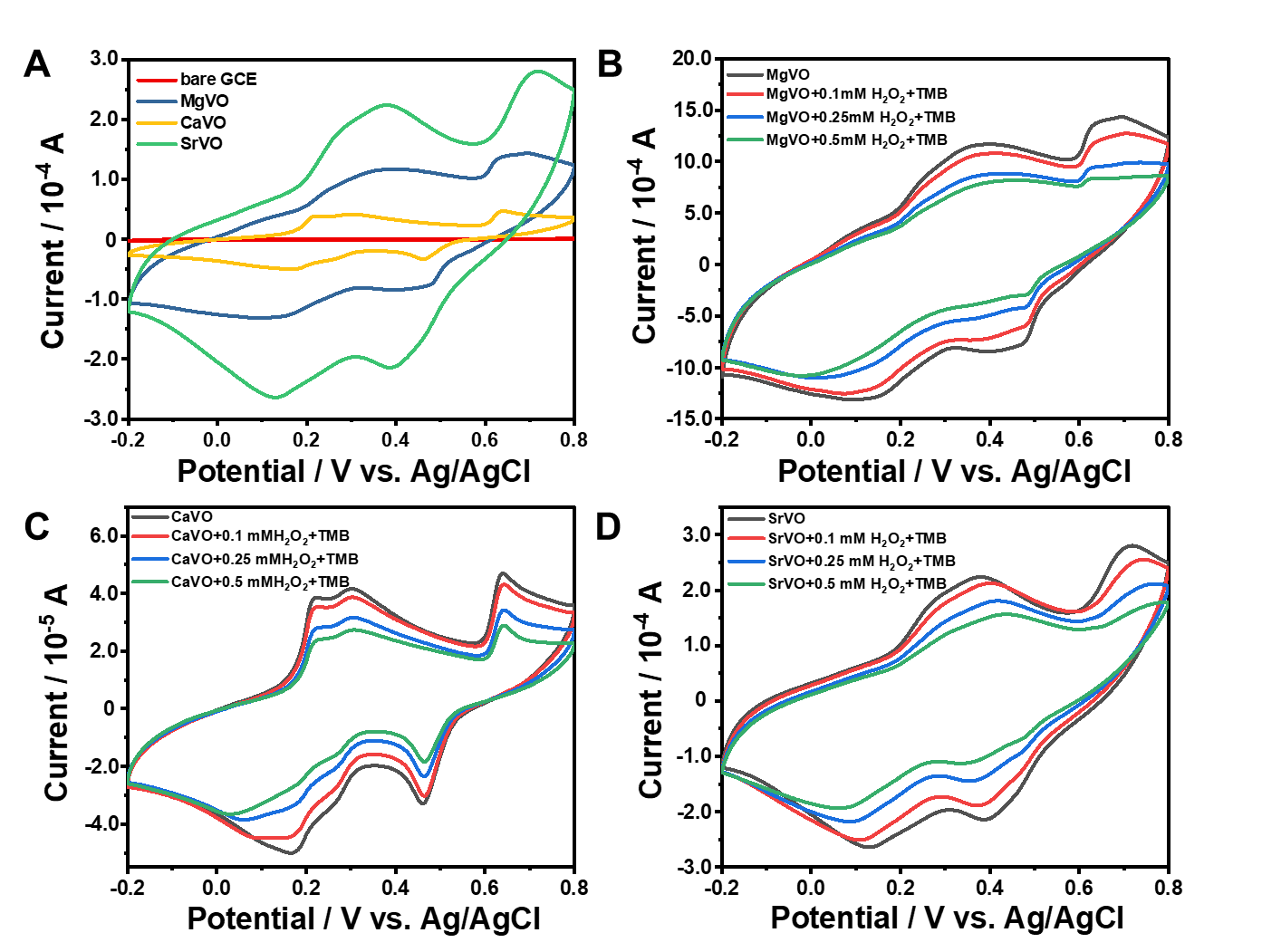


**Supplementary Fig. 12 Electrochemical analysis of peroxidase-mimicking activity.** (A) The CV curvers of bare GCE and M_x_V_2_O_5_·nH_2_O modified GCE in 3 M KCl, scan rate: 50 mV/s. The CV curvers of MgVO (B), CaVO (C) and SrVO (D) modified GCE in absence or presence of different concentrations of H_2_O_2_ (0.1- 0.5 mM) and fixed concentrations of TMB (0.2 mM), scan rate: 50 mV/s.


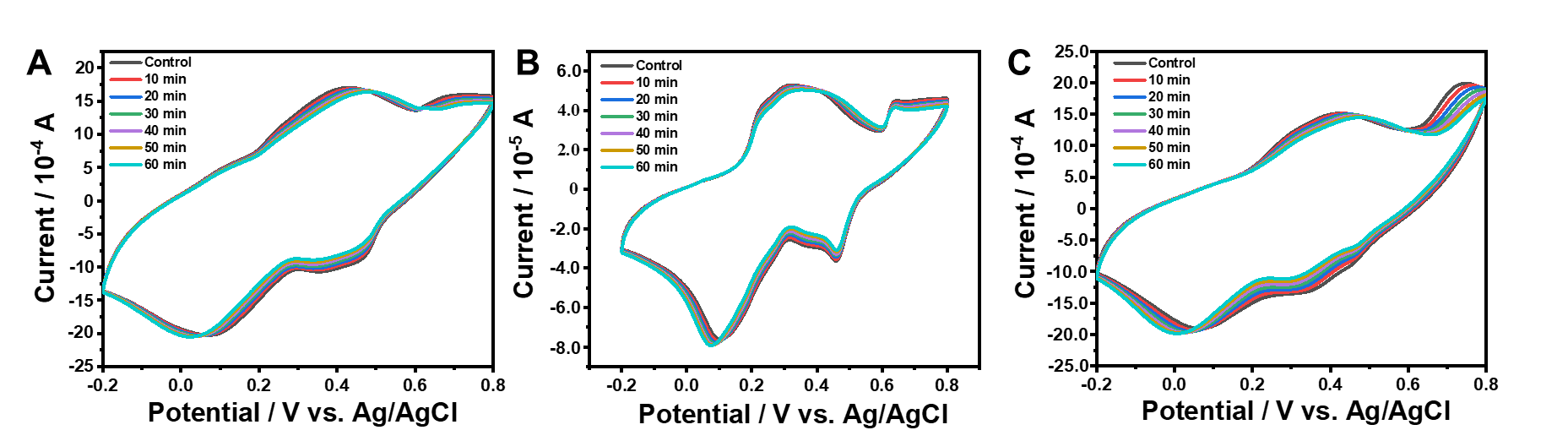


**Supplementary Fig. 13 Electrochemical stability analysis.** Time-dependent CV curvers of MgVO (A), CaVO (B) and SrVO (C) modified GCE with different time in 3 M KCl (scan rate: 50 mV/s) showed that the CV curve does not change significantly, demonstrating the electrochemical stability of the M_x_V_2_O_5_·nH_2_O.


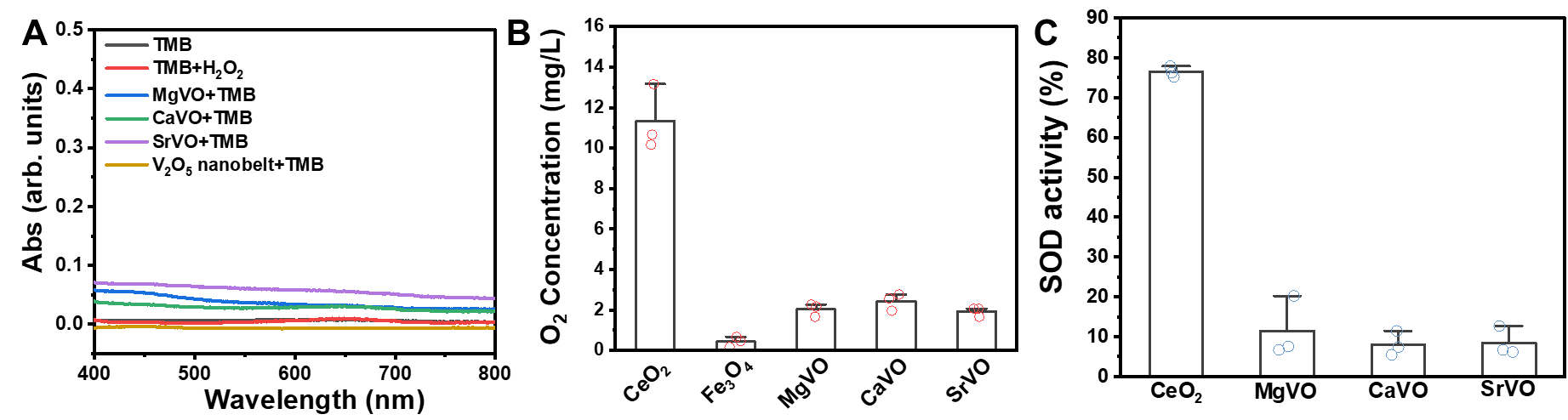


**Supplementary Fig. 14 Assay of oxidase/ catalase/ superoxide dismutase-mimicking activity.** Exploration of oxidase-mimicking activity (A) and catalase-mimicking activity (B) and superoxide dismutase- mimicking activity (C) of M_x_V_2_O_5_·nH_2_O. n = 3 experimental replicates (B, C). Data are presented as mean values ± SD (B, C).

**
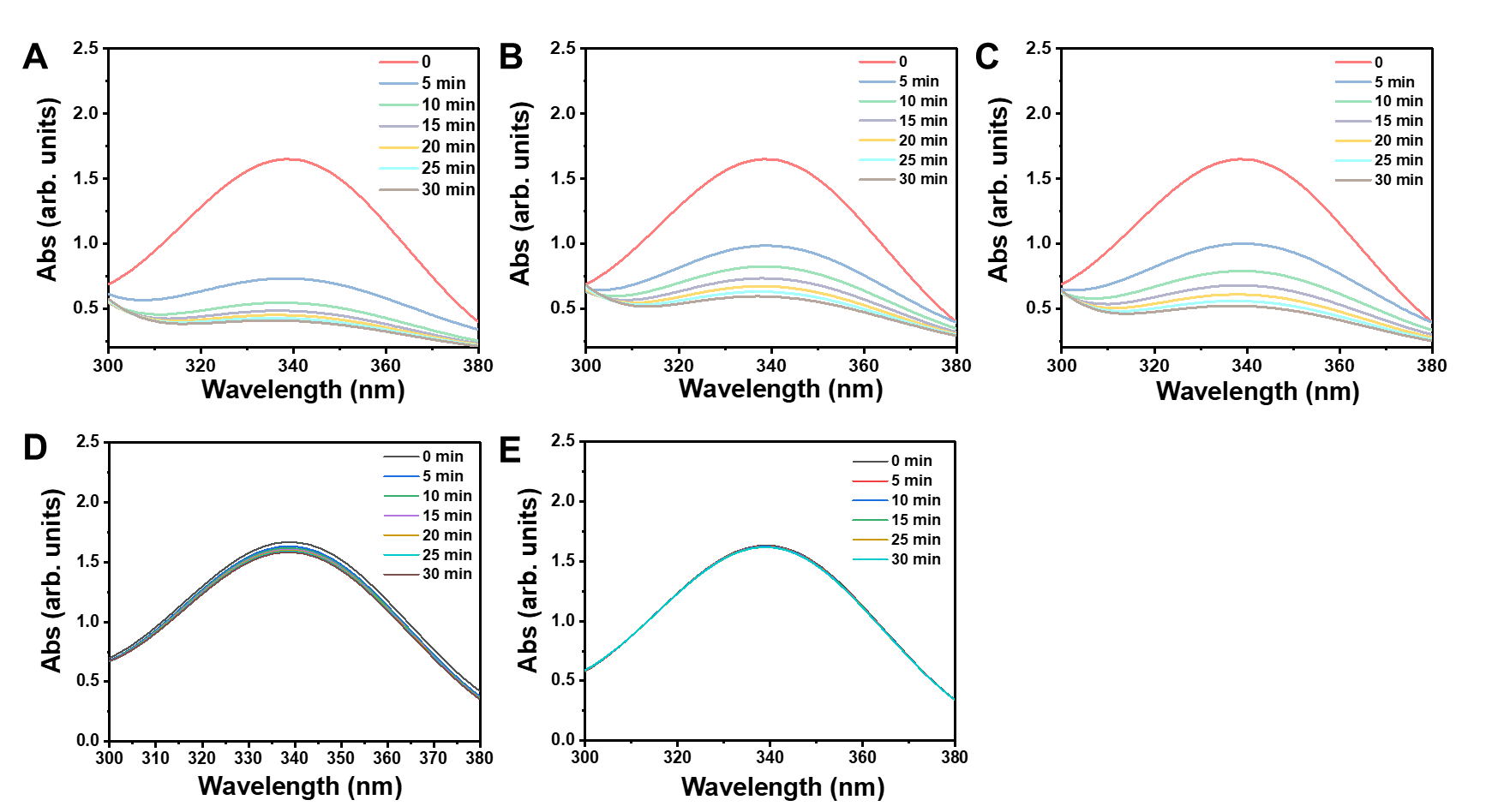
**

**Supplementary Fig. 15 Assay of glutathione peroxidase-mimicking activity.** Exploration of glutathione peroxidase-mimicking activity of M_x_V_2_O_5_·nH_2_O. Time-dependent UV-vis absorption spectra for monitoring the glutathione peroxidase-mimicking catalytic activities of MgVO (A), CaVO (B) and SrVO (C), under the condition of phosphate buffer (pH 7.4) containing 2 mM GSH, 0.4 mM H_2_O_2_, and 0.4 mM NADPH at room temperature. Time-dependent UV-vis absorption spectra for monitoring the glutathione peroxidase-mimicking catalytic activities of M_x_V_2_O_5_·nH_2_O, under the above condition without GSH (D) or without H_2_O_2_ (E).

**
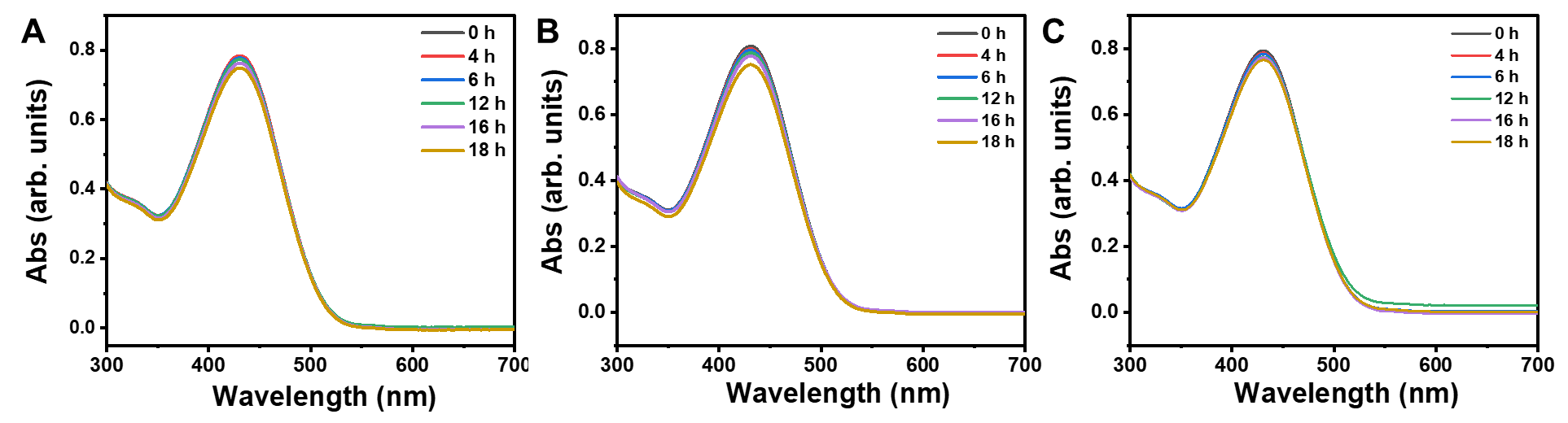
**

**Supplementary Fig. 16 Assay of haloperoxidase-mimicking activity.** Exploration of haloperoxidase-mimicking activity of M_x_V_2_O_5_·nH_2_O. Time-dependent UV-vis absorption spectra for monitoring the glutathione peroxidase-mimicking catalytic activities of MgVO (A), CaVO (B) and SrVO (C), under the condition of NaAc/HAc buffer (pH = 5.0) containing 28 μM phenol red, 4.4 mM NH_4_Br, and 0.42 mM H_2_O_2_ at room temperature.

**
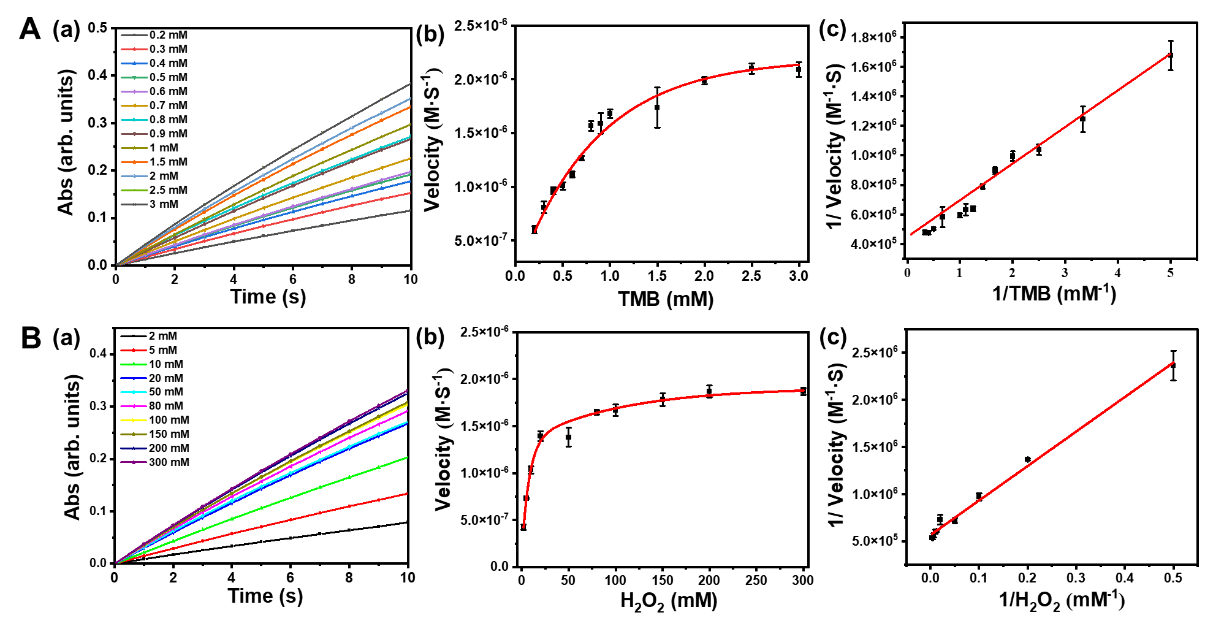
**

**Supplementary Fig. 17 Steady-state kinetics of CaVO.** (A) Time-dependent absorbance of oxTMB (a), reaction rate (b) with different concentrations of TMB and fixed concentrations of CaVO/H_2_O_2_, and the corresponding double reciprocal (Lineweaver-Burk) plots (c). (B) Time-dependent absorbance of oxTMB (a), reaction rate (b) with different concentrations of H_2_O_2_ and fixed concentrations of CaVO/TMB, and the corresponding double reciprocal (Lineweaver-Burk) plots (c). Data are presented as mean values ± SD. n = 3 experimental replicates.

**
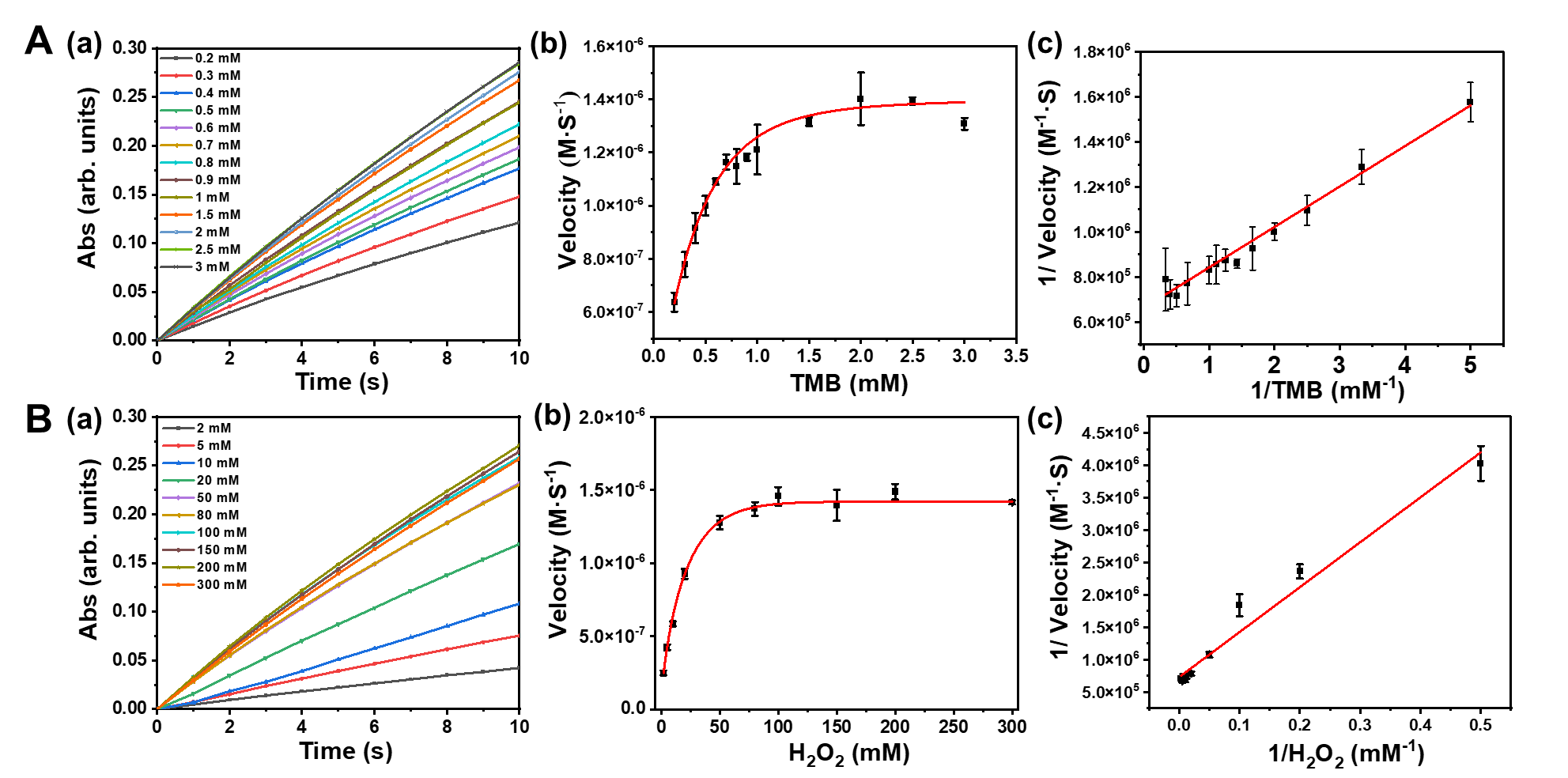
**

**Supplementary Fig. 18** **Steady-state kinetics of SrVO.** (A) Time-dependent absorbance of oxTMB (a), reaction rate (b) with different concentrations of TMB and fixed concentrations of SrVO/H_2_O_2_, and the corresponding double reciprocal (Lineweaver-Burk) plots (c). (B) Time-dependent absorbance of oxTMB (a), reaction rate (b) with different concentrations of H_2_O_2_ and fixed concentrations of SrVO/TMB, and the corresponding double reciprocal (Lineweaver-Burk) plots (c). Data are presented as mean values ± SD. n = 3 experimental replicates.

**
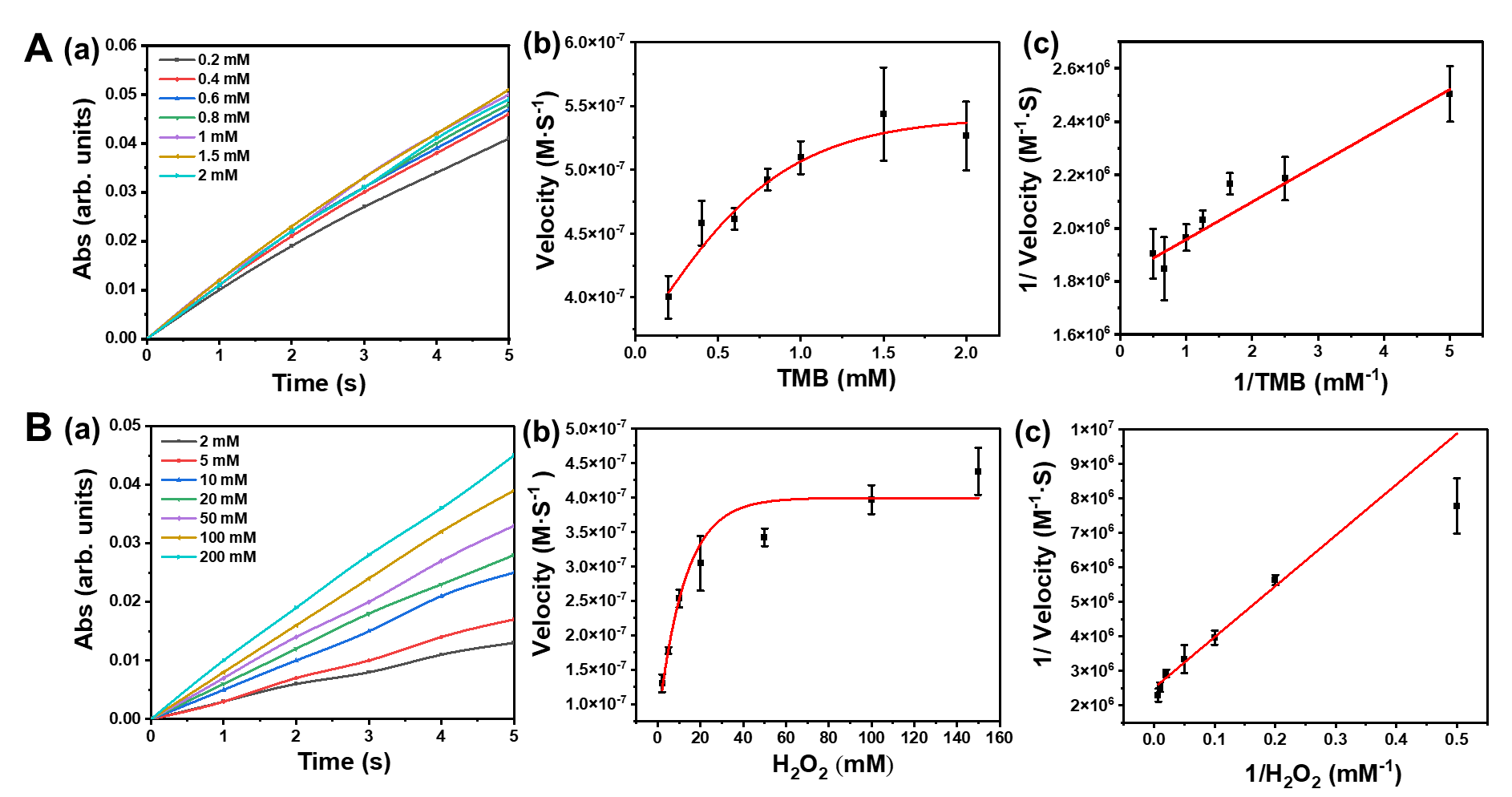
**

**Supplementary Fig. 19** **Steady-state kinetics of** **V_2_O_5_ nanobelt.** (A) Time-dependent absorbance of oxTMB (a), reaction rate (b) with different concentrations of TMB and fixed concentrations of V_2_O_5_ nanobelt/H_2_O_2_, and the corresponding double reciprocal (Lineweaver-Burk) plots (c). (B) Time-dependent absorbance of oxTMB (a), reaction rate (b) with different concentrations of H_2_O_2_ and fixed concentrations of V_2_O_5_ nanobelt/TMB, and the corresponding double reciprocal (Lineweaver-Burk) plots (c). Data are presented as mean values ± SD. n = 3 experimental replicates.

**
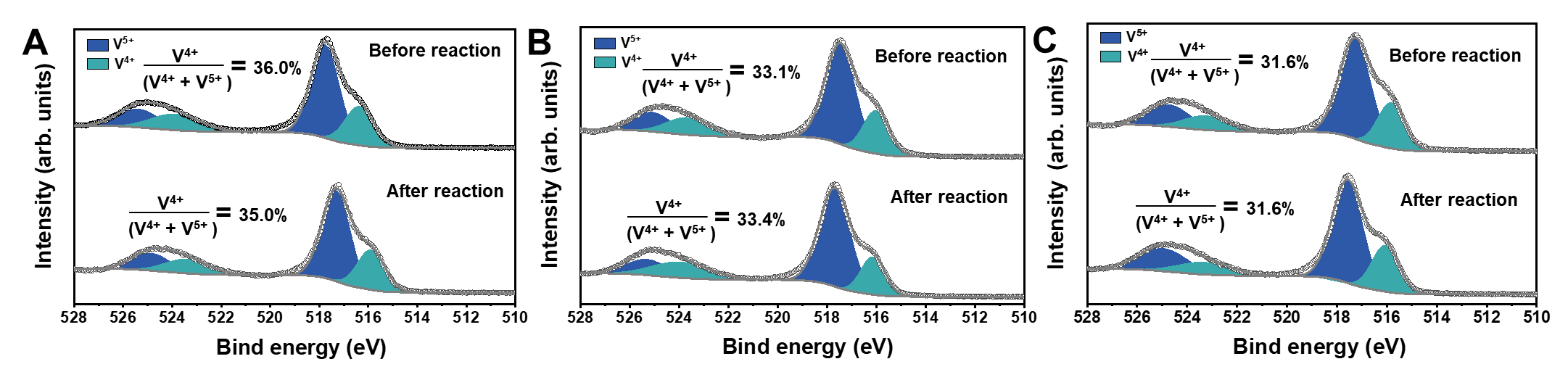
**

**Supplementary Fig. 20 XPS spectra.** V 2*p* of MgVO (A), CaVO (B), and SrVO (C) before and after enzymatic reaction.

**
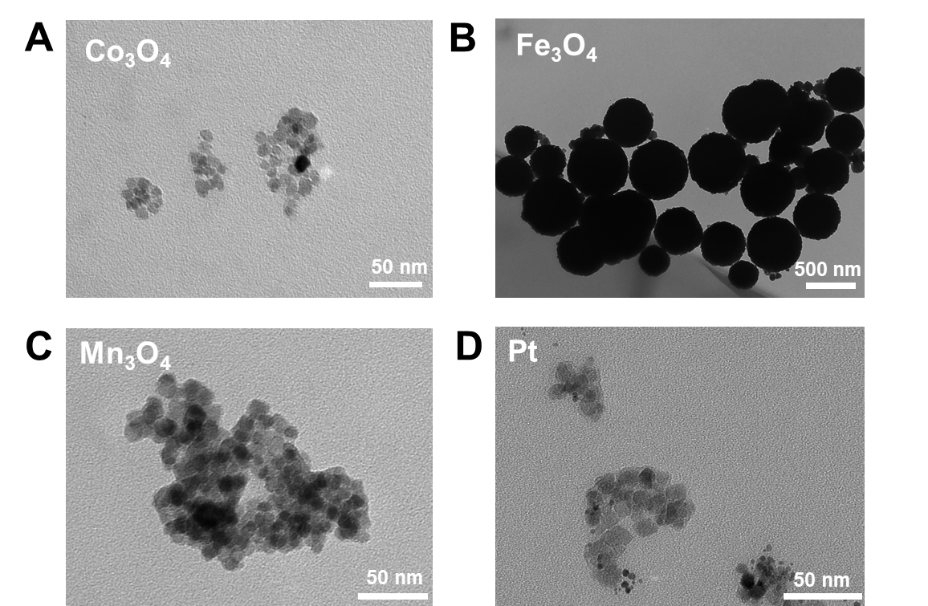
**

**Supplementary Fig. 21 TEM images****.** (A) Co_3_O_4_, (B) Fe_3_O_4_, (C) Mn_3_O_4_, (D) Pt NPs. Representative images are shown from three independent experiments with similar results.

**
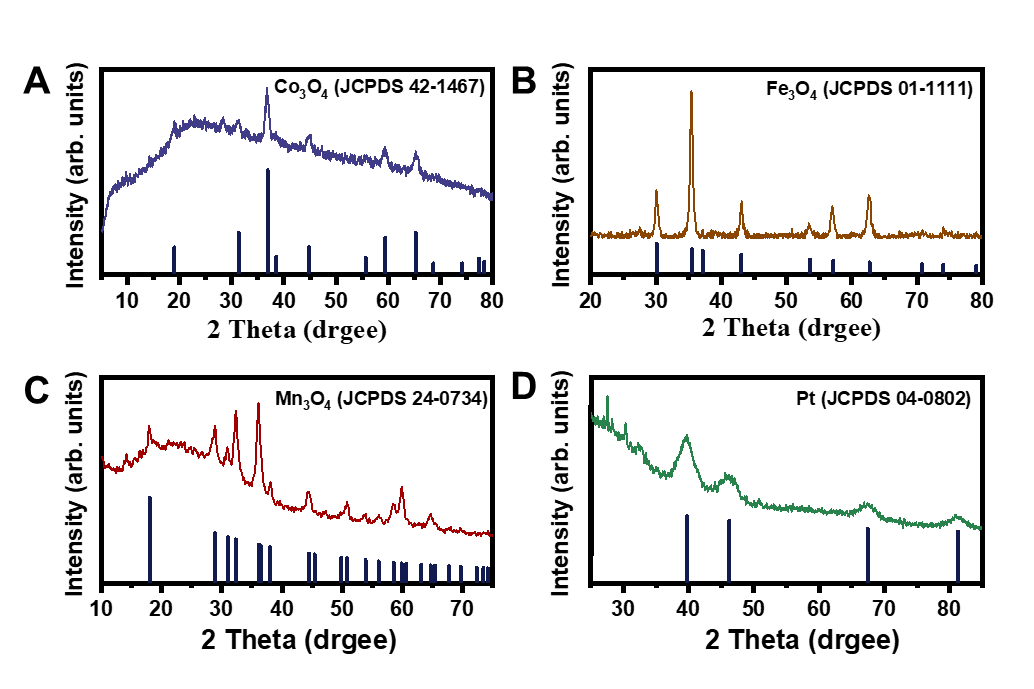
**

**Supplementary Fig. 22 XRD patterns**. (A) Co_3_O_4_, (B) Fe_3_O_4_, (C) Mn_3_O_4_, (D) Pt NPs.

**
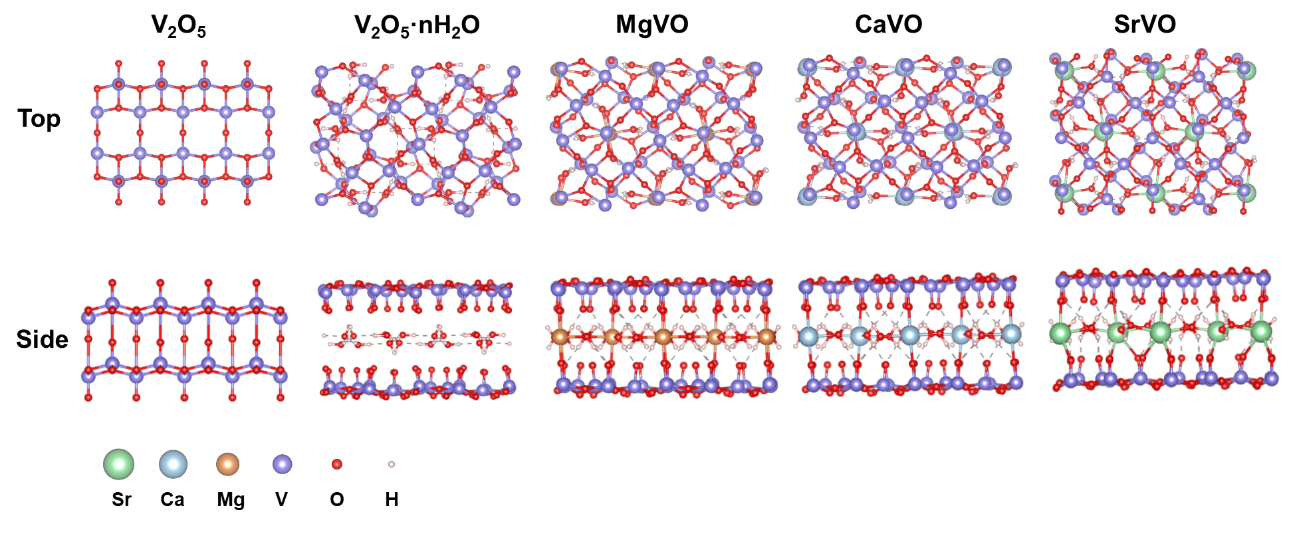
**

**Supplementary Fig. 23 Crystal models.** Top and side views of five crystal models including perfect V_2_O_5_, V_2_O_5_·nH_2_O, MgVO, CaVO, and SrVO.


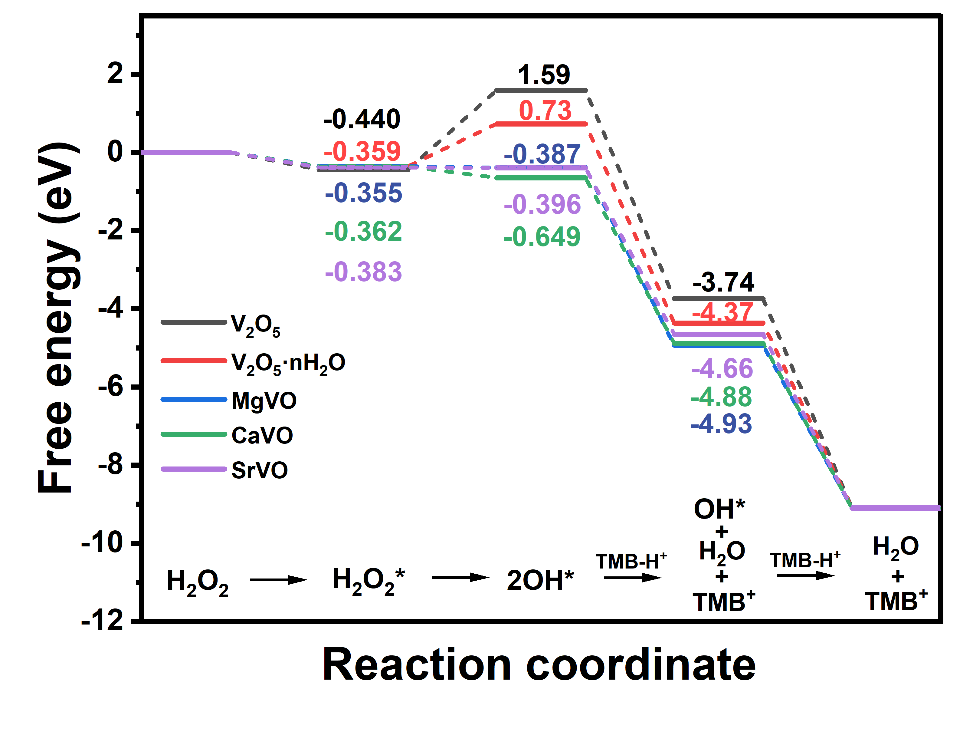


**Supplementary Fig. 24** **DFT calculation of reaction pathway.** Free energy diagram of the proposed reaction pathway with substrate (TMB) for V_2_O_5_, V_2_O_5_·nH_2_O and M_x_V_2_O_5_·nH_2_O models.


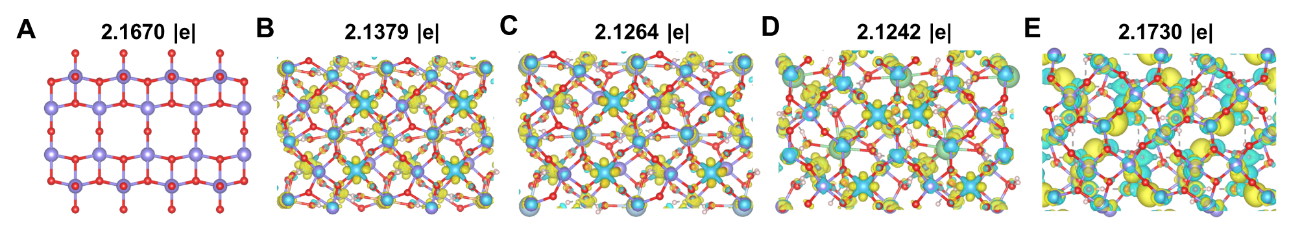


**Supplementary Fig. 25 The bader charge and differential charge analysis.** (A) V_2_O_5_, (B) MgVO, (C) CaVO, (D) SrVO and (E) V_2_O_5_·nH_2_O. (cyan and yellow represent charge depletion and accumulation, respectively)


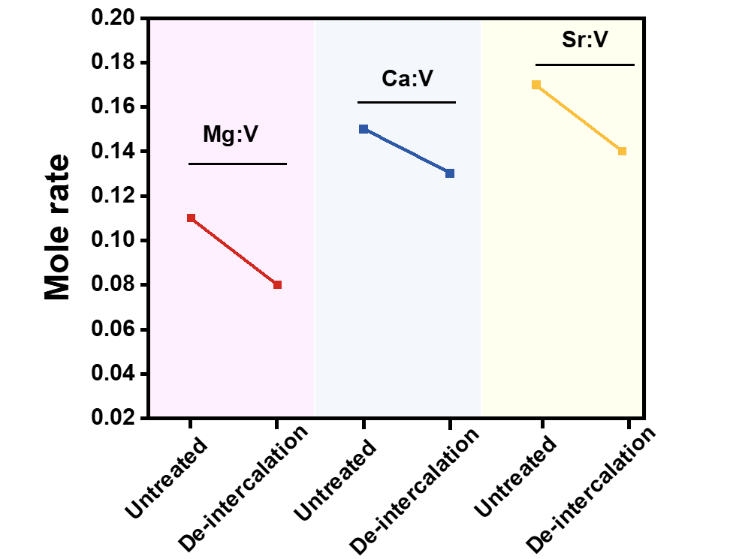


**Supplementary Fig. 26 The molar ratios of Mg/V, Ca/V, and Sr/V in M_x_V_2_O_5_·nH_2_O before and after deintercalation.**


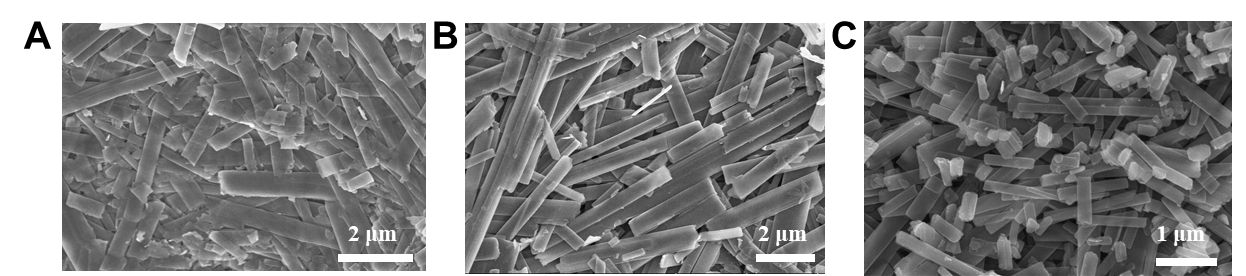


**Supplementary Fig. 27** **SEM images.** (A) MgVO, (B) CaVO and (C) SrVO after deintercalation. Representative images are shown from three independent experiments with similar results.


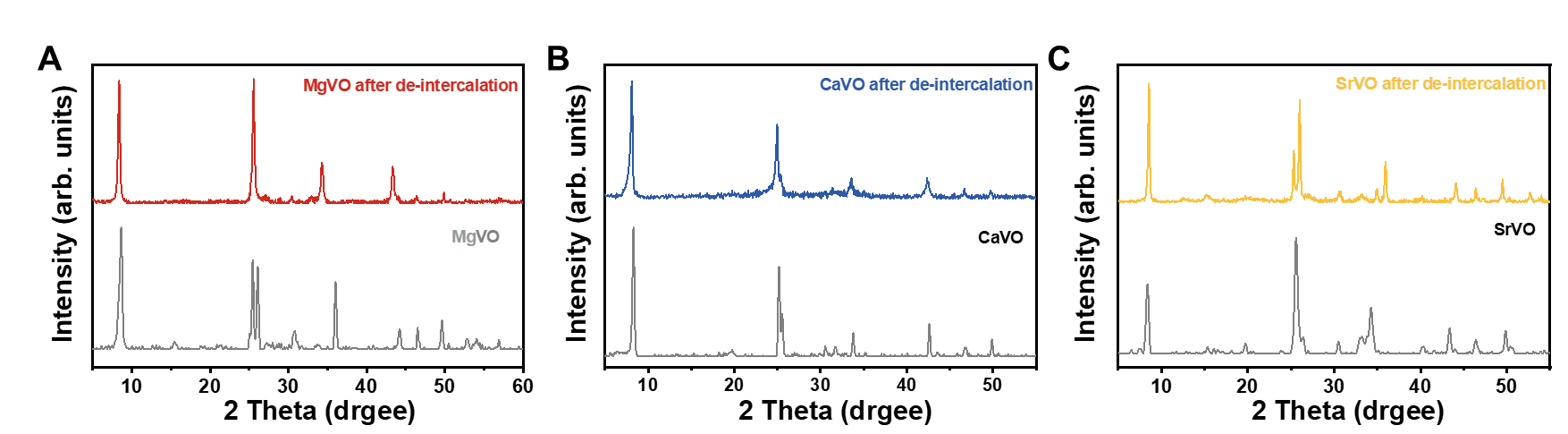


**Supplementary Fig. 28 XRD patterns.** (A) MgVO, (B) CaVO and (C) SrVO after deintercalation.


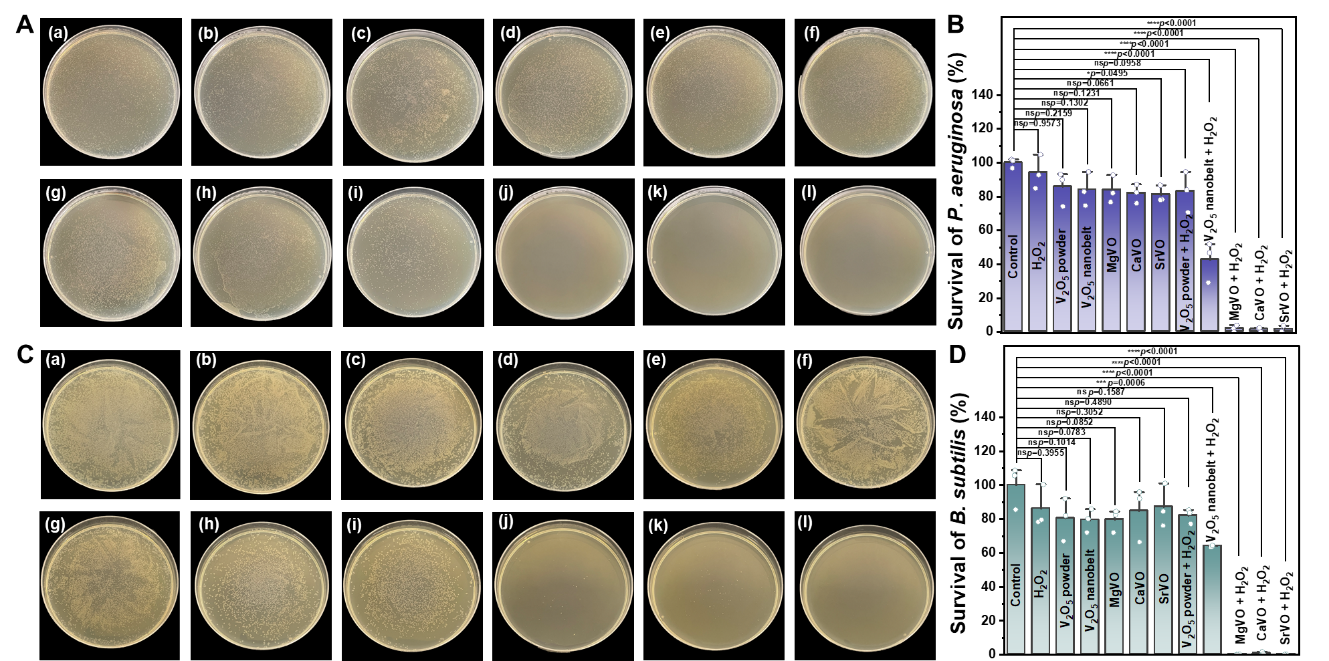


**Supplementary Fig. 29 Antibacterial properties of M_x_V_2_O_5_·nH_2_O.** Photographs of bacterial colonies against *P. aeruginosa* (A) and *B. subtilis* (C) with different treatments. Relative survival rate of *P. aeruginosa* (B) and *B. subtilis* (D) upon different treatments determined by spread plate method. (a) PBS, (b) H_2_O_2_, (c) V_2_O_5_ powder, (d) V_2_O_5_ nanobelt, (e) MgVO, (f) CaVO, (g) SrVO, (h) V_2_O_5_ powder + H_2_O_2_, (i) V_2_O_5_ nanobelt + H_2_O_2_, (j) MgVO + H_2_O_2_, (k) CaVO + H_2_O_2_, (l) SrVO + H_2_O_2_. n = 3 biologically independent samples (B, D). Data are presented as mean values ± SD (B, D). Data were analyzed by one-way ANOVA with Turkey’s multiple comparisons test, ns represents no statistical difference, *P<0.01. **P<0.001. ***P<0.0001. ****P<0.0001.


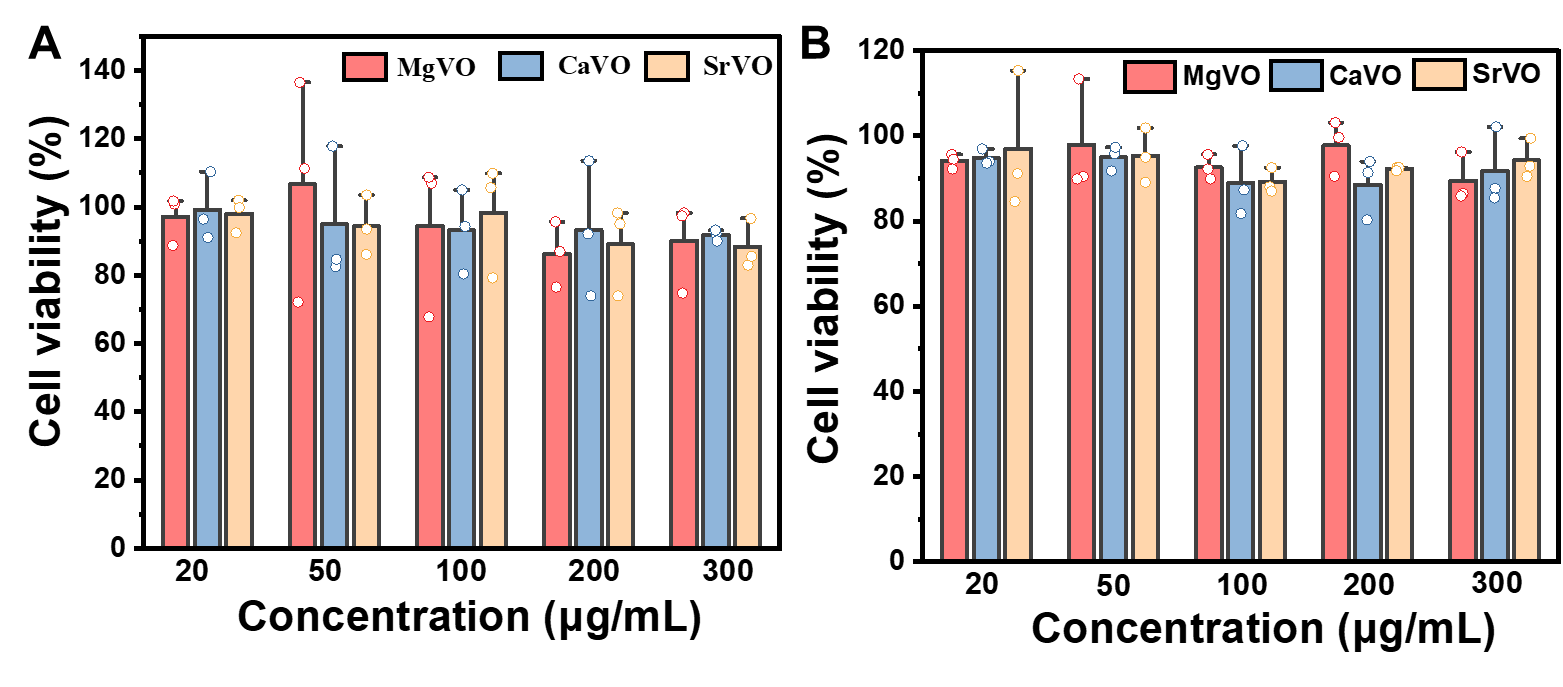


**Supplementary Fig. 30 Cytotoxicity validation of M_x_V_2_O_5_·nH_2_O.** Assay of the viability of HMEC-1 (A) and L929 (B) cells cultured with varying M_x_V_2_O_5_·nH_2_O concentrations. n = 3 biologically independent samples. Data are presented as mean values ± SD.

.
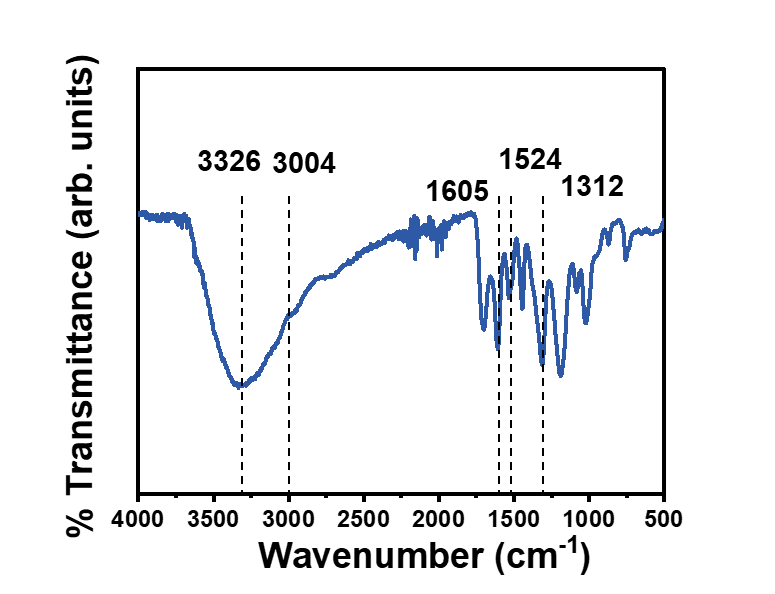


**Supplementary Fig. 31 FTIR spectra of hydrogel wound dressing.** (The peaks of 3326 and 1312 cm^-1^ are attributed to the stretching and bending vibrations of OH in TA, respectively. The peaks of 3004, 1605 and 1524 cm^-1^ correspond to C-H stretching vibration of protein in gelatin, C=O stretching vibration of amide I band and N-H bending vibration of amide II band, re-spectively.)

**
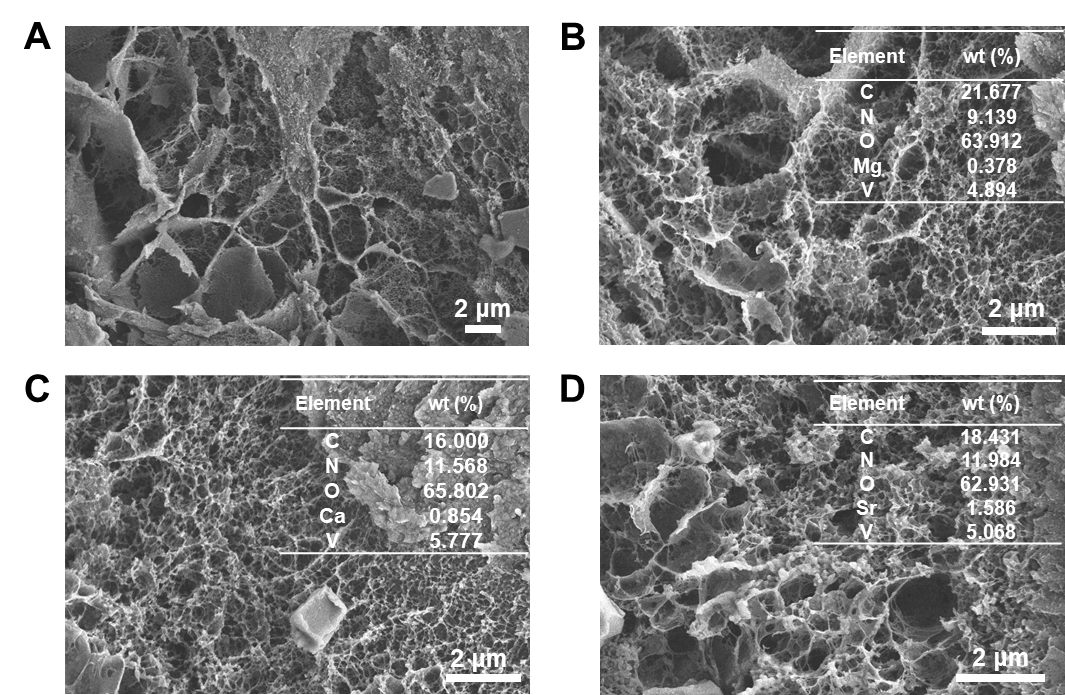
**

**Supplementary Fig. 32** **SEM images with EDX elemental quantification of functionalized wound dressing.** The SEM images of GelTA (A) and SEM images with EDX elemental quantification (insets) of Mg-GelTA (B), Ca-GelTA (C), Sr-GelTA (D). Representative images are shown from three independent experiments with similar results.

**
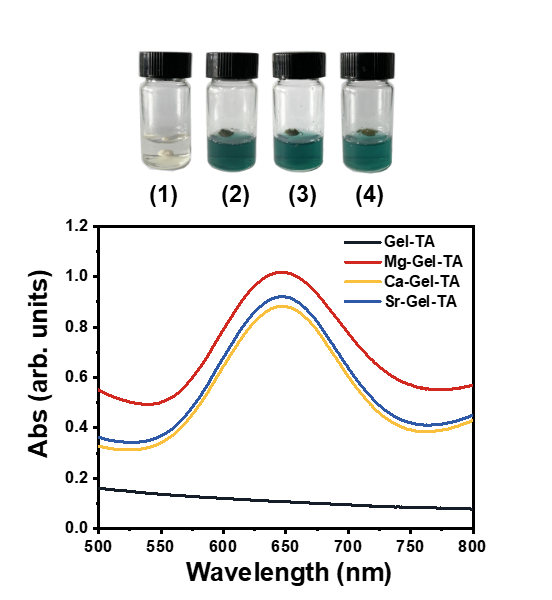
**

**Supplementary Fig. 33 Assay of peroxidase-mimicking activity of functionalized wound dressing.** Exploration of peroxidase-mimicking activity of M_x_V_2_O_5_·nH_2_O nanobelts functionalized hydrogel wound dressing.

**
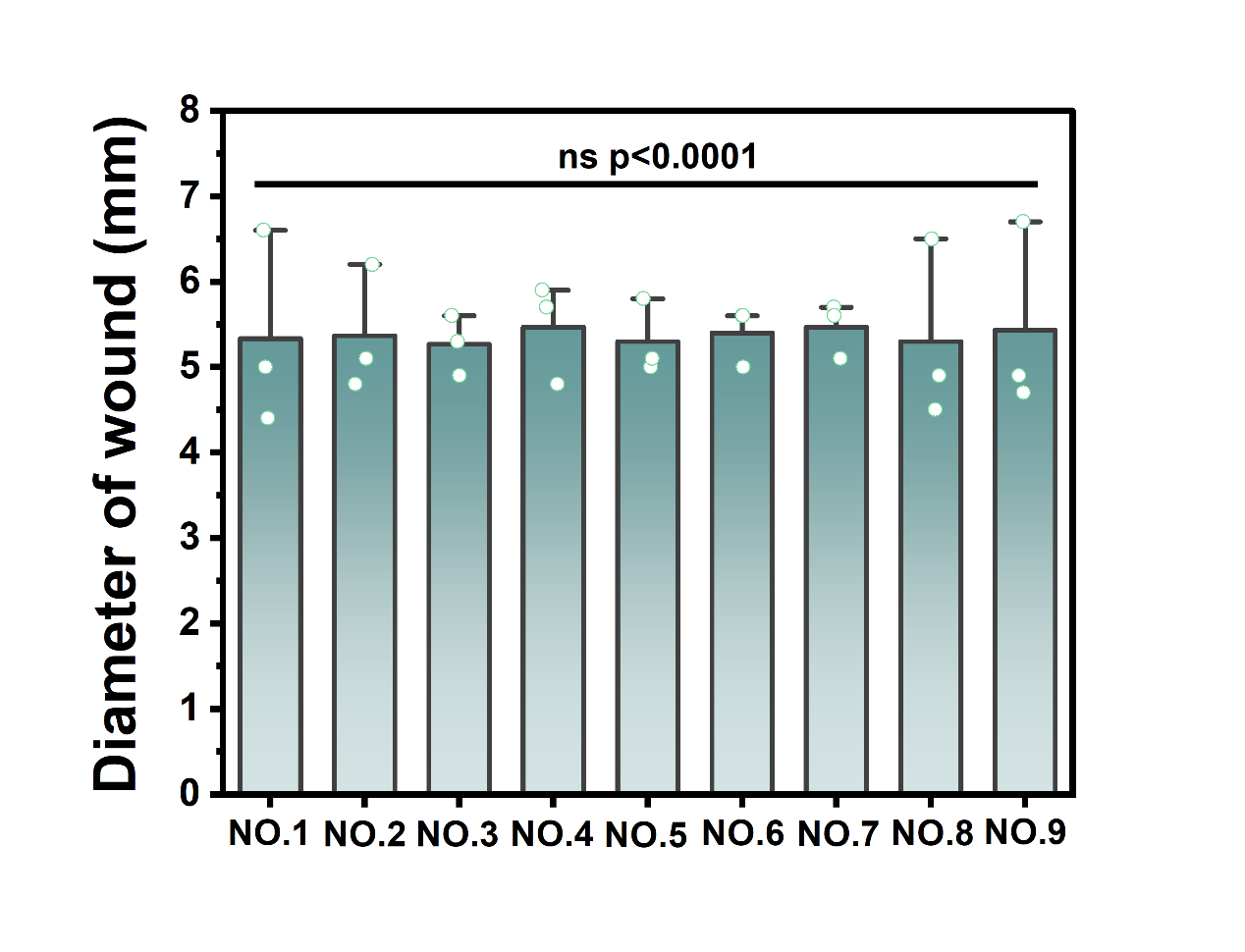
**

**Supplementary Fig. 34 The diameter of wound at day 0 measured by vernier caliper.** n = 3 biologically independent samples. Data are presented as mean values ± SD. Data were analyzed by one-way ANOVA with Turkey’s multiple comparisons test. ns represents no statistical difference.

**
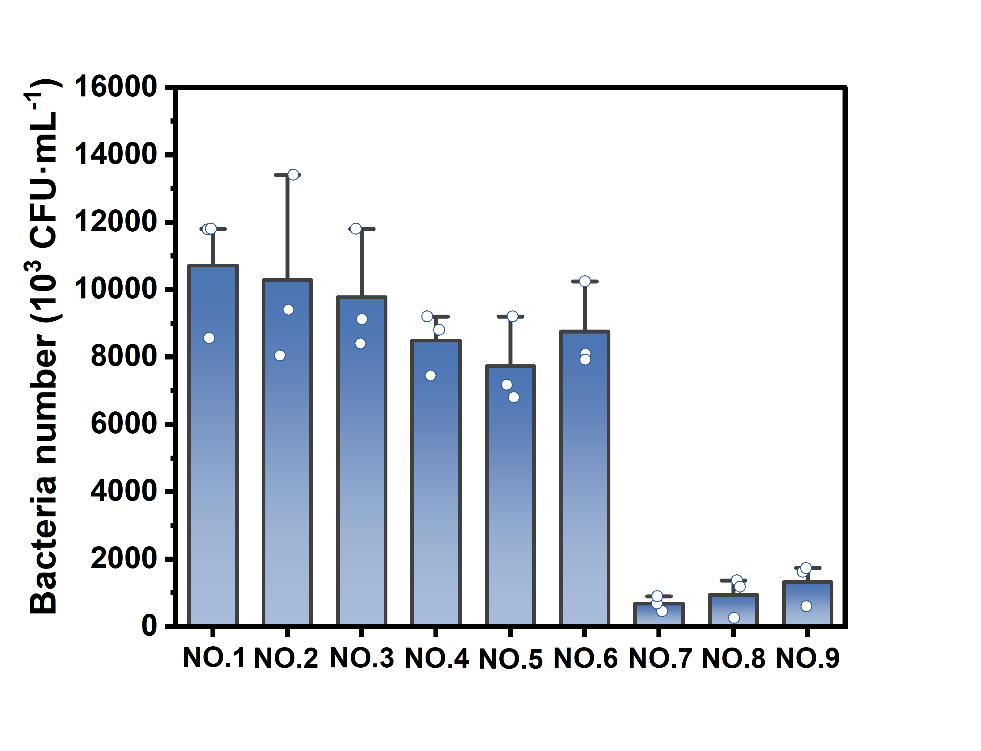
**

**Supplementary Fig. 35 Count of bacteria number.** Bacteria number in different groups after treatment at day 5. n = 3 biologically independent samples. Data are presented as mean values ± SD.

**
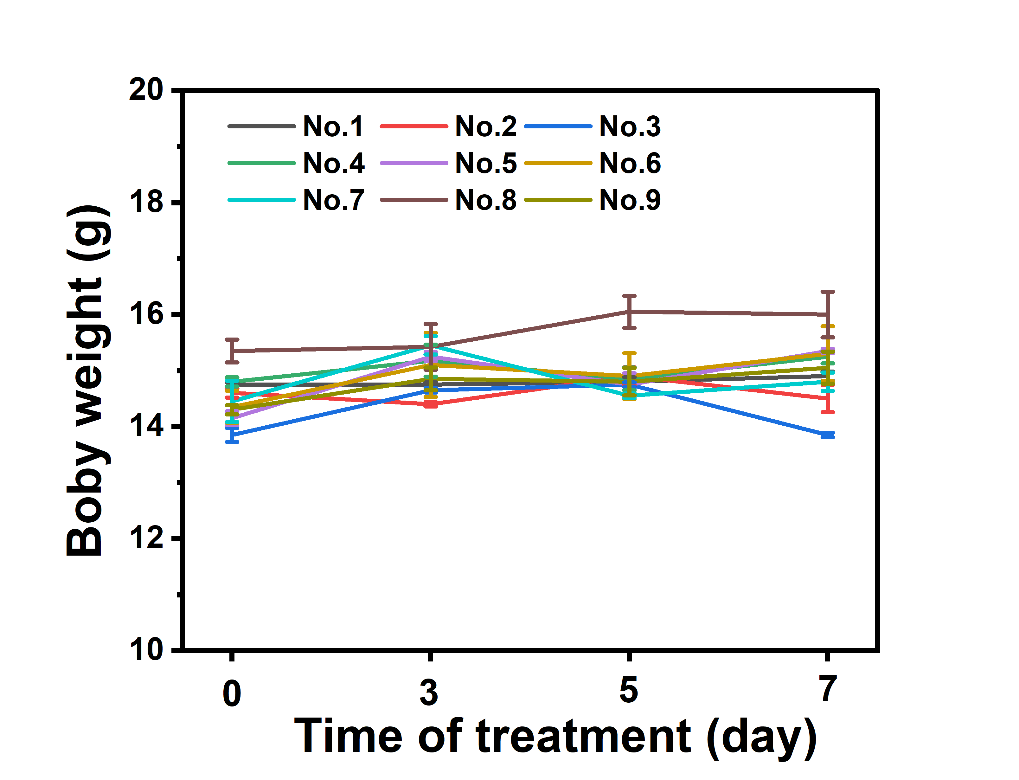
**

**Supplementary Fig. 36. Weight analysis of mice.** Changes in body weight of mice during treatment with different groups. n = 3 biologically independent samples. Data are presented as mean values ± SD.

**
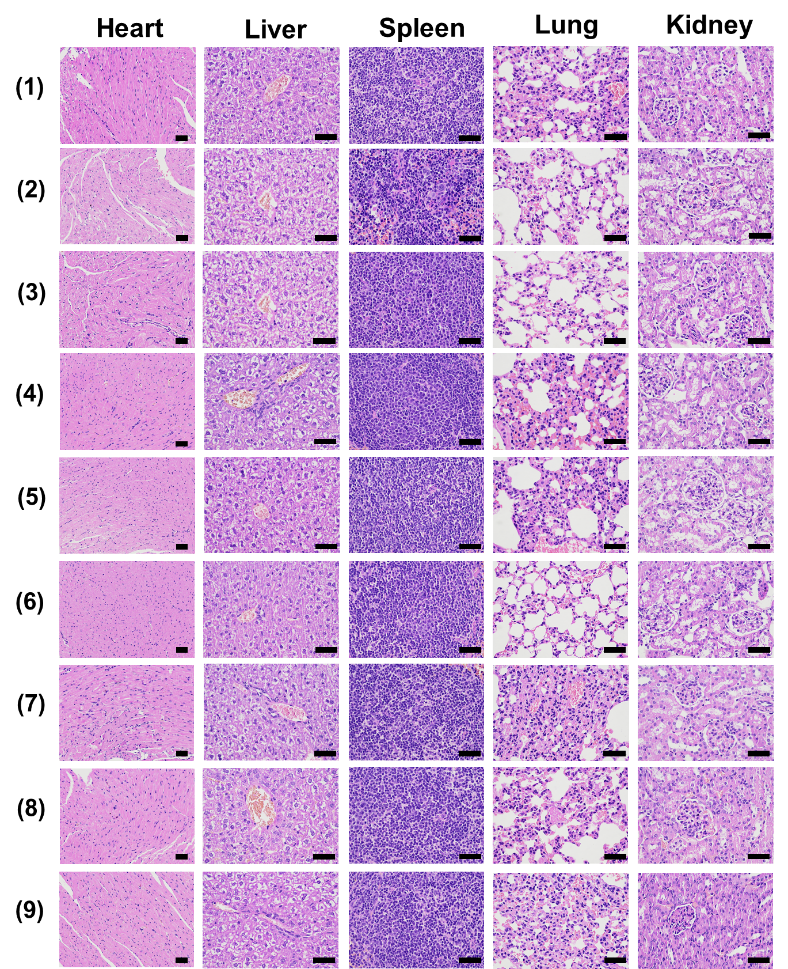
**

**Supplementary Fig. 37 Histological analysis.** H&E staining sections of major organs of mice treated with different groups. Scale bar, 100 μm.

**Supplementary Table 1** Inductively coupled plasma mass spectrometry (ICP-MS) analysis of M_x_V_2_O_5_·nH_2_O.

| **Elements**  **Samples** | **Mg (mg/L)** | **V (mg/L)** | **Mg/V (mole ratio)** |
| --- | --- | --- | --- |
| MgVO | 0.83 | 16.08 | 0.22/2 |
| **Elements**  **Samples** | **Ca (mg/L)** | **V (mg/L)** | **Ca/V (mole ratio)** |
| CaVO | 1.77 | 15.15 | 0.30/2 |
| **Elements**  **Samples** | **Sr (mg/L)** | **V (mg/L)** | **Sr/V (mole ratio)** |
| SrVO | 4.53 | 15.76 | 0.34/2 |

**Supplementary Table 2** Comparison of kinetic parameters of M_x_V_2_O_5_·nH_2_O with HPR and other reported artificial enzymes.

| **Catalyst** | **Substrate** | **K_m_ (mM)** | **V_max_ (10^-8^ M·S^-1^)** | **References** |
| --- | --- | --- | --- | --- |
| **MgVO** | TMB | 0.44 | 245 | This work |
|  | H_2_O_2_ | 7.91 | 306 |  |
| **CaVO** | TMB | 0.54 | 220 |  |
|  | H_2_O_2_ | 6.46 | 177 |  |
| **SrVO** | TMB | 0.27 | 150 |  |
|  | H_2_O_2_ | 10.32 | 148 |  |
| **Fe_3_O_4_** | TMB | 0.098 | 3.44 | Nat. Nanotechnol.,2007, 2, 577-583 |
|  | H_2_O_2_ | 154 | 9.78 |  |
| **HRP** | TMB | 0.434 | 10.0 | Nat. Nanotechnol.,2007, 2, 577-583 |
|  | H_2_O_2_ | 3.7 | 8.71 |  |

**Supplementary Table 3** Comparison of kinetic constants of M_x_V_2_O_5_·nH_2_O to H_2_O_2_ with other reported artificial enzymes.

| **Catalyst** | **V_max_ (10^-8^ M·S^-1^)** | **TON (10^-3^·S^-1^)** | **References** |
| --- | --- | --- | --- |
| **MgVO** | 306 | 123 | This work |
| **CaVO** | 177 | 73.9 |  |
| **SrVO** | 148 | 66.5 |  |
| **VO_x_** | 26.0 | 26.0 | Adv.Mater.2022, 34, 2108646 |
| **MnO_2_** | 0.6 | 0.056 | Nat. Commun. 2019, 10, 704. |
| **Mn_2_O_3_** | 101.0 | 7.979 | Nat. Commun. 2019, 10, 704. |
| **Mn_3_O_4_** | 1.3 | 0.099 | Nat. Commun. 2019, 10, 704. |
| **CoO** | 114 | 8.550 | Nat. Commun. 2019, 10, 704. |
| **CeO_2_** | 18.0 | 3.096 | Nat. Commun. 2019, 10, 704. |
| **Fe_3_O_4_** | 16.0 | 1.237 | Nat. Commun. 2019, 10, 704. |
| **CuO** | 28.0 | 2.226 | Nat. Commun. 2019, 10, 704. |
| **Co_3_O_4_** | 26.0 | 2.087 | Nat. Commun. 2019, 10, 704. |
| **Fe-N-C** | 62.0 | 3.990 | Anal. Chem. 2020, 92, 3373. |
| **Cu NPs/N-C** | 06.0 | 3.300 | Anal. Chem. 2020, 92, 3373. |
| **Co-N-C** | 16.5 | 9.582 | ACS Catal. 2020, 10, 6422. |
| **Zn-N-C** | 4.8 | 0.308 | ACS Catal. 2020, 10, 6422. |
| **Pt NCs** | 18.2 | 1.370 | ACS Appl. Mater. Interfaces. 2017, 9, 10027. |

TON=V_max_/[E], where [E] is the mole concentration of metal in the whole nanomaterials.

**Supplementary Table 4** ICP-MS analysis of M_x_V_2_O_5_·nH_2_O after deintercalation.

| **Elements**  **Samples** | **Mg (mg/L)** | **V (mg/L)** | **Mg/V (mole ratio)** |
| --- | --- | --- | --- |
| MgVO after deintercalation | 0.67 | 16.45 | 0.08/1 |
| **Elements**  **Samples** | **Ca (mg/L)** | **V (mg/L)** | **Ca/V (mole ratio)** |
| CaVO after deintercalation | 1.64 | 16.64 | 0.13/1 |
| **Elements**  **Samples** | **Sr (mg/L)** | **V (mg/L)** | **Sr/V (mole ratio)** |
| SrVO after deintercalation | 4.02 | 15.88 | 0.14/1 |
