# Supplementary material for: Biomimetic synergistic effect of redox site and Lewis acid for construction of efficient artificial enzyme
Source: Nat Commun. 2024 Jul 26;15:6315. doi: 10.1038/s41467-024-50687-1 (PMC11282276; doi:10.1038/s41467-024-50687-1)
Supplement: Supplementary file 2 — Reporting summary [file 41467_2024_50687_MOESM2_ESM.pdf]

nature portfolio

Corresponding author(s): Lu Li, Bo Tang

Last updated by author(s): Jul 11, 2024

Reporting Summary

Nature Portfolio wishes to improve the reproducibility of the work that we publish. This form provides structure for consistency and transparency in reporting. For further information on Nature Portfolio policies, see our Editorial Policies and the Editorial Policy Checklist.

Statistics

For all statistical analyses, confirm that the following items are present in the figure legend, table legend, main text, or Methods section.

n/a	Confirmed	
☐	☒	The exact sample size (*n*) for each experimental group/condition, given as a discrete number and unit of measurement
☐	☒	A statement on whether measurements were taken from distinct samples or whether the same sample was measured repeatedly
☐	☒	The statistical test(s) used AND whether they are one- or two-sided<br>*Only common tests should be described solely by name; describe more complex techniques in the Methods section.*
☒	☐	A description of all covariates tested
☐	☒	A description of any assumptions or corrections, such as tests of normality and adjustment for multiple comparisons
☐	☒	A full description of the statistical parameters including central tendency (e.g. means) or other basic estimates (e.g. regression coefficient) AND variation (e.g. standard deviation) or associated estimates of uncertainty (e.g. confidence intervals)
☐	☒	For null hypothesis testing, the test statistic (e.g. *F*, *t*, *r*) with confidence intervals, effect sizes, degrees of freedom and *P* value noted<br>*Give P values as exact values whenever suitable.*
☒	☐	For Bayesian analysis, information on the choice of priors and Markov chain Monte Carlo settings
☒	☐	For hierarchical and complex designs, identification of the appropriate level for tests and full reporting of outcomes
☒	☐	Estimates of effect sizes (e.g. Cohen's *d*, Pearson's *r*), indicating how they were calculated

Our web collection on statistics for biologists contains articles on many of the points above.

Software and code

Policy information about availability of computer code

Data collection	The data were collected on Thermo Fisher Talors F2OOS G2(TEM, HRTEM, EDS-mapping), Hitacihi Cold Field Emisson Scanning Electron Microscope (SEM), RigaKu Ultima IV X-ray diffractormeter (XRD), Escalab 250Xi (XPS), HORIBA LabRAM HR Evolution Raman Spectrometer (Raman), Therm Fisher Scientific IS50 FI-IR Spectormeter (FT-IR), Bruker EMX plus-6/1 (ESR), Bruker Dimension Icon (AFM), Shimadzu UV-2600i (UV-Vis), Thermo scientific XSeries2 (ICP-MS), Mettler Toledo TGA/DSC 3+ (TGA),Leica STELLARIS 5 confocal laser scanning microscop (CLSM). Density functional theory (DFT) calculations was conducted on Vienna ab-initio simulation package (VASP).
Data analysis	Graphpad Psism 8.0 software was used for statistical analysis. Microsoft execl 2019, Origin 2021, NanoScope Aanlysis 3.0, MDI Jade 6.0 and Digital Micrograph 3.9.1, Adobe Photoshop cc 2018, Adobe Illustrator 2022 were used to analyze the data.

For manuscripts utilizing custom algorithms or software that are central to the research but not yet described in published literature, software must be made available to editors and reviewers. We strongly encourage code deposition in a community repository (e.g. GitHub). See the Nature Portfolio guidelines for submitting code & software for further information.

Data

Policy information about availability of data

All manuscripts must include a data availability statement. This statement should provide the following information, where applicable:

- Accession codes, unique identifiers, or web links for publicly available datasets
- A description of any restrictions on data availability
- For clinical datasets or third party data, please ensure that the statement adheres to our policy

All data supporting the findings of this study are available within the article and its Supplementary Information files. Source data are provided with this paper.

Research involving human participants, their data, or biological material

Policy information about studies with human participants or human data. See also policy information about sex, gender (identity/presentation), and sexual orientation and race, ethnicity and racism.

Reporting on sex and gender	NA
Reporting on race, ethnicity, or other socially relevant groupings	NA
Population characteristics	NA
Recruitment	NA
Ethics oversight	NA

Note that full information on the approval of the study protocol must also be provided in the manuscript.

Field-specific reporting

Please select the one below that is the best fit for your research. If you are not sure, read the appropriate sections before making your selection.

☒ Life sciences ☐ Behavioural & social sciences ☐ Ecological, evolutionary & environmental sciences

For a reference copy of the document with all sections, see nature.com/documents/nr-reporting-summary-flat.pdf

Life sciences study design

All studies must disclose on these points even when the disclosure is negative.

Sample size	No sample size calculation was performed before the experiments. Sample size was determined to be 3 per each group to obtain statistically meaningful conclusion such as differences or similarities between groups. Sample size of each experiments is indicated in Figure legends.
Data exclusions	No data exclusions.
Replication	The presented experiments were at least repeated for three times independently. All attempts at replication were successful.
Randomization	All samples were randomly allocated into experimental groups
Blinding	The researcher performing the animal experiment was blind for the animal gruops. The groups were code labeled other than labeled by treatments, whcih were reveled after results analysis. Another researcher was blinded during data collection and analysis in the experiments.

Reporting for specific materials, systems and methods

We require information from authors about some types of materials, experimental systems and methods used in many studies. Here, indicate whether each material, system or method listed is relevant to your study. If you are not sure if a list item applies to your research, read the appropriate section before selecting a response.

Materials & experimental systems

n/a	Involved in the study	
☒	☐	Antibodies
☐	☒	Eukaryotic cell lines
☒	☐	Palaeontology and archaeology
☐	☒	Animals and other organisms
☒	☐	Clinical data
☒	☐	Dual use research of concern
☒	☐	Plants

Methods

n/a	Involved in the study	
☒	☐	ChIP-seq
☒	☐	Flow cytometry
☒	☐	MRI-based neuroimaging

Eukaryotic cell lines

Policy information about cell lines and Sex and Gender in Research

Cell line source(s)	HMEC-1 (CRL-3243) and L929 (CCL-1) cells were acquired commercially form ATCC.
Authentication	The cell lines purchased from ATCC have been authenticated by STR.
Mycoplasma contamination	All cell lines were tested negative for mycoplasma contamination.
Commonly misidentified lines (See ICLAC register)	No commonly misidentified cells lines were used.

Animals and other research organisms

Policy information about studies involving animals; ARRIVE guidelines recommended for reporting animal research, and Sex and Gender in Research

Laboratory animals	6-week-old BALB/c mice (wide-type) all SPF grade, fed in a barrier environment. Mice were kept in constant temperature (22℃), constant humidity (55%) and cyclic lighting (12 h light/12 h dark). The mice were inoculated with S. aureus (ATCC 6538) at the wound sites, and were euthanized after a week of treatment.
Wild animals	No wild animals were used in this study.
Reporting on sex	This study only included female mice, and we did not pay attention to the effect of gender on wound healing. Since male mice are more prone to bite wounds and dressings than female mice when fed in the same cage, we chose more docile female mice.
Field-collected samples	No field-collected samples in this study.
Ethics oversight	All animal experiments were carried out under the approval of the Animal Ethics Committee of Shandong Normal University.

Note that full information on the approval of the study protocol must also be provided in the manuscript.

Plants

Seed stocks	NA
Novel plant genotypes	NA
Authentication	NA
