# Supplementary material for: Biomimetic synergistic effect of redox site and Lewis acid for construction of efficient artificial enzyme
Source: Nat Commun. 2024 Jul 26;15:6315. doi: 10.1038/s41467-024-50687-1 (PMC11282276; doi:10.1038/s41467-024-50687-1)
Supplement: Supplementary file 4 — Description of Additional Supplementary Files [file 41467_2024_50687_MOESM4_ESM.pdf]

Description of Additional Supplementary Files

File Name: Supplementary Data 1

Description: This file contains the atomic coordinates of the studied structures.
